# Cross-Linking of Sugar-Derived Polyethers and Boronic Acids for Renewable, Self-Healing, and Single-Ion Conducting Organogel Polymer Electrolytes

**DOI:** 10.1021/acsaem.2c03937

**Published:** 2023-02-22

**Authors:** Emma L. Daniels, James R. Runge, Matthew Oshinowo, Hannah S. Leese, Antoine Buchard

**Affiliations:** † University of Bath Institute for Sustainability, Claverton Down, Bath BA2 7AY, U.K.; ‡ Department of Chemistry, University of Bath, Claverton Down, Bath BA2 7AY, U.K.; § Materials for Health Lab, Department of Chemical Engineering, University of Bath, Claverton Down, Bath BA2 7AY, U.K.

**Keywords:** xylose, bioderived polymers, self-healing gel, single-ion conductor, gel polymer electrolyte

## Abstract

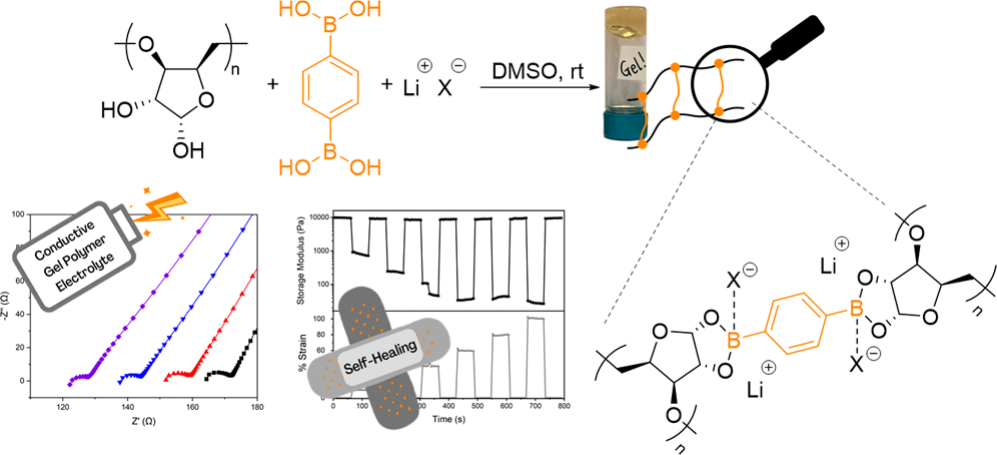

This report describes the synthesis and characterization of organogels by reaction of a diol-containing polyether, derived from the sugar d-xylose, with 1,4-phenylenediboronic acid (PDBA). The cross-linked materials were analyzed by infrared spectroscopy (FT-IR), thermal gravimetric analysis (TGA), scanning electron microscopy (FE-SEM), and rheology. The rheological material properties could be tuned: gel or viscoelastic behavior depended on the concentration of polymer, and mechanical stiffness increased with the amount of PDBA cross-linker. Organogels demonstrated self-healing capabilities and recovered their storage and loss moduli instantaneously after application and subsequent strain release. Lithiated organogels were synthesized through incorporation of lithium bis(trifluoromethanesulfonyl)imide (LiTFSI) into the cross-linked matrix. These lithium–borate polymer gels showed a high ionic conductivity value of up to 3.71 × 10^–3^ S cm^–1^ at 25 °C, high lithium transference numbers (*t*_+_ = 0.88–0.92), and electrochemical stability (4.51 V). The gels were compatible with lithium-metal electrodes, showing stable polarization profiles in plating/stripping tests. This system provides a promising platform for the production of self-healing gel polymer electrolytes (GPEs) derived from renewable feedstocks for battery applications.

## 1 Introduction

The reversible reaction of boronic acids with *cis*-1,2 or *cis*-1,3 diols to form boronate esters has been leveraged in applications such as glucose and chemosensors,^1−6^ drug delivery systems,^7−9^ and chromatography.^10−12^ When combined with polymeric materials, this reversible chemistry can generate functional, cross-linked hydro- or organogel networks. The formation of boronate ester cross-links is reversible, depending on pH, temperature, and the presence of other hydroxylated compounds.^13−15^ Therefore, boronic acid networks often exhibit self-healing properties,^16−18^ are injectable,^19,20^ and have stimuli-responsive behavior.^21^ The alginate-boronic acid hydrogel developed by Hong et al. demonstrates for example many of the desirable properties of boronic acid-diol cross-linking: it shows self-healing and high strain to failure and is responsive to pH and glucose concentration.^22^

Many of these self-healing networks are made from reaction of a boronic-acid-containing polymer (often a polymer containing phenylboronic acid (PBA) side chains) with a second, diol-containing polymer, such as poly(vinyl alcohol) (PVA), or catechol derivatives of polymers.^14,16−18,23^ Alternatively, both components can exist within the same polymer, leading to intramolecular cross-linking and gel formation.^20−22^

Cross-linking can also be achieved by reaction of a diol-containing polymer with a small molecule, a diboronic acid cross-linker. Cross-linking of PVA with borax or boric acid, for example, is well-known to easily form a gel.^24−26^ 1,4-Phenylenediboronic acid (PDBA) is soluble in many organic solvents and so can be used to synthesize various organogels. Duncan et al. demonstrated the formation of dimethyl sulfoxide (DMSO), dimethylformamide (DMF), and methanol gels from PDBA and hydrolyzed poly(vinyl acetate) (PVAc).^27,28^ Gelation between PDBA and PVA or guar is also possible, with the latter able to form hollow microspheres by the extrusion of PVA into PDBA solutions.^29,30^ Hydrogels of PDBA can also be formed indirectly, by immersion of organogels in water. Nishiyabu et al. for example combined PDBA, PVA, and a dansyl-modified boronic acid in DMSO to afford a fluorescent gel capable of the detection of aqueous copper ions.^3^ A hydrogel has also been made from the meta derivative, 1,3-phenylenediboronic acid, and catechol-derivatized poly(ethyleneglycol) (PEG).^13^

The Lewis acidity of boron allows further functionality to be installed into the networks to create ion-conductive organogels for application as gel polymer electrolytes (GPEs). GPEs are attractive materials for use in lithium-ion technologies as they show improved safety compared to liquid electrolytes while maintaining high ionic conductivity relative to inorganic and solid polymer electrolytes (SPEs).^31,32^ Indeed, boron atoms possess vacant p-orbitals which can coordinate to Lewis bases, such as hydroxide anions.^12,33^ This forms a new sp^3^, anionic, tetrahedral boron center, immobilized within the polymer structure creating a single-ion conducting effect. Therefore, only the cations can dissociate through the polymer matrix, resulting in increased ionic conductivity and transference numbers close to unity. The electron-deficient sp^2^ boron center can also coordinate anions of lithium salts, acting as an “anion-trap”, enhancing dissociation of cation–anion pairs to further improve the conductivity and transference number.^33,34^

Several groups have exploited the single-ion conducting^35−40^ and anion-trapping^41−44^ capabilities of boron in polymer electrolyte systems. Among these, Mecerreyes and co-workers have recently reported single-ion conducting GPEs with high ambient ionic conductivity (0.71 × 10^–3^ S cm^–1^ at 25 °C) and transference numbers of up to 0.85 based upon lithium borate methacrylate polymers.^45,46^ Meanwhile, Chen et al. have developed a hydrogel electrolyte through the copolymerization of methacrylate and acrylamide monomers in the presence of borate. The resulting GPE showed excellent ambient ionic conductivity of 4.5 × 10^–3^ S cm^–1^ while being able to self-heal during to the dynamic nature of its borate-diol cross-links.^47^ The GPE developed by Shim et al. shows a similarly high ionic conductivity of 4.2 × 10^–3^ S cm^–1^ at 30 °C. This GPE, which is based upon a semi-interpenetrating network containing poly(vinylidene fluoride) (PVDF) and cross-linkers containing ethylene oxide chains and anion-trapping boron groups, also shows a high transference number of 0.82 due to the introduction of the boron moieties.^31^ However, these examples, and most examples of boronic acid hydro- or organogels, rely on polymers derived from petrochemicals.

Our group,^48−52^ among many others,^53−56^ has identified monosaccharides as a promising sustainable feedstock for polymer synthesis, owing to their low cost, low toxicity, and high abundance as well as their potential for pre- or postpolymerization functionalization, due to the presence of multiple hydroxy groups.^57−59^ Herein, we report a novel organogel system from the cross-linking of a sugar-derived polyether with PDBA. The polyether, previously studied in our group, is derived from d-xylose and can be modified by postpolymerization to reveal *cis*-1,2-diols along the polymer backbone.^50^ These hydroxy groups have been utilized to generate DMSO organogels with tunable rheological properties and self-healing capabilities. Cross-linking in the presence of a lithium salt exploited the Lewis acidity of the boron-based cross-links and produced conductive organogels, which have been further investigated as a single-ion conducting gel polymer electrolyte.

## 2 Results and Discussion

### 2.1 Organogel Synthesis

**Scheme 1:**
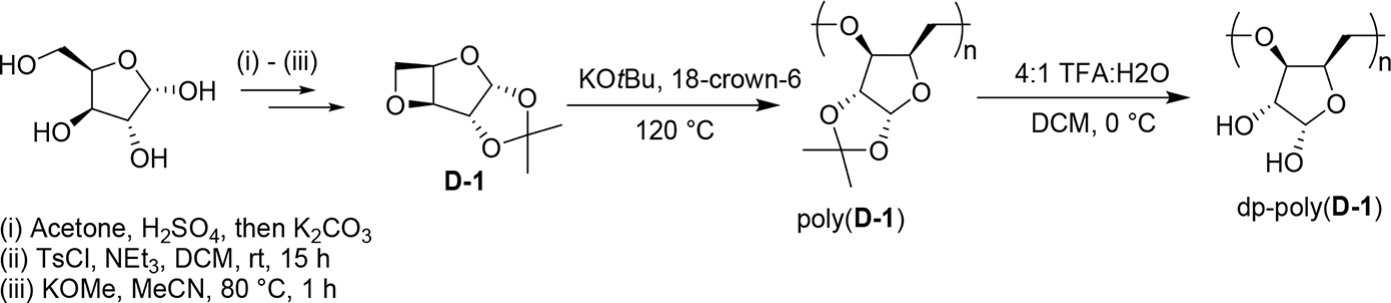
Synthesis of Monomer **d****-1**, Subsequent Anionic Ring-Opening Polymerization, and Post-Polymerization Acetal Deprotection to Yield Hydroxy-Polymer, dp-poly(d**-1**)

A sugar-derived, diol-containing polymer was synthesized through postpolymerization modification of a polyether derived from d-xylose (Scheme 1). As previously reported,^50^ an oxetane-type monomer, **d****-1**, was synthezised in three steps from d-xylose. Anionic ring-opening polymerization (ROP) of d-1 with potassium *tert*-butoxide and 18-crown-6, in a monomer:KOtBu:18-crown-6 ratio of 100:1:1, yielded a regioregular polyether, poly(**d****-1**), with *M*_n,SEC_ of 12,500 g mol^–1^ (*Đ*_M_ = 1.23; vs poly(lactide) standards in THF). By acid hydrolysis, near quantitative deprotection of acetal groups was achieved (>95% deprotection), yielding DMSO and water-soluble polyethers, dp-poly(**d****-1**), with *M*_n,SEC_ of 20,000 g mol^–1^ (*Đ*_M_ = 1.21; vs poly(methyl methacrylate) standards in DMAc/LiBr).

**Scheme 2:**
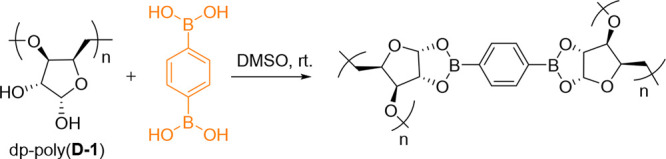
Cross-Linking of dp-poly(**d****-1**) and 1,4-Phenylenediboronic Acid (PDBA)

**Figure 1:**
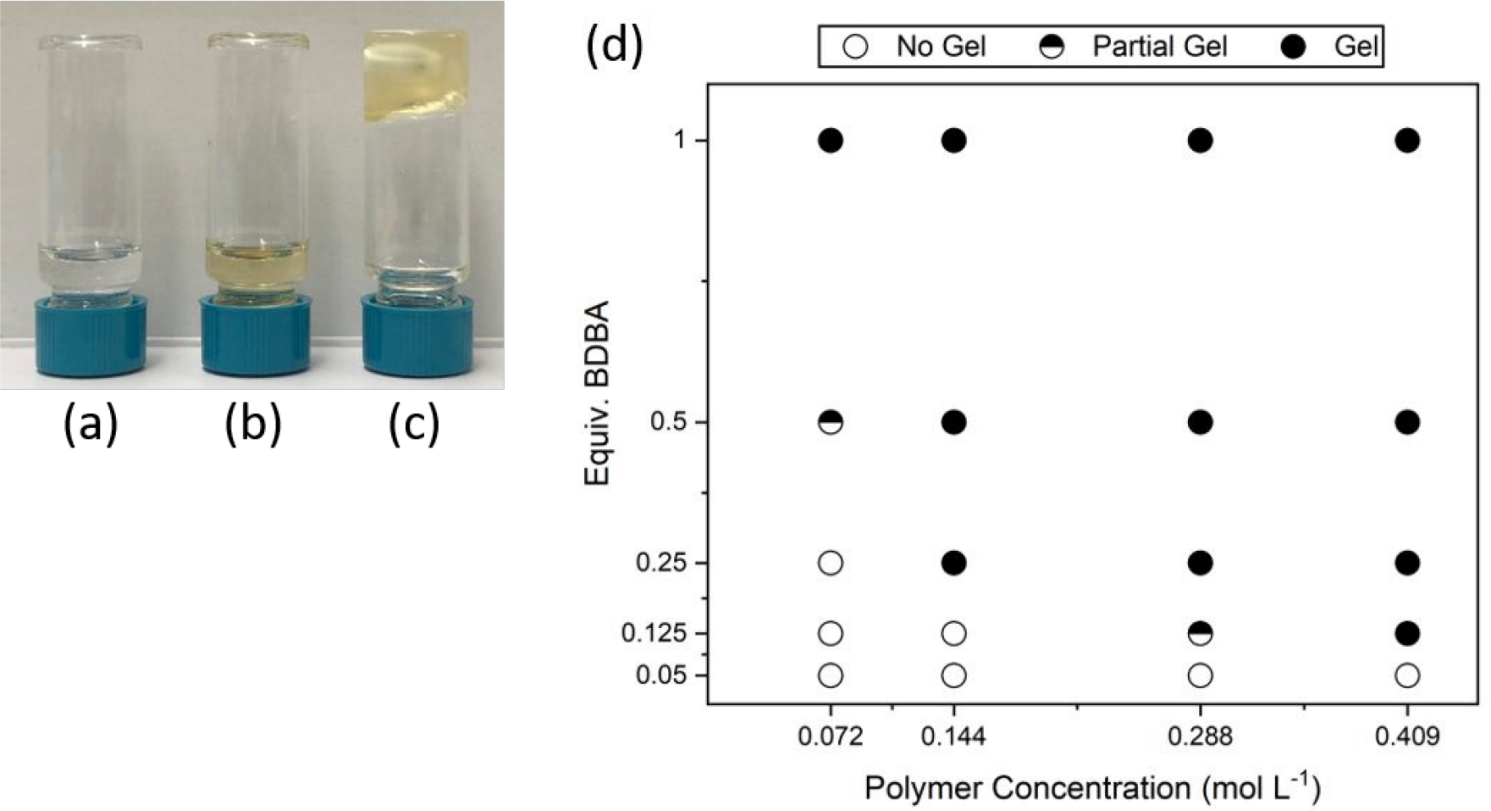
Vial inversion tests of (a) a 24 mg mL^–1^ solution of PDBA in DMSO, (b) a 0.287 mol L^–1^ solution of dp-poly(**d****-1**), and (c) a reaction between dp-poly(**d****-1**) and PDBA observed over several hours. (d) Phase diagram of the dp-poly(**d****-1**)/PDBA system in DMSO at room temperature.

The newly revealed hydroxy groups in dp-poly(**d****-1**) could then be reacted with difunctional boronic acids to form cross-linked organogels. 0.5 equiv of PDBA (with respect to the deprotected monomer unit, assuming quantitative acetal deprotection) was added to a 0.287 mol L^–1^ solution of dp-poly(**d****-1**) in DMSO (Scheme 2). When stirred at room temperature (rt), the formation of a gel occurred within 5 min, as proved qualitatively via a vial inversion test (Figures 1 a–c). Reaction mixtures in the absence of either dp-poly(**d****-1**) or PDBA remained liquid. By altering either the equivalents of PDBA or the concentration of polymer in solution, the conditions required for gelation were found (Figure 1d). The formation of a gel, as determined by the vial inversion test, is more likely in samples with high polymer concentrations and PDBA equivalents (SI, Figures S11–S14). In some cases, gel-like material was observed, but vial inversion was not successful. Hence, a partial gel event was defined.

The equilibrium between boronate esters and diols/boronic acids is known to be pH dependent, with dissociation into free boronic acid and diols favored at pH values below the p*K*_a_ of the boronic acid.^7,13,16,21,30,41^ As expected, the pH reduction of the gel solutions below 2 by addition of hydrochloric acid disrupted cross-linking and caused dissolution of the polymer network, as qualitatively indicated by failure of the vial inversion test. Similarly, gels were not stable under basic conditions, with dissolution occurring above pH 13. Small amounts of gel could be reformed by addition of acid to the basic solution (Figure S15).

**Figure 2:**
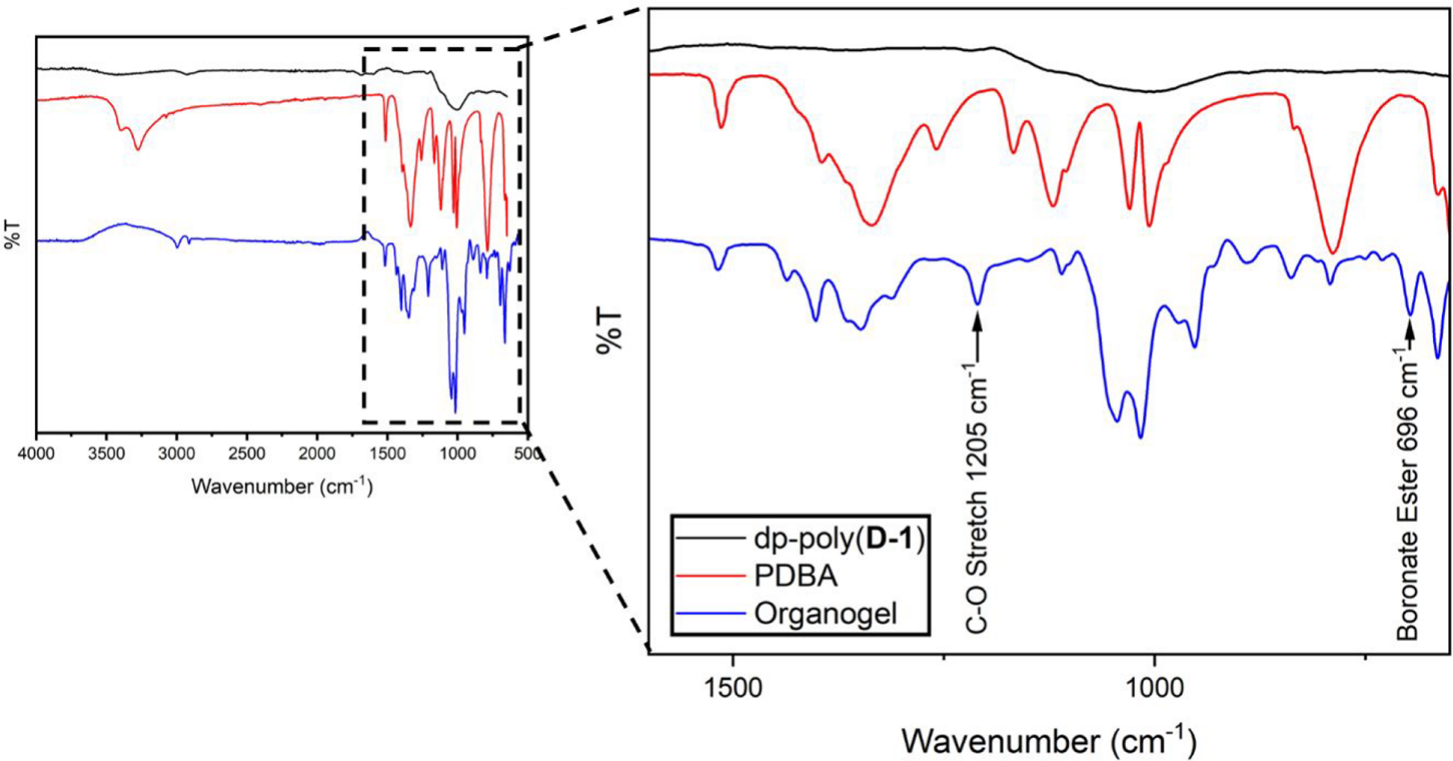
FT-IR spectra of dp-poly(**d****-1**) (black), PDBA (red), and organogel (blue) made from dp-poly(**d****-1**) (0.287 mol L^–1^) and 0.5 equiv of PDBA (with respect to monomer unit).

FT-IR analysis revealed several new chemical environments in the gels, when compared to dp-poly(d-**1**) and PDBA. Particularly, cross-linking was evidenced by a new signal at 696 cm^–1^ corresponding to new boronate ester bonds, and at new C–O stretching frequencies at 1205 cm^–1^ (Figure 2), the intensity of the latter increases with the degree of cross-linking (Figures S16–S18). However, the extent of cross-linking cannot be determined, as after gelation the materials were insoluble in all common solvents, hindering NMR analysis. Monosubstituted moieties, in which PDBA reacts with just one diol unit as shown in Scheme S1, may also be present within the organogels.

### 2.2 Rheology and Self-Healing Properties

**Figure 3:**
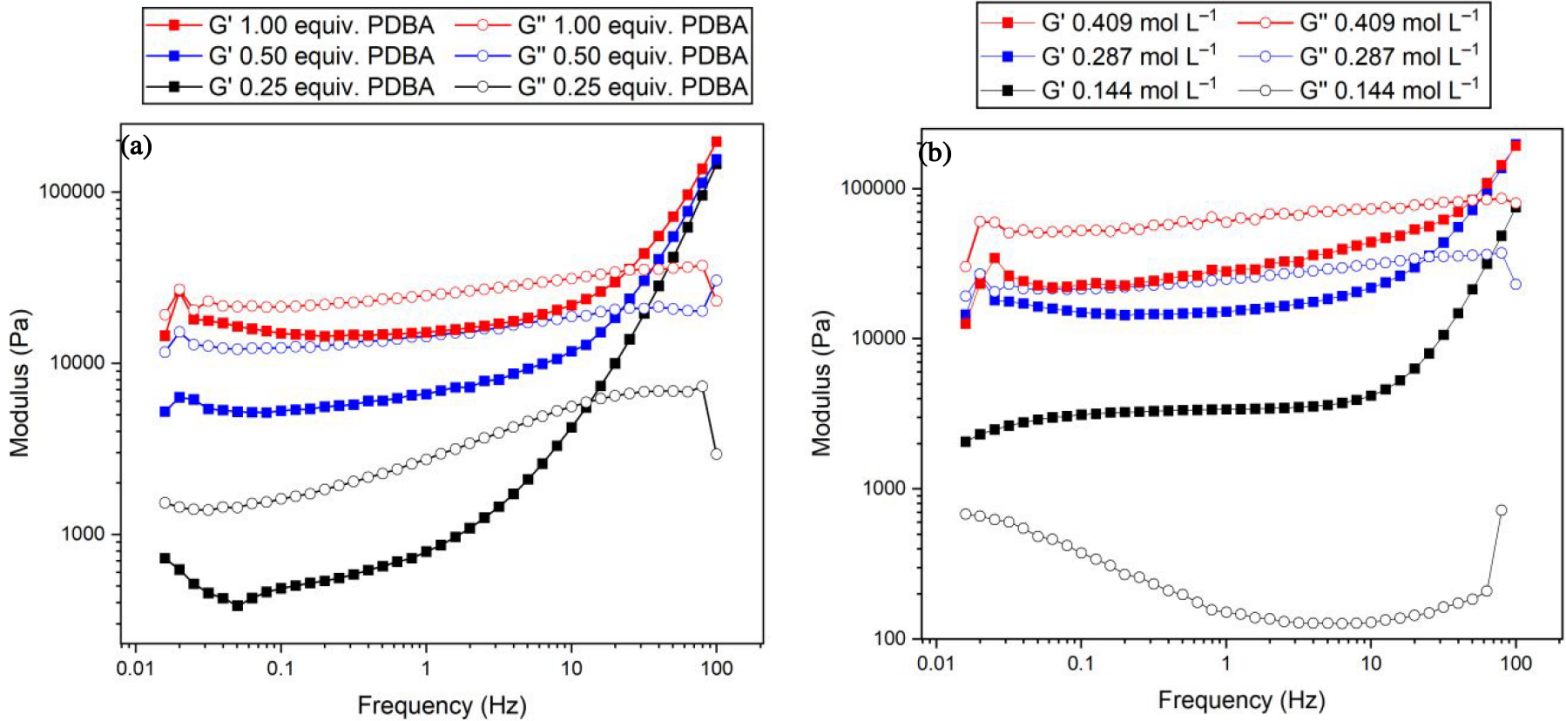
(a) Dynamic frequency sweep of organogels made from dp-poly(**d****-1**) (0.287 mol L^–1^) and increasing PDBA equiv. (b) Dynamic frequency sweep of organogels made from dp-poly(**d****-1**) (increasing concentrations) and PDBA (1 equiv).

Rheology was employed to gain further insight into the organogel materials. Frequency sweep experiments from 0 to 100 Hz showed most materials to be viscoelastic (Figure 3a). At lower frequencies, typically less than 13 Hz, the storage modulus (*G*′) is less than the loss modulus (*G*″). Beyond a cross-over frequency (ω_c_, 13.5–49.6 Hz), a gel-like region exists in which *G*′ is greater than *G*″. Looking at the 0.287 mol L^–1^/PDBA (0.25 equiv.) system in detail, *G*′ (793 Pa at 1 Hz) < *G*″ (2737 Pa at 1 Hz) was observed at rotating frequencies below 13.5 Hz, while *G*′ (9963 Pa at 20 Hz) > *G*″ (6648 Pa at 20 Hz) at frequencies greater than 13.5 Hz (Figure 3a, black trace). Such frequency-dependent moduli, with *G*′ > *G*″ only at higher frequencies, indicate the presence of dynamic cross-links.^16^ At long time scales (low frequencies) the network can reorganize with viscous behavior, while at short time scales (high frequencies) the cross-linked network is more rigid, with elastic behavior.^42^ The organogel stiffness depends on its composition, with increased PDBA content yielding increased storage and loss moduli across all frequencies due to higher degrees of cross-linking. Similar trends have been observed in other PDBA organogel systems.^27^ The crossover frequency also increased as samples became more cross-linked, occurring at 13.5, 22.3, and 24.6 Hz for materials made with 0.25, 0.50, and 1.00 equiv of PDBA, respectively. The polymer concentration also affected the rheological behavior of the cross-linked materials. Those made with 0.144 mol L^–1^ polymer solutions exhibit true gel behavior, with *G*′ > *G*″ across all frequencies (Figure 3b).^22^ This polymer concentration produced materials with consistent gel-like behavior, irrespective of the amount of PDBA (Figures S22–S24).

**Figure 4:**
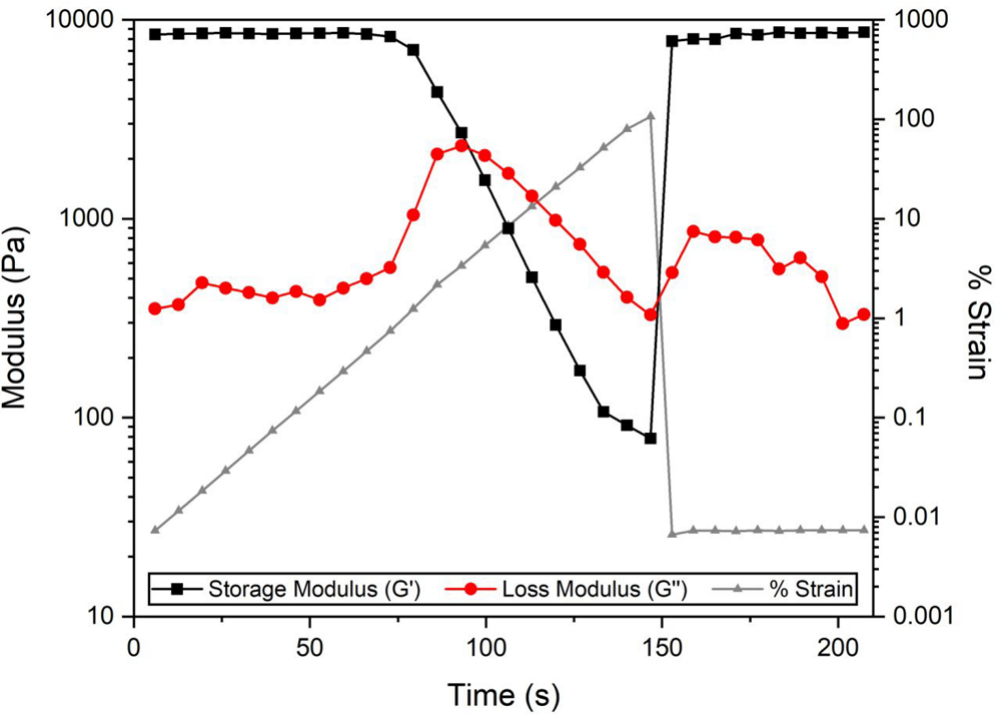
Change in moduli during a strain ramp of an organogel (0.144 mol L^–1^ dp-poly(**d****-1**), 0.50 equiv of PDBA) measured at a frequency of 1 Hz. The percentage strain was increased from 0.007% to 100%; then strain was released; and the moduli were measured at 0.007% strain.

The self-healing ability of the system was also investigated. An organogel made from a 0.144 mol L^–1^ solution of dp-poly(**d****-1**) with 0.50 equiv of PDBA was chosen for this analysis, as it showed gel-like behavior across all frequencies and demonstrated relatively high *G*′ and *G*″ values. First, the critical strain required to break the cross-linking network was identified by increasing the applied strain on 1 Hz oscillatory measurements. After 5% strain, the material showed liquid behavior, with *G*″ > *G*′, indicating disruption of the gel network (Figure S26). Upon strain release (back to 0.007%), the cross-links were able to reform, and the original moduli values were immediately restored (Figure 4). The organogel showed a *G*′ of 8457 Pa and *G*″ of 352 Pa before the application of strain. After release, the material recovered its gel-like behavior (*G*′ > *G*″), exhibiting average moduli of 8452 and 622 Pa for *G*′ and *G*″, respectively.

**Figure 5:**
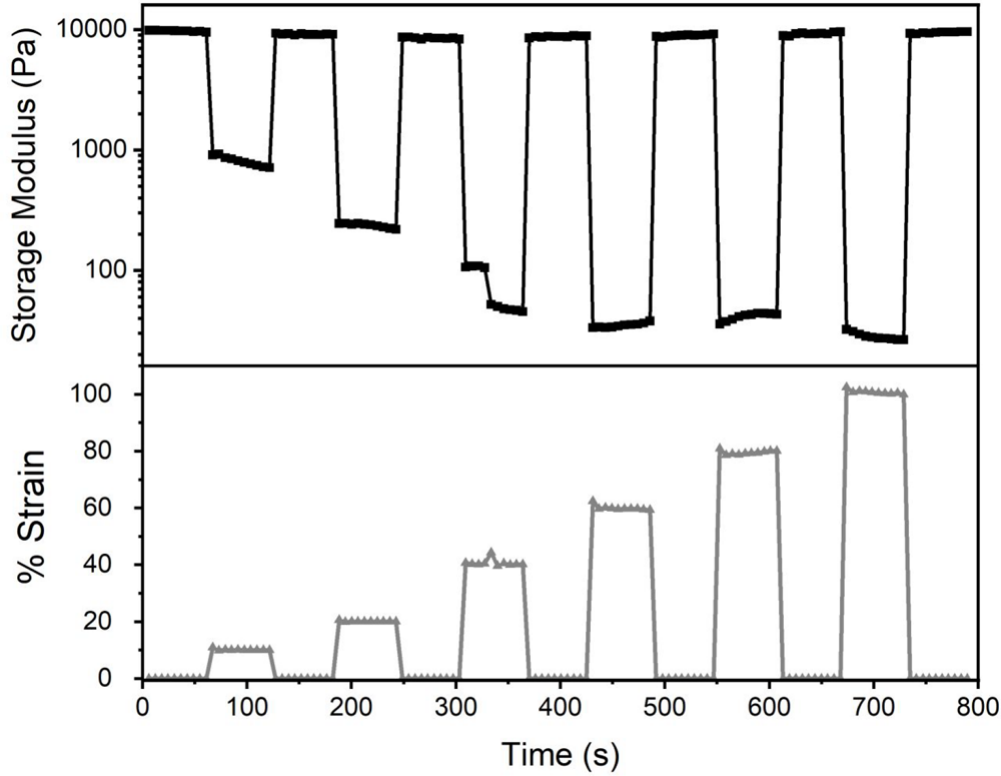
Gel fracture and self-healing in sequential strain steps. Top shows the measured storage moduli at each strain step, as described in the bottom plot (0% → 10% → 0% → 20% → 0% → 40% → 0% → 60% → 0% → 80% → 0% → 100% → 0%, measured at 1 Hz).

The ability of the organogel to self-heal was further demonstrated in step-strain measurements performed at 1 Hz (Figure 5). The percentage strain was varied in sequential strain steps: 0% to 10% to 0% to 20% to 0% to 40% to 0% to 60% to 0% to 80% to 0% to 100% to 0%. Each time the strain was relaxed (up to 80% strain), the material fully recovered its original storage modulus. Even after release of the highest strains (80 and 100% strain), 84 and 74% of the original storage, modulus was recovered, with average values of 8640 and 7810 Pa, respectively. These results show that the material quickly recovers its structural properties after experiencing mechanical strain.

### 2.3 Lyophilized Gel Microstructure and Thermal Properties

**Figure 6:**
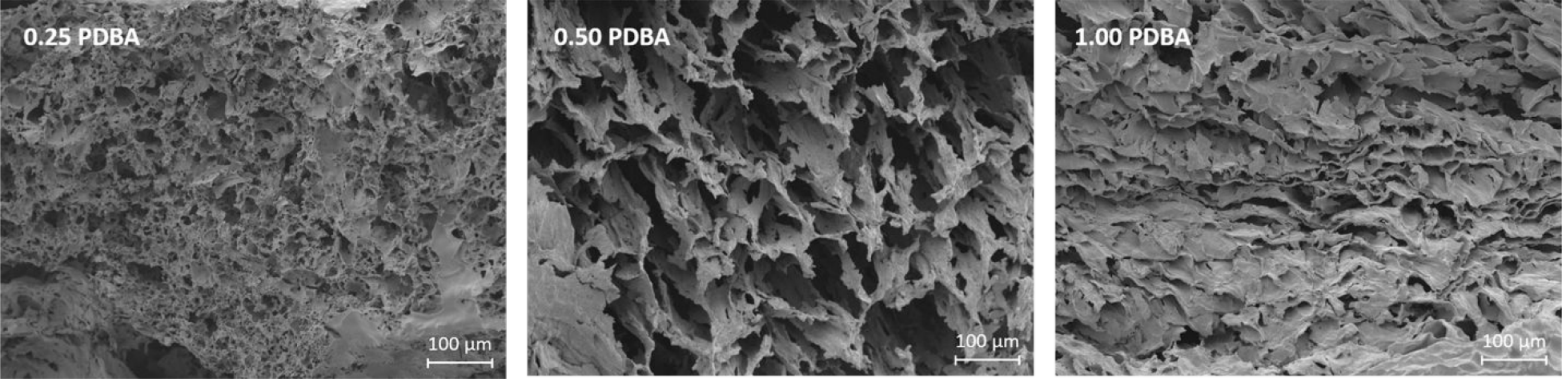
FE-SEM imaging of lyophilized organogels made from 0.144 M solutions of dp-poly(**d****-1**) in DMSO with 0.25–1.00 equiv of PDBA.

To investigate the microstructure of the gel materials, samples made from a 0.144 mol L^–1^ solution of dp-poly(**d****-1**) with 0.25–1.00 equiv of PDBA were analyzed by field emission scanning electron microscopy (FE-SEM). To remove DMSO from the system, the gels were first flash frozen in liquid nitrogen and then dried under vacuum in a process akin to freeze-drying. Materials exhibited large, irregular pores throughout. The microstructure showed a dependency on the cross-linking density (Figure 6). Cross-sectional imaging of the 0.25 material showed many globular pores, of up to 0.124 mm in length (in an observed area of 0.474 mm^2^). Increasing the proportion of PDBA to 0.50 equiv formed a less dense structure with directional, elongated pores (up to 0.415 mm in length, observed from 4.226 mm^2^). Further increase of cross-linking density produced a denser structure, with fewer observable pores (up to 0.118 mm observed in 0.473 mm^2^, also see Figures S35–S38). This structure could be due to more prominent monosubstitution of polymer chains (see Scheme S1).

**Figure 7:**
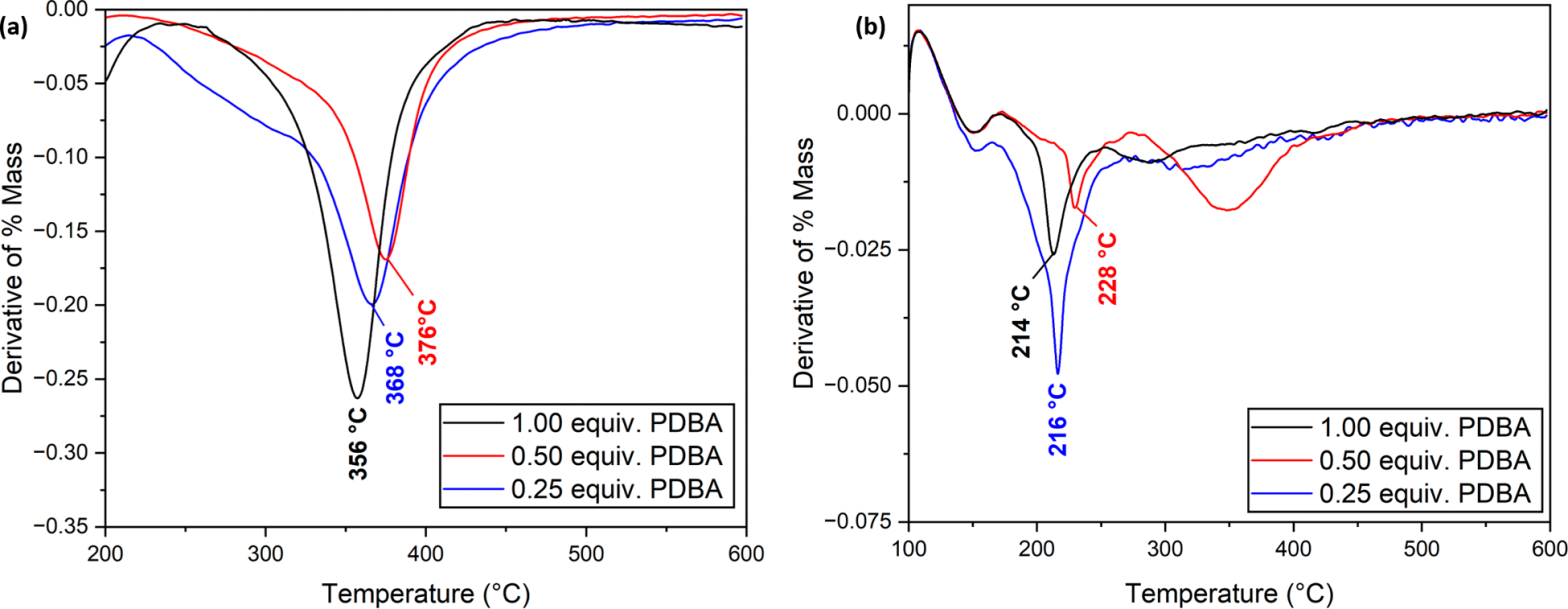
TGA of (a) lyophilized and (b) nonlyophilized organogel samples made from dp-poly(**d****-1**) and PDBA with a polymer concentration of 0.144 mol L^–1^ and PDBA equiv of 0.25–1.00. Annotations of the *T*_d,max_ values of each sample.

The thermal characteristics of the lyophilized gels were analyzed. Compared to the non-cross-linked dp-poly(**d****-1**), thermal gravimetric analysis (TGA) of dried gel samples showed that cross-linking increased thermal stability. The sample with 0.25 equiv of PDBA showed a *T*_d,max_ of 368 °C, compared to 272 °C for dp-poly(**d****-1**). This increased thermal stability is expected from the introduction of cross-linking and the presence of rigid phenyl rings. Increasing the amount of PDBA to 0.50 equiv provides more cross-linking opportunities, which is reflected by a further increase in the onset of thermal degradation to *T*_d,max_ = 376 °C. As the amount of PDBA was increased further to 1.00 equiv, the onset of degradation decreased slightly, to 356 °C (Figure 7a). This change could again be explained by an excess of PDBA favoring monosubstitution of dp-poly(**d****-1**), leaving more diol moieties along the polymer chains. The presence of diol moieties is known to reduce the thermal stability of this polyether (*T*_d,max,poly(d-1)_ = 372 °C vs *T*_d,max,dp-poly(d-1)_ = 272 °C, Figures S39–S40);^50^ hence, monosubstitution would decrease the observed *T*_d,max_.^54^ Most cross-linked materials showed an absence of glass transition, crystallization, and melting events by differential scanning calorimetry (DSC, Figures S49–S51), although a small glass transition can be observed in the material made with 0.50 equiv of PDBA (*T*_g_ = 112 °C, Figure S47).

Nonlyophilized materials were also analyzed by TGA. A similar trend to the lyophilized materials was observed: the thermal stability increased as the amount of cross-linking increased until monosubstitution became favored at high PDBA equiv (Figure 7b). TGA analysis of nonlyophilized materials also allowed the gel solvent content to be quantified. Samples were held at 100 °C for two hours to remove all the DMSO solvent (Figures S44–S46), resulting in 75, 68, and 71% mass loss for materials (and therefore mass solvent percentage) made with 0.50, 0.50, and 1.00 equiv of PDBA, respectively.

### 2.4 Gel Polymer Electrolytes and Electrochemical Properties

**Scheme 3:**
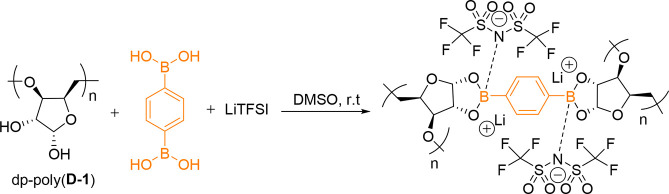
Cross-Linking of dp-poly(**d****-1**) and PDBA in the Presence of a Lithium Salt (LiTFSI)

As well as facilitating gelation and self-healing, cross-linking with boronic acids allows additional functionality to be installed via the empty p-orbital of boron. After reacting with the diols of dp-poly(**d****-1**), the boron center is sp^2^ hybridized and can coordinate with an additional nucleophilic group to become anionic and sp^3^ hybridized.^31^ Balancing the charge with lithium counter cations introduces conductivity. Thus, cross-linking dp-poly(d**-1**) with PDBA (0.5 equiv) in the presence of lithium bis(trifluoromethanesulfonyl)imide (LiTFSI, 1.0 equiv) produced single-ion conductive organogels (Scheme 3).

The presence of LiTFSI in the cross-linked network changed the material’s rheological behavior. The material made from 0.144 mol L^–1^ solution of dp-poly(**d****-1**) and 0.50 equiv of PDBA still showed gel behavior (*G*′ > *G*″) and self-healing capabilities (Figures S27–S29), akin to its nonlithiated analogue, but was softer with reduced storage and loss moduli (at 1 Hz, *G*′_Li_ = 2227 Pa vs *G*′_non,Li_ = 10233 Pa, *G*″_Li_ = 1099 Pa vs *G*″_non,Li_ = 2923 Pa). Materials made with a 0.287 mol L^–1^ solution of dp-poly(**d****-1**) and 0.50 equiv of PDBA were viscoelastic in the absence of LiTFSI but showed gel-like behavior and self-healing when lithiated (Figures S30–S32). The effect LiTFSI had on the rheological properties of these materials confirms that it is fully incorporated within the cross-linked polymeric network.

The temperature within electrochemical devices is variable during battery operation. Therefore, temperature-dependent rheological measurements were conducted on the lithiated gels, to gain further insight into their mechanical integrity. Both materials retained gel-like behavior across the temperature range studied (25–70 °C). Although the 0.144 mol L^–1^ material softened with increasing temperature (decrease of storage and loss moduli, Figure S33), the 0.287 mol L^–1^ material showed good thermal stability, with only small differences between the storage and loss moduli measured at 25 and 70 °C (*G*′_25 °C_ = 1237 Pa, *G*′_75 °C_ = 1037 Pa, 84% decrease, Figure S34). Retention of properties at elevated temperatures indicated that this latter material was suitable for electrochemical analysis and had potential to be a self-healing, renewable gel polymer electrolyte.

**Figure 8:**
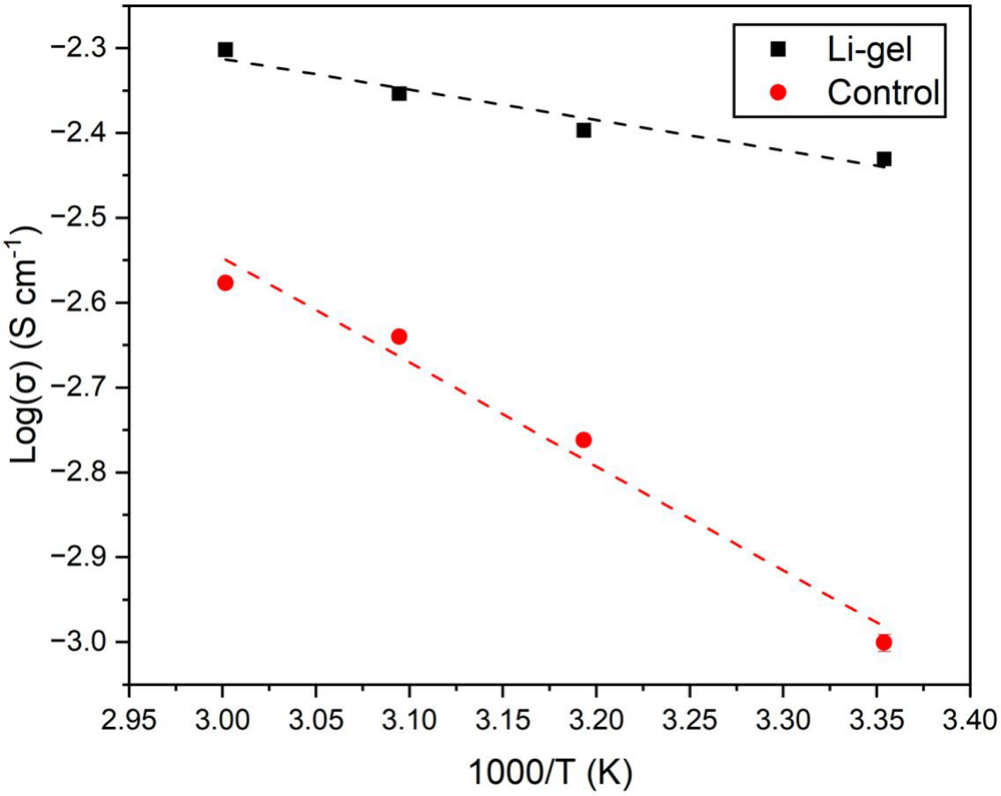
Temperature dependence of ionic conductivity (σ) for lithiated (1 equiv of LiTFSI, 0.144 mol L^–1^; **Li-gel**) and nonlithiated (**control**) organogels made from dp-poly(**d****-1**) (0.287 mol L^–1^ with respect to the repeating unit) with 0.50 equiv of PDBA.

The conductivity of this organogel (referred to as **Li-gel** in the following) was assessed using electrochemical impedance spectroscopy (EIS). Typical Nyquist plots were obtained, with partial semicircles representing bulk resistance (*R*_b_) followed by diagonal lines showing Warburg diffusion (Figure S52). The gel showed a room temperature (25 °C) ionic conductivity of 3.71 × 10^–3^ S cm^–1^. As shown by the Arrhenius plots in Figure 8, conductivity was further enhanced at elevated temperatures as *R*_b_ decreased, reaching 4.99 × 10^–3^ S cm^–1^ at 60 °C (Figure S54, Table S1). As a control, a nonlithiated gel analogue was also analyzed by EIS and showed reduced ionic conductivity across all temperatures compared to the sample containing LiTFSI (Figures S53–S54, Table S2). These initial EIS experiments demonstrate the potential for this system to be used as a gel polymer electrolyte, or GPE. GPEs are attractive electrolyte materials as they alleviate the safety concerns of liquid electrolytes while achieving higher ionic conductivities than solid polymer electrolyte (SPE) alternatives.^31,32^ The ambient ionic conductivity of our system (3.71 × 10^–3^ S cm^–1^ at 25 °C) matches or exceeds recently reported, boron-containing GPEs (see Table S3).^60−63^

In terms of single-ion conducting GPEs featuring anion-trapping boron moieties, the ionic conductivity values reported for **Li-gel** in this study are comparable to those achieved by Ma and co-workers (2.33 × 10^–3^ S cm^–1^ at room temperature)^60^ and Dai et al. (8.4 × 10^–4^ S cm^–1^ at 30 °C).^61^ Similarly, Shi and co-workers have recently reported a novel single-ion conducting boron-centered GPE, which showed ionic conductivity (1.03 × 10^–3^ S cm^–1^ at 32 °C) in the same order of magnitude as the system reported in this study.^62^ Moreover, Xu et al. have reported a self-healing hydrogel polymer electrolyte, based upon copolymers of glycerol monomethacrylate and acrylamide with dynamic borate cross-links, which showed comparable ionic conductivity of 4.5 × 10^–3^ S cm^–1^ at room temperature.^63^

Further electrochemical characterization was carried out. Electrochemical stability was determined by linear sweep voltammetry (Figure S56). **Li-gel** displayed anodic stability up to 4.51 V (vs Li/Li^+^), therefore beyond the 4.2 V stability to oxidation required for practical application. The lithium transference number was also determined using the Bruce–Vincent method, combining DC polarization chronoamperometry and EIS measurements in a symmetrical Li|GPE|Li cell. Although equilibrium was not obtained, **Li-gel** presented high *t*_Li+_ values between 0.88 and 0.92 during combined EIS and chronoamperometry measurements (average of 3 repeats, Table S4 and Figure S57). This data provides an estimate of the *t*_Li+_ of **Li-gel** and is consistent with single-ion conducting behavior, demonstrating the potential of the GPE.

**Figure 9:**
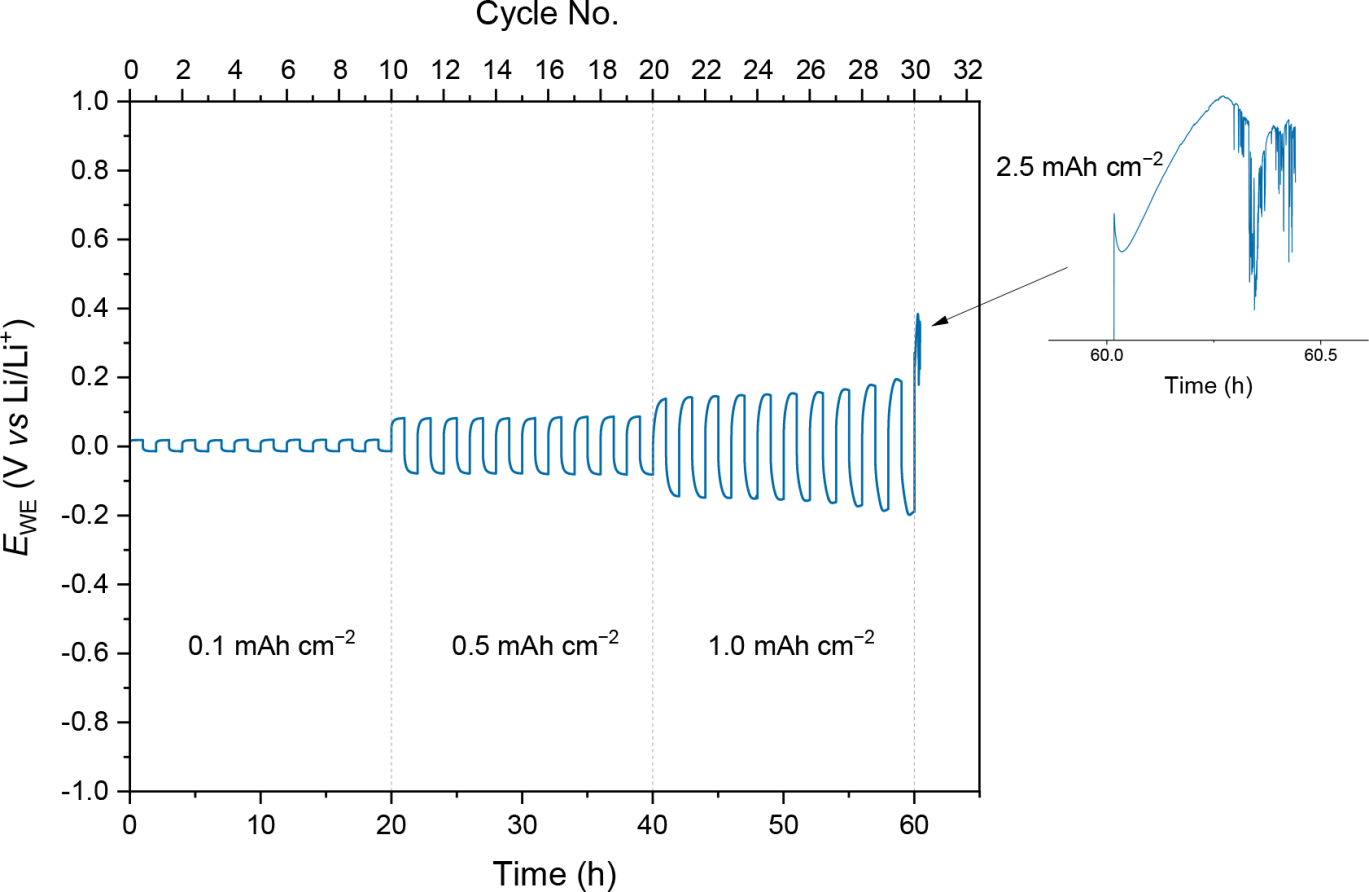
Li^+^ stripping/plating voltage profile of **Li-gel** in a symmetrical Li|**Li-gel**|Li cell at 25 °C. The half-cycle duration was 1 h with a cutoff voltage of ±1.0 V.

Finally, the performance of **Li-gel** for the stripping/plating behavior of Li^+^ was investigated by recording polarization profiles in a symmetrical Li|GPE|Li cell at 25 °C (Figure 9). **Li-gel** exhibited good cycling behavior for at least 10 full cycles (20 h) at consecutive current densities of 0.1, 0.5, and 1.0 mA cm^–2^, with small overpotentials ≤0.05 V (vs Li/Li^+^) for the latter current density. When a large current density of 2.5 mA cm^–2^ was applied, a severe voltage noise was observed which was attributed to short circuits due to lithium dendrites emerging from the electrode surfaces.^64^ Nevertheless, the ability of the material to withstand a critical current density of up to 1.0 mA cm^–2^ is highly promising and compares well with recent single-ion gel polymer electrolytes based on borate pendant groups, even if more studies are needed, including over a longer time frame and in Li|**Li-gel**|cathode cells. However, preliminary results using Li|**Li-gel**|LiFePO_4_ showed poor charging/discharging behavior, suggesting that lithium iron phosphate may not be an appropriate cathode material for this GPE. The dynamic nature of boronic acid cross-linking and the generation of water upon cross-linking may also explain this and the fluctuation in current during the measurement of *t*_Li+._ More investigations are currently underway and will be reported in due course.

Like the majority of polymer electrolytes, the leading boron-containing GPEs rely on fossil fuel derived polymers. Several GPEs have recently been developed from renewable, biobased materials, such as derivatives of cellulose, natural rubber, and chitosan.^65−69^ The electrochemical performance of the sugar-derived system reported here rivals many of these examples and combines the benefits of anion trapping with the sustainability benefits of renewable polymers. Further investigation is therefore warranted to study and optimize the anion-trapping capabilities of PDBA in these systems and its effect on the electrochemical performance of the renewable and self-healing GPE.

## 3 Conclusions

A new self-healing organogel system has been developed from the room-temperature cross-linking of a sugar-derived polyether and 1,4-phenylenediboronic acid (PDBA) in DMSO. The effect of cross-linking density and polymer concentration on the material properties has been demonstrated, with viscoelastic and gel materials favored at polymer concentrations above and below 0.144 mol L^–1^, respectively. As expected, the stiffness of the materials can be tuned according to the equivalents of PDBA: stiffer materials are produced from higher cross-linking densities. Due to the dynamic nature of the boronate ester bonds, the materials show excellent self-healing abilities. After application of up to 100% strain, materials immediately recover at least 74% of their original storage modulus. The boronate ester moieties also allowed lithium ions to be incorporated throughout the cross-linked polymer matrix network, producing single-ion conductive organogels. Gels retain their self-healing nature after lithiation, enabling the production of self-healing, gel polymer electrolytes (GPEs). Initial electrochemical impedance spectroscopy (EIS) measurements show that this system is a promising candidate for Li battery applications. The organogel demonstrates high ambient ionic conductivities of 3.71 × 10^–3^ S cm^–1^, matching or outperforming recently reported GPEs. It also showed high anodic stability (4.51 V vs Li/Li^+^), high transference numbers (0.88–92), and high critical current density (1 mA cm^–2^). This promising electrical performance, coupled with the self-healing properties and renewable nature of the xylose-derived polymer, means there is scope to optimize electrochemical performance (including in whole cell setups) while investigating gelation in common electrolyte solutions, such as ethylene (EC), propylene (PC), or dimethyl carbonate (DMC).

## 4 Experimental Section

### 4.1 Materials and Methods

All reagents were used all purchased, without further purification. d-Xylose and 1,2-*O*-isopropylidene-α,d-xylofuranose were purchased from Carbosynth. Sulfuric acid (H_2_SO_4_) was purchased from VWR. Potassium carbonate (K_2_CO_3_), trifluoroacetic acid (TFA), triethylamine (NEt_3_), and potassium methoxide (KOMe) were purchased from Fischer scientific. Tosylchloride (TsCl), potassium *tert*-butoxide (KO*t*Bu), and 1,4-phenylenediboronic acid (PDBA) were purchased from Merck. 18-Crown-6 was purchased from Acros Organics.

### 4.2 Characterization Methods

#### 4.2.1 NMR Spectroscopy

NMR spectra were recorded in either CDCl_3_, DMSO, or D_2_O on a Bruker-400 or -500 MHz instrument referenced to the corresponding residual solvent as an internal standard: ^1^H NMR spectra (400 or 500 MHz) δH = 7.26, δH = 2.50, and δH 4.79 ppm for residual protiated CDCl_3_, DMSO, and D_2_O, respectively: ^13^C NMR spectra (101 or 126 MHz) δC = 77.16, δC = 39.52 ppm for CDCl_3_, DMSO-*d*_6_, and D_2_O, respectively.

#### 4.2.2 Size-Exclusion Chromatography (SEC)

The number-average molar mass, *M*_n_, and dispersity, *Đ*_M_, of polymer samples were determined by size exclusion chromatography (SEC) in a THF or dimethylacetamide (DMAc) eluent (DMAc contains 0.1% w/v LiBr). Polymer samples were dissolved to a concentration of ∼2 mg/mL. Multianalysis software was used to process the data. THF samples were recorded on an Agilent 1260 Infinity series instrument at 1 mL min^–1^ at 35 °C using two PLgel 5 μm MIXED-D 300 × 7.5 mm columns in series. Samples were detected with a differential refractive index (RI) detector. Number-average molar mass (*M*_n,SEC_) and dispersities (*Đ*_M_ (*M*_w_/*M*_n_)) were calculated against a polystyrene calibration (11 polystyrene standards of narrow molar mass, ranging from *M*_w_ 615 to 568000 Da). DMAc samples were measured on an Agilent 1260 SEC MDS instrument at 0.5 mL min^–1^ at 50 °C using a Polargel-M 300 × 7.5 mm column. Samples were detected using an RI detector. *M*_n,SEC_ and *Đ*_M_ were calculated against a polymethylmethacrylate calibration (11 polymethylmethacrylate standards of narrow molar mass ranging from *M*_w_ 885 to 260900).

#### 4.2.3 Thermogravimetric Analysis (TGA)

TGA was performed on a Setsys Evolution TGA 16/18 from Setaram. The Calisto program was used to collect and process data. Samples were loaded into a 170 μL alumina crucible and heated from 30 to 500 °C at a rate of 10 °C min^–1^.

#### 4.2.4 Differential Scanning Calorimetry (DSC)

Glass transition temperatures, *T*_g_, of polymer samples were found using DSC on a Q20 machine from TA Instruments, controlled by the Q Series program. Samples were loaded into a 10 μL Tzero aluminum pan. Samples were heated and cooled at a rate of 10 °C min^–1^ and immediately submitted to a second heating and cooling cycle at the same rate. The heat flow was recorded against an empty reference 10 μL Tzero aluminum reference pan.

#### 4.2.5 IR Spectroscopy

IR spectra were recorded using a Spectrum 100 FT-IR spectrometer from Perkin-Elmer and were baseline corrected in MatLab.

#### 4.2.6 pH

Measurements were conducted using an MD 8000 L pHenomenal pH meter from VWR.

#### 4.2.7 Rheology

Measurements were conducted using a Discovery HR-2 hybrid rheometer from TA Instruments with a 25 mm parallel plate geometry. Unless otherwise specified, a temperature of 25 °C was maintained throughout the experiments. Oscillatory frequency sweep measurements were conducted at 0.007% strain. Oscillatory strain amplitude measurements were conducted at a frequency of 1 Hz.

#### 4.2.8 Microscopy

Images were taken using a JEOL JSM-7900F field emission scanning electron microscope (FE-SEM) from JEOL U.K., Ltd. Before imaging, samples were coated with 20 nm of gold using a Quorum Q150T S sputter coater from Quorum Tech. Analysis of images was conducted using the Image J software (Java 1.8.0_112 64 bit).

#### 4.2.9 Electrochemistry

Measurements were performed using a modified version of a TCS battery cell (RHD instruments) with blocking stainless steel current collectors (exposed area diameter = 0.8 cm) connected to a Metrohm Autolab PGSTAT204 potentiostat with a FRA32M module. The cell components were dried in a vacuum oven at 70 °C prior to assembly. Temperature control of the cell was achieved using an Autolab Microcell HC temperature-controlled cell stand and temperature controller designed by RHD instruments. The cell was equilibrated for 1 h at each temperature before measurements were taken. Ionic conductivity (σ) was determined by a two-electrode electrochemical impedance spectroscopy (EIS) measurement in the typical frequency range of 0.1 Hz to 0.5 MHz with an applied amplitude of 50 mV in a symmetrical SS|GPE|SS cell. Results shown are averages of five repeat measurements taken at each temperature value. NOVA 2.1 (Metrohm) software was used to analyze the results. The bulk resistance (*R*_b_) was taken as the high-frequency intercept of the *Z*′ axis on the Nyquist plot.^70^ The ionic conductivity values were then calculated using
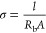
where *l* is the thickness of the gel (determined by triplicate measurements with digital callipers), and *A* is the area of the electrode surface.

Linear sweep voltammetry was used to determine the electrochemical stability in a Li|GPE|SS cell using a lithium counter/reference electrode at 25 °C. The open-cell voltage (OCV) was first determined, and then the voltage swept from the OCV to +6 V with a scan rate of 1 mV s^–1^.

Lithium transference number (*t*_**+**_) was determined using a combined EIS and chronoamperometry method using a Li|GPE|Li symmetrical cell using lithium foil of 0.75 mm thickness and an applied voltage of 10 mV. The measurement was recorded at 25 °C, and *t*_+_ was calculated using the Bruce–Vincent equation
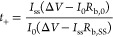
where Δ*V* is the applied voltage and *I*_0_ and *I*_SS_ represent the initial and steady state current before and after DC polarization. *R*_b,0_ and *R*_b,SS_ represent the bulk resistance obtained from EIS measurements before and after DC polarization.

The Li^+^ stripping/plating behavior was measured in symmetrical Li/Li cells (Li thickness = 750 μm) with half-cycle durations of 1 h at 25 °C. Ten full cycles with ±1.0 V cutoff voltages were performed for each current density until the experiment was stopped due to cell failure.

### 4.3 Synthetic Procedures

#### 4.3.1 1,2-*O*-Isopropylidene-xylofuranose (IPXF)

Following an adapted literature procedure:^1^d-xylose (20.00 g, 133.34 mmol, 1.00 equiv), nonanhydrous acetone (500 mL), and 95–98% concentrated H_2_SO_4_ (20 mL, 373.16 mmol, 2.80 equiv) were combined to form a pale-yellow suspension. The suspension was left to stir until the solid had fully dissolved to form a yellow solution (ca. 1 h). Upon full dissolution, an aqueous solution of K_2_CO_3_ (224 mL, 1.10 mol L^–1^, 246.01 mmol, 1.84 equiv) was slowly added. The resultant white suspension was allowed to stir at room temperature with aliquots taken at regular intervals to monitor the deprotection by TLC (1:1 HCCl_3_:acetone eluent) and NMR (*d*_6_-DMSO). After approximately 90 min, near-quantitative formation of the monoprotected sugar was observed. Solid K_2_CO_3_ (17.80 g, 128.79 mmol, 1.04 equiv) was added slowly at room temperature and the pH adjusted until 7–8 by litmus paper. The suspension was then filtered and the acetone removed *in vacuo* at 40 °C. The aqueous phase was washed with DCM (100 mL, ×3), and the organic phases were collected and back extracted with water (50 mL, ×3). The aqueous phases were collected, and the water was removed *in vacuo* at 50 °C. The resultant oil was dissolved in EtOAc and stirred over MgSO_4_ overnight. The suspension was filtered and the solvent removed *in vacuo* at 40 °C to yield 1,2-*O*-isopropylidene-α-xylofuranose (IPXF) as a clear oil (19.76 g, 78%).

#### 4.3.2 1,2-*O*-Isopropylidene-5-*O*-tosyl-xylofuranose (Ts-IPXF)

IPXF (20.00 g, 105.16 mmol, 1.00 equiv) and tosyl chloride (22.06 g, 115.68 mmol, 1.10 equiv) were dissolved in dichloromethane (200 mL). The reaction vessel was cooled over ice, and triethylamine (100 mL, 574.17 mmol, 5.46 equiv) was added. The vessel was left to warm to 20 °C overnight. Ethyl acetate (200 mL) was added, and the mixture was transferred to a separating funnel. The organic phase was then washed with brine (150 mL, ×1), 1 mol L^–1^ sodium hydrogen carbonate (150 mL, ×1), and water (150 mL, ×1). The organic phase was collected and dried over magnesium sulfate, filtered, and concentrated in vacuo at 40 °C to give a white solid. The solid was then stirred over cold Et_2_O (−18 °C, 150 mL) for 10 min, filtered over a glass frit, and rinsed again with cold Et_2_O (50 mL). The precipitate was left to dry in a vacuum oven overnight at 50 °C to give the product as an off-white solid (28.32 g, 82.59 mmol, 79%). Synthesized or commercial IPXF can be used. Quoted yield uses commercial IPXF.

#### 4.3.3 Monomer **d****-1**

To a round-bottom flask was charged Ts-IPXF (15.90 g, 46.04 mmol, 1.00 equiv) and acetonitrile (160 mL). The suspension was agitated at 400 rpm until a clear colorless homogeneous solution was obtained. Potassium methoxide (6.84 g, 96.68 mmol, 2.10 equiv) was added to the solution which was then heated to reflux (80 °C) for 1 h. The resulting dark brown reaction was quenched by addition of water (80 mL). The acetonitrile was removed *in vacuo* at 40 °C. The aqueous phase was extracted with Et_2_O (100 mL, ×3). The organic phases were collected and washed with brine (100 mL, ×1), 1 mol L^–1^ sodium hydrogen carbonate (100 mL, ×1), and water (100 mL, ×1). The organic phases were collected, dried over MgSO_4_, filtered, and concentrated *in vacuo* at 40 °C to give **d**-**1** as a clear oil. For further purification, the oil was stirred over CaH_2_ at 80 °C overnight and vacuum distilled (1 × 10^–2^ mbar, 40 °C) to yield the oxetane as a clear oil (5.52 g, 32.11 mmol, 70%).

#### 4.3.4 General Procedure for the Polymerization of **d**-**1**

Under an argon atmosphere, a centrifuge tube was charged with the oxetane monomer (2.00 g, 11.63 mmol, 100.00 equiv), KO^t^Bu (232 μL, 0.5 mol L^–1^ in THF, 1.00 equiv), and 18-crown-6 (232 μL, 0.5 mol L^–1^ in THF, 1.00 equiv). The tube was sealed and heated to 120 °C with stirring. After 22 h, the vial was cooled, and the solid was dissolved in the minimum amount of CHCl_3_ and then precipitated from cold Et_2_O. The suspension was centrifuged (3500 rpm, 5 min), and the solid phase was collected. The polymer was then redissolved in CHCl_3_ and precipitated twice more from cold Et_2_O with centrifugation (3500 rpm, 5 min). The solid was collected and dried in a vacuum oven for 24 h at 100 °C to yield the polyether (1.59 g, 9.26 mmol, 80%).

#### 4.3.5 General Procedure for the Deprotection of Polyethers

Following an adapted literature procedure: to a dram vial was added poly(**d**-**1**) (1.86 g, 10.83 mmol, *M*_n,SEC_ 7250–12,600 g mol^–1^, *Đ*_M_ = 1.2) and DCM (7.40 mL). Upon dissolution, the reaction mixture was cooled to 0 °C, and a 4:1 TFA:H_2_O solution (18.00 mL) was added. After 8 h, the produce was precipitated from cold Et_2_O, and the resulting suspension was centrifuged (3500 rpm, 5 min). The solid phase was collected and rinsed twice more in cold Et_2_O or until the supernatant was neutral by a litmus test. The solid phase was collected, but not dried, to yield the polyether deprotected at 91% (calculated by relative integration of protected and deprotected anomeric environments in ^1^H NMR spectroscopy, see Section 4.2.5). The product was immediately dissolved into an appropriate solvent for analysis or further reaction (1.40 g, 10.60 mmol, 98%, *M*_n,SEC_ 20,300–24,300 g mol^–1^, *Đ*_M_ = 1.21–1.71).

#### 4.3.6 General Procedure for the Cross-Linking of dp-poly(**d**-**1**) and PDBA

In a dram vial, PDBA (0.05–1.00 equiv with respect to the deprotected polymer repeat unit; an example calculation of the amount of PDBA used in the synthesis can be found in the Supporting Information) was added to a solution of dp-poly(**d**-**1**) in DMSO (0.072–0.409 mol L^–1^). The mixture was stirred at room temperature until gelation occurred (as confirmed by a vial inversion test). For rheological and self-healing measurements, the mixture was transferred into a glass Petri dish (diameter = 3.5 cm, depth = approximately 0.8 cm) before the point of gelation. The mixture was covered and allowed to mature overnight at room temperature.

#### 4.3.7 General Procedure for the Formation of Conductive Organogels

In a dram vial, PDBA (0.50 equiv with respect to the deprotected polymer repeat unit) and lithium bis(trifluoromethanesulfonyl)imide (LiTFSI, 1.00 equiv) were added to a solution of dp-poly(**d**-**1**) in DMSO (0.144 or 0.287 mol L^–1^). The mixture was stirred at room temperature and then was transferred into a glass Petri dish (diameter = 3.5 cm, depth = approximately 0.8 cm) before the point of gelation. The mixture was covered and allowed to mature at room temperature. To perform EIS, a disc of diameter 1.1 cm was cut from the gel after 3–4 h of curing. The gel was then transferred into the electrochemical cell for analysis: the disc of gel was placed onto a stainless-steel current collector (diameter of exposed area = 0.8 cm) electrode inside a TSC battery cell (RHD Instruments). The second electrode was placed on top of the gel, and the cell was closed and tightened to ensure sufficient electrode contact. For LSV and DC polarization measurements where lithium electrodes (*d* = 1.0 cm) were used, the electrodes were first polished with a nylon brush before contact with the gel was made.

## Supporting Information Available

Cross-linking procedures, NMR spectra, photographs of vial inversion tests, FT-IR spectra, detailed rheological characterizations, FE-SEM microscopy, TGA and DSC traces, and additional electrochemistry results (PDF)

## ABBREVIATIONS

PDBA: 1,4-phenylenediboronic acid
FT-IR: Fourier-transform infrared
TGA: thermal gravimetric analysis
DSC: differential scanning calorimetry
LiTFSI: lithium bis(trifluoromethanesulfonyl)imide
GPE: polymer gel electrolyte
PVA: poly(vinyl alcohol)
DMSO: dimethyl sulfoxide
DMF: dimethylformamide
PVAc: poly(vinyl acetate)
PEG: poly(ethyleneglycol)
SPE: solid polymer electrolyte
ROP: ring-opening polymerization
DMAc: dimethylacetamide
LiBr: lithium bromide
EIS: electrochemical impedance spectroscopy
DC: direct current
SEC: size exclusion chromatography
THF: tetrahydrofuran
