# Supplementary material for: Cross-Linking of Sugar-Derived Polyethers and Boronic Acids for Renewable, Self-Healing, and Single-Ion Conducting Organogel Polymer Electrolytes
Source: ACS Appl Energy Mater. 2023 Feb 22;6(5):2924–35. doi: 10.1021/acsaem.2c03937 (PMC10015429; doi:10.1021/acsaem.2c03937)
Supplement: Supplementary file 1 — ae2c03937_si_001.pdf [file ae2c03937_si_001.pdf]

Supporting Information

Cross-linking of Sugar-Derived Polyethers and Boronic Acids for Renewable, Self-healing and Single-Ion Conducting Organogel Polymer Electrolytes

Emma L. Daniels,[a,b,c] James R. Runge,[a,b] Matthew Oshinowo,[a,b] Hannah S. Leese*[a,c] and Antoine Buchard*[a,b]

[a]University of Bath Institute for Sustainability, Claverton Down, Bath, BA2 7AY

[b]Department of Chemistry, University of Bath, Claverton Down, Bath, BA2 7AY

[c]Materials for Health Lab, Department of Chemical Engineering, University of Bath, Claverton Down, Bath, BA2 7AY

Email: a.buchard@bath.ac.uk; h.s.leese@bath.ac.uk

SUPPORTING INFORMATION

1. General Procedures S4
1.1 Example calculation of PDBA equiv S4
1.2 Cross-linking and monosubstitution of PDBA S4
2. Characterisation of Compounds S5
2.1 1,2-O-Isopropylidene-xylofuranose (IPXF) S5
2.2 1,2-O-isopropylidene-5-O-tosyl-xylofuranose (Ts-IPXF) S6
2.3 Monomer (**D-1**) S7
2.4 Polyether, poly(**D-1**) S8
2.5 Deprotected Polyether, dp-poly(**D-1**) S9
3. Vial Inversion Tests S10
3.1 0.287 mol L^{-1} dp-poly(**D-1**), 0.00 – 1.00 PDBA equiv. S10
3.2 0.144 mol L^{-1} dp-poly(**D-1**), 0.125 – 1.00 PDBA equiv. S10
3.3 0.409 mol L^{-1} dp-poly(**D-1**), 0.05 – 0.125 PDBA equiv. S11
3.4 0.072 mol L^{-1} dp-poly(**D-1**), 0.25 – 1.00 PDBA equiv. S11
3.5 pH Stability S12
4. FT-IR Spectroscopy S13
4.1 Deprotected polyether, dp-poly(**D-1**) S13
4.2 1,4-phenyl diboronic acid (PDBA) S13
4.3 0.287 mol L^{-1} dp-poly(**D-1**), 0.125 – 1.00 PDBA equiv. S14
5. Rheology S15
5.1 Frequency sweep of 0.287 mol L^{-1} dp-poly(**D-1**), 0.25 PDBA equiv S15
5.2 Frequency sweep of 0.287 mol L^{-1} dp-poly(**D-1**), 0.50 PDBA equiv S15
5.3 Frequency sweep of 0.287 mol L–1 dp-poly(**D-1**), 1.00 PDBA equiv. S16
5.5 Frequency sweep of 0.144 mol L–1 dp-poly(**D-1**), 0.50 PDBA equiv. S17
5.6 Frequency sweep of 0.144 mol L^{-1} dp-poly(**D-1**), 1.00 PDBA equiv S17
5.7 Frequency sweep of 0.409 mol L^{-1} dp-poly(**D-1**), 1.00 PDBA equiv S18
5.8 Strain sweep of 0.144 mol L^{-1} dp-poly(**D-1**), 0.50 PDBA equiv. S18
5.9 Frequency sweep of 0.144 mol L^{-1} dp-poly(**D-1**), 0.50 PDBA equiv. with LiTFSI S19
5.10 Strain ramp of 0.144 mol L^{-1} dp-poly(**D-1**), 0.50 PDBA equiv. with LiTFSI S19
5.11 Step strain of 0.144 mol L^{-1} dp-poly(**D-1**), 0.50 PDBA equiv. with LiTFSI S20
5.12 Frequency sweep of 0.287 mol L^{-1} dp-poly(**D-1**), 0.50 PDBA equiv. with LiTFSI S20
5.13 Strain ramp of 0.287 mol L^{-1} dp-poly(**D-1**), 0.50 PDBA equiv. with LiTFSI S21
5.14 Step strain of 0.287 mol L^{-1} dp-poly(**D-1**), 0.50 PDBA equiv. with LiTFSI S21
5.15 Temperature ramp of 0.144 mol L^{-1} dp-poly(**D-1**), 0.50 PDBA equiv. with LiTFSI S22
5.16 Temperature ramp of 0.287 mol L^{-1} dp-poly(**D-1**), 0.50 PDBA equiv. with LiTFSI S22

6. FE-FEM S23
6.1 Cross section of 0.144 mol L^{-1} dp-poly(**D-1**), 0.25 PDBA equiv. S23
6.2 Cross section of 0.144 mol L^{-1} dp-poly(**D-1**), 0.50 PDBA equiv. S23
6.3 Side view of 0.144 mol L^{-1} dp-poly(**D-1**), 0.50 PDBA equiv S24
6.4 Cross section of 0.144 mol L^{-1} dp-poly(**D-1**), 1.00 PDBA equiv. S24
7. Thermal Characterisation S25
7.1 TGA Traces S25
7.1.1 Polyether, poly(**D-1**) S25
7.1.2 Deprotected polyether, dp-poly(**D-1**) S25
7.1.3 0.144 mol L^{-1} dp-poly(**D-1**), 0.25 PDBA equiv. S26
7.1.4 0.144 mol L^{-1} dp-poly(**D-1**), 0.50 PDBA equiv. S26
7.1.5 0.144 mol L^{-1} dp-poly(**D-1**), 1.00 PDBA equiv. S27
7.1.6 0.144 mol L^{-1} dp-poly(**D-1**), 0.25 PDBA equiv. nonlyophilized S27
7.1.7 0.144 mol L^{-1} dp-poly(**D-1**), 0.50 PDBA equiv. nonlyophilized S28
7.1.8 0.144 mol L^{-1} dp-poly(**D-1**), 1.00 PDBA equiv. nonlyophilized S28
7.2 DSC Traces S29
7.2.1 Polyether, poly-(**D-1**) S29
7.2.2 Deprotected Polyether, dp-poly(**D-1**) S29
7.2.3 0.144 mol L^{-1} dp-poly(**D-1**), 0.25 PDBA equiv. S30
7.2.4 0.144 mol L^{-1} dp-poly(**D-1**), 0.50 PDBA equiv. S30
7.2.5 0.144 mol L^{-1} dp-poly(**D-1**), 1.00 PDBA equiv. S31
8. Electrochemistry Impedance Spectroscopy (EIS) S32
8.1 Nyquist plots of 0.287 mol L^{-1} dp-poly(**D-1**), 0.50 PDBA equiv. with LiTFSI S32
8.2 Nyquist plots of 0.287 mol L^{-1} dp-poly(D-1), 0.50 PDBA equiv. without LiTFSI S33
8.3 Temperature dependent conductivity of 0.287 mol L^{-1} dp-poly(D-1), 0.50 PDBA equiv. with LiTSFI S34
8.4 Temperature dependent conductivity of 0.287 mol L^{-1} dp-poly(D-1), 0.50 PDBA equiv. without LiTSFI S34
8.5 Comparison with literature ionic conductivity values S35
8.6 Linear Sweep Voltammetry S36
8.7 Lithium Transference Number, t_{Li}+ S36
9. References S37

1. General Procedures

1.1 Example calculation of PDBA equiv.

Example calculation of amount of PDBA, assuming 38 mg of a 91% deprotected polymer was used with 1.00 equiv. of PDBA;

$M_{r,protected}$ = 172 g mol^{-1}
$M_{r,deprotected}$ = 132 g mol^{-1}
$M_{r.PDBA}$ = 165.75 g mol^{-1}

$M_{r,polymer\ average}$ = (0.09 × 172) + (0.91 × 172) = 148.39 g mol^{-1}

$n_{polymer}$ = 38 ÷ 148.39 = 0.256 mmol = n_{PDBA}

m_{PDBA} = 0.256 × 165.75 = 42.4 mg

Unless otherwise stated, quantities deprotection was assumed.

1.2 Cross-linking and monosubstitution of PDBA

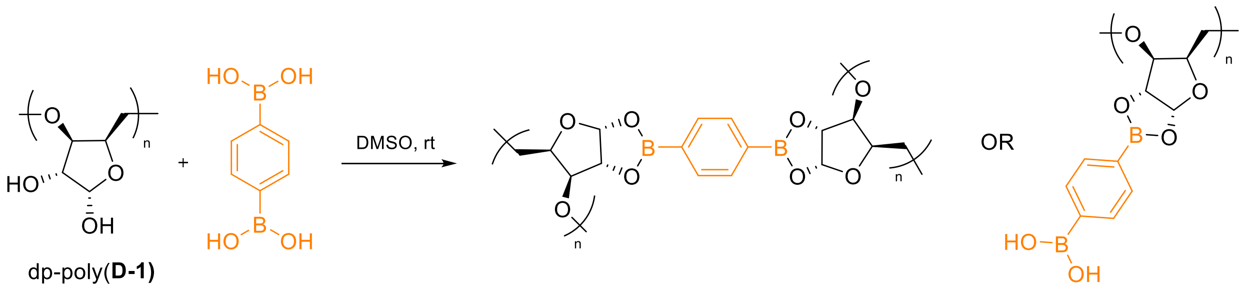

Scheme S1 Reaction of dp-poly(D-1) and 1,4-phenylenediboronic acid (PDBA) can afford cross-linked or monosubstituted products, or those containing a combination of both.

2. Characterisation of Compounds

2.1 1,2-O-Isopropylidene-xylofuranose (IPXF)

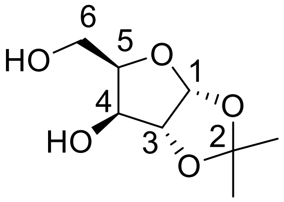

IPXF: Clear, colourless oil, 78% yield (19.76 g)

^{1}H NMR (400 MHz, $CDCl_3$, δ in ppm): 5.90 (s, 1H, H-1), 4.45 (d, J = 2.6 Hz, 1H, H-3), 4.23 (dd, J = 5.6, 2.6 Hz, 1H, H-4), 4.17 – 3.87 (m, 5H, H-5/H-6), 1.42 (s, 3H, CH_3), 1.25 (s, 3H, CH_3).

^{13}C NMR (101 MHz, $CDCl_3$, δ in ppm): 111.8 (C-2), 104.9 (C-1), 85.7 (C-3), 79.2 (C-5), 76.5 (C-4), 60.9 (C-6), 26.8 (CH_3), 26.2 (CH_3).

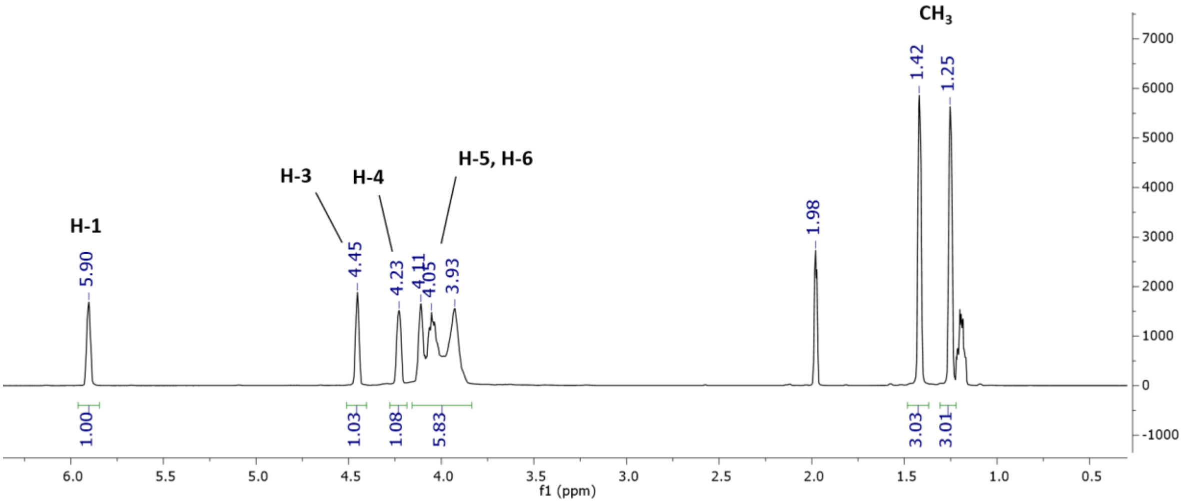

Figure S1 Annotated ^{1}H NMR spectrum of IPXF in chloroform-d.

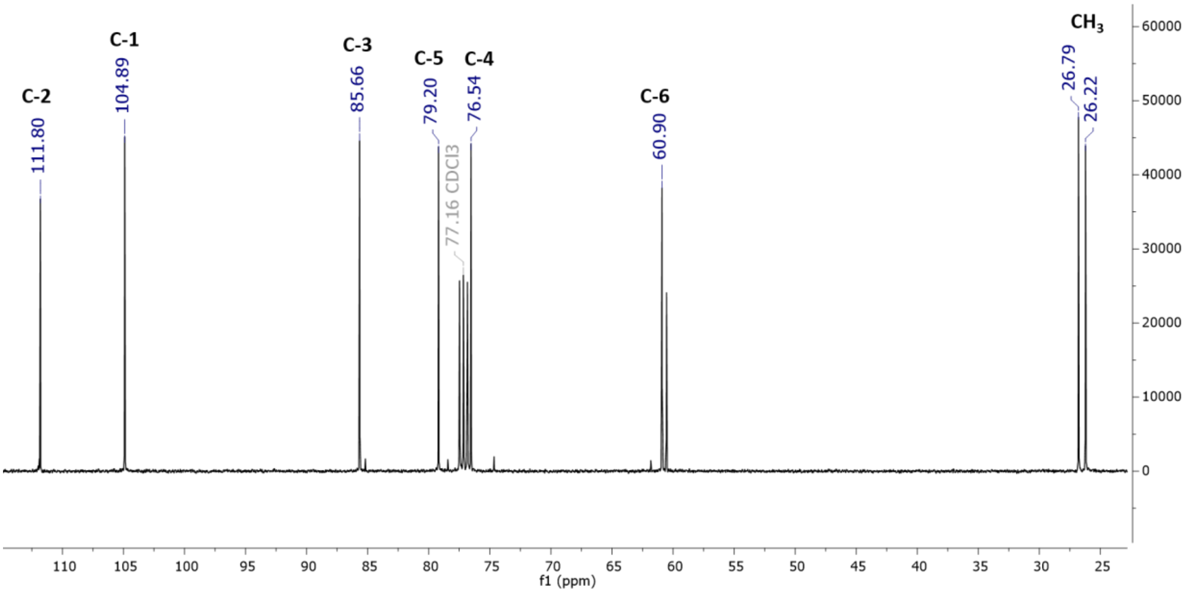

Figure S2 Annotated ^{13}C{^{1}H} NMR spectrum of IPXF in chloroform-d.

2.2 1,2-O-isopropylidene-5-O-tosyl-xylofuranose (Ts-IPXF)

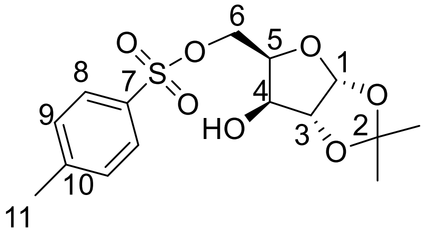

Ts-IPXF: Off-white solid, 79% yield, 28.32 g

^{1}H NMR (400 MHz, $CDCl_3$, δ in ppm): 7.83 (d, J = 8.8 Hz, 2H, H-8), 7.38 (d, J = 8.8 Hz, 2H, H-9), 5.90 (d, J = 3.6 Hz, 1H, H-1), 4.53 (d, J = 3.6 Hz, 1H, H-3), 4.41 – 4.31 (m, 4H, H-4/H-5/H-6), 4.11 – 4.15 (m, 1H, H-6'), 2.48 (s, 3H, H-11), 1.49 (s, 3H, CH_3), 1.35 – 1.30 (m, 3H, CH_3).

^{13}C NMR (101 MHz, $CDCl_3$, δ in ppm): 145.3 (C-7), 132.4 (C-10), 130.0 (C-8), 128.0 (C-9), 112.1 (C-2), 104.9 (C-1), 85.1 (C-3), 77.6 (C-5), 74.3 (C-4), 66.1 (C-6), 26.8 (CH_3), 26.2 (CH_3), 21.2 (C-11).

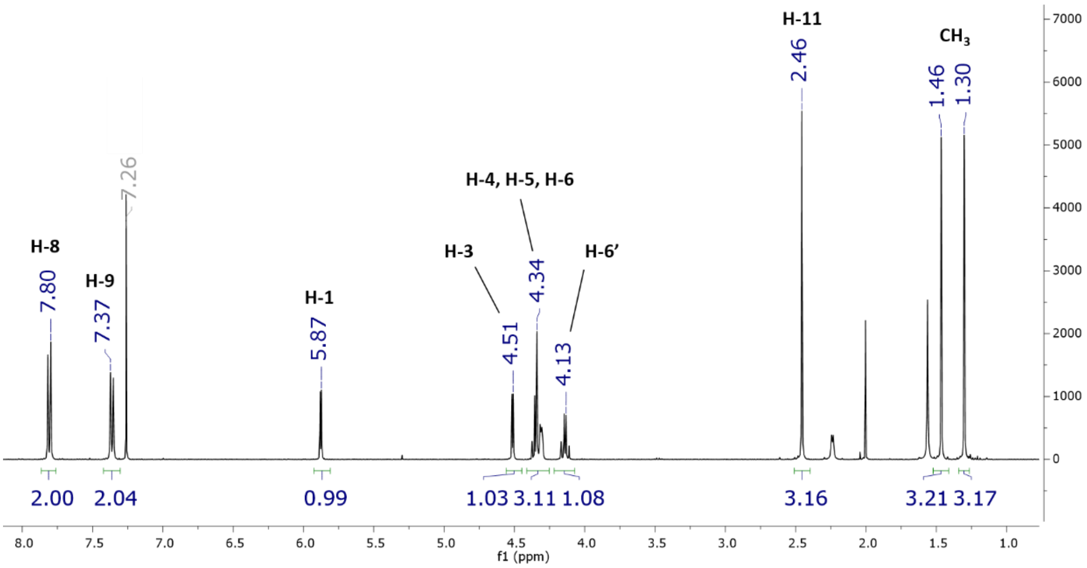

Figure S3 Annotated ^{1}H NMR spectrum of Ts-IPXF in chloroform-d.

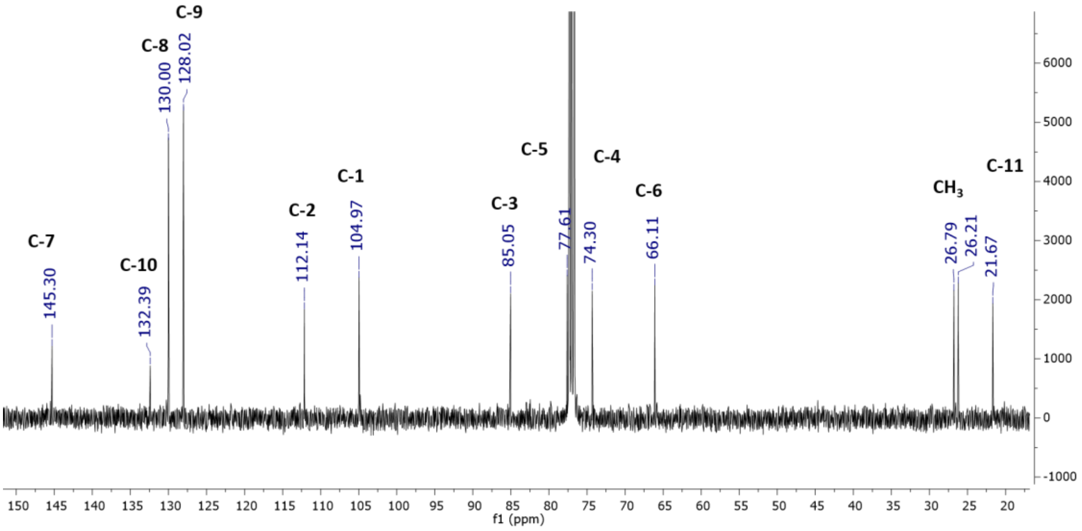

Figure S4 Annotated ^{13}C{^{1}H} NMR spectrum of Ts-IPXF in chloroform-d.

2.3 Monomer (D-1)

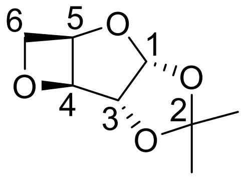

D-1: Clear, colourless oil, 70% yield, 5.52 g

^{1}H NMR (400 MHz, $CDCl_3$, δ in ppm): 6.18 (d, J = 3.7 Hz, 1H, H-1)), 5.10 (d, J = 4.0, 1H, H-4), 5.02 (dt, J = 4.2, 2.3 Hz, 1H, H-5), 4.67 – 4.57 (m, 2H, H-3/H-6), 4.15 (dd, J = 7.7, 2.3 Hz, 1H, H-6), 1.32 (s, 3H, CH_3), 1.28 (s, 3H, CH_3).

^{13}C NMR (100 MHz, $CDCl_3$, δ in ppm): 113.7 (C-2), 108.1 (C-1), 87.4 (C-4), 84.5 (C-3), 78.3 (C-5/C-6), 27.8 (CH_3), 27.1 (CH_3).

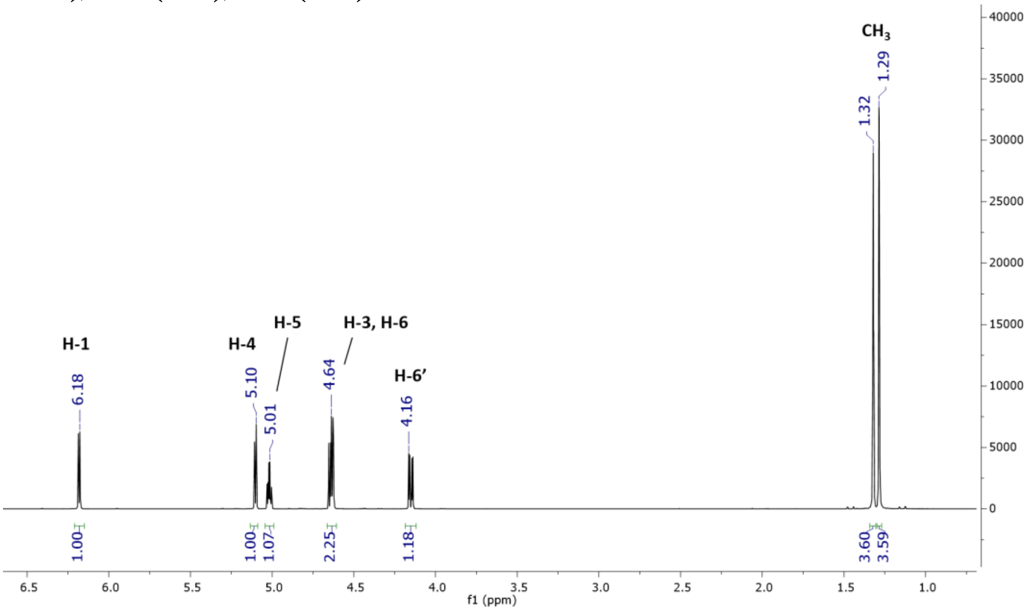

Figure S5 Annotated ^{1}H NMR spectrum of D-1 in chloroform-d.

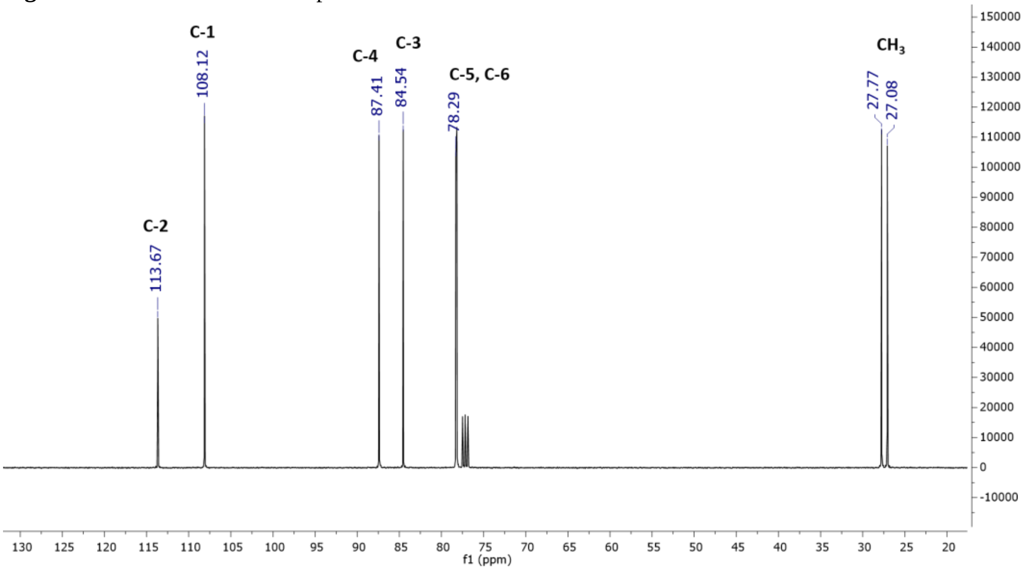

Figure S6 Annotated ^{13}C{^{1}H} NMR spectrum of D-1 in chloroform-d.

2.4 Polyether, poly(D-1)

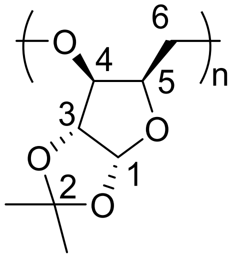

Polyether: Beige solid, 80% yield, 1.59 g

^{1}H NMR (400 MHz, $CDCl_3$, δ in ppm): 5.88 (d, J = 3.8 Hz, 1H, H-1), 4.60 (d, J = 3.7 Hz, 1H, H-3), 4.33 – 4.27 (m, 1H, H-5), 3.91 (m, J = 5.9, 4.0 Hz, 2H, H-4/H-6), 3.60 (dd, J = 10.3, 6.7 Hz, 1H, H-6), 1.48 (s, 3H, CH_3), 1.31 (s, 3H, CH_3).

^{13}C NMR (101 MHz, $CDCl_3$, δ in ppm): 111.7 (C-2), 105.3 (C-1), 83.1 (C-4), 81.9 (C-3), 79.5 (C-5), 68.0 (C-6), 26.4 (CH_3), 27.1 (CH_3).

$\boldsymbol{M}_{\mathbf{n,SEC}}$ = 12,500 mol^{-1}, $Đ_M$ = 1.23, T_g = 138 °C, T_m = 281 °C, T_c = 246 °C, $T_{d,max}$ = 372 °C, $T_{d,5\%}$ = 361°C

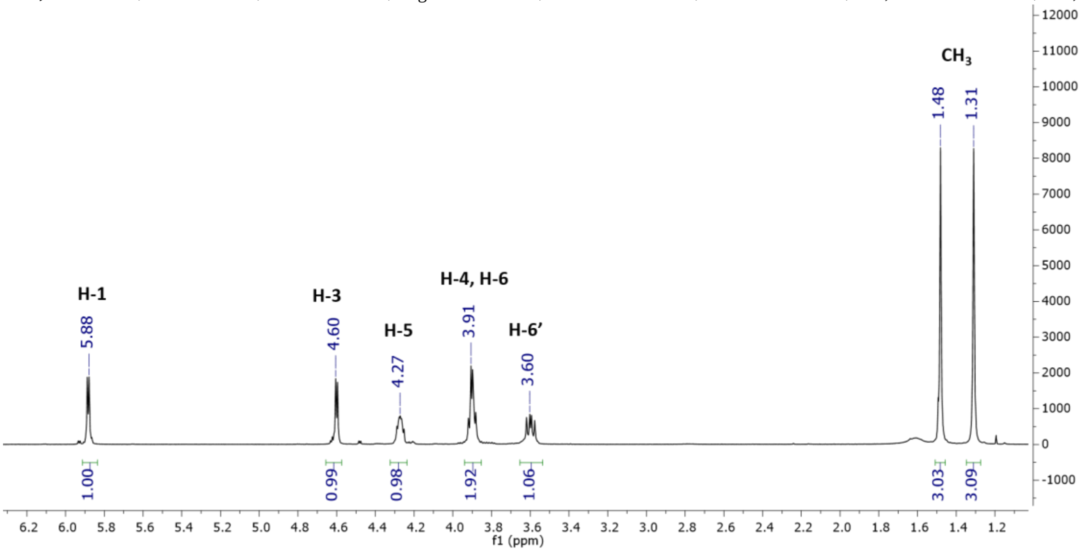

Figure S7 Annotated ^{1}H NMR spectrum of poly(**D-1**) in chloroform-d.

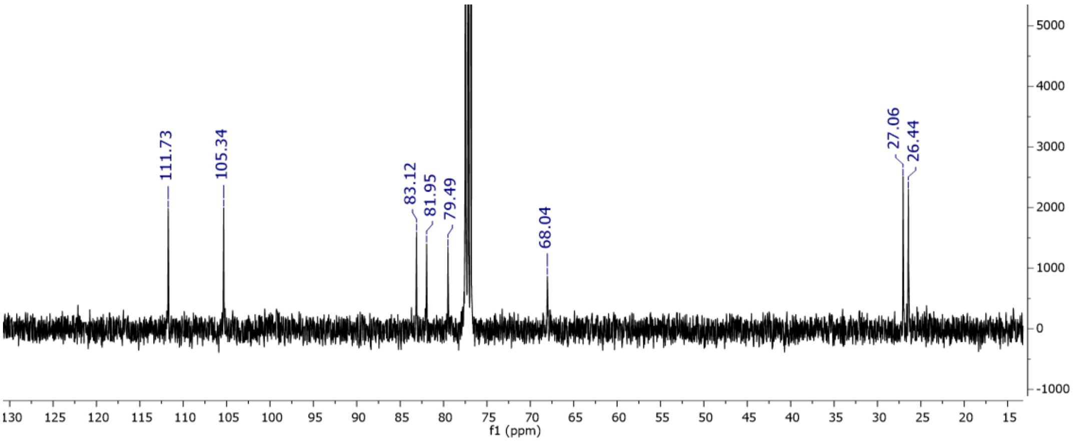

Figure S8 Annotated $^{13}C\{^{1}H\}$ NMR spectrum of poly(**D-1**) in chloroform-d.

2.5 Deprotected Polyether, dp-poly(D-1)

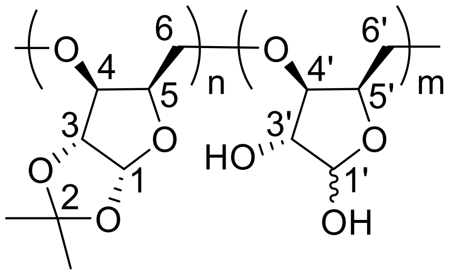

Deprotected Polyether: Off-white solid, 91% deprotected (H-1 : [H-1'α + H-1'β] = 0.09:0.91)

^{1}H NMR (500 MHz, DMSO-d$_6$, δ in ppm): 5.86 – 5.72 (m, H-1), 5.11 (s, H-1'β), 4.87 (s, H-1'α), 4.57 (s, br, H-3), 4.19 – 4.02 (m, J = 23.4 Hz, H-5/H-5'), 3.91 – 3.61 (m, H-3'/H-4/H-4'/H-6/H-6'), 3.56 – 3.45 (m, H-6), 3.41 – 3.36 (m, H-6'), 1.36 (s, CH_3), 1.21 (s, CH_3).

^{13}C NMR (126 MHz, DMSO-d$_6$, δ in ppm): 103.3 (C-1'α), 96.5 (C-1'β), 84.8 (C-3'). 79.2 (C-5'), 76.8/74.7 (C-4'), 69.7 (C-6'), 27.1 (CH_3), 26.5 (CH_3).

$\boldsymbol{M}_{\mathbf{n,SEC}}$ = 17,400 g mol^{-1}, $Đ_M$ = 1.22, T_g = not observed, T_m = not observed, T_c = not observed, $T_{d,max}$ = 272 °C, $T_{d,5\%}$ = 136 °C

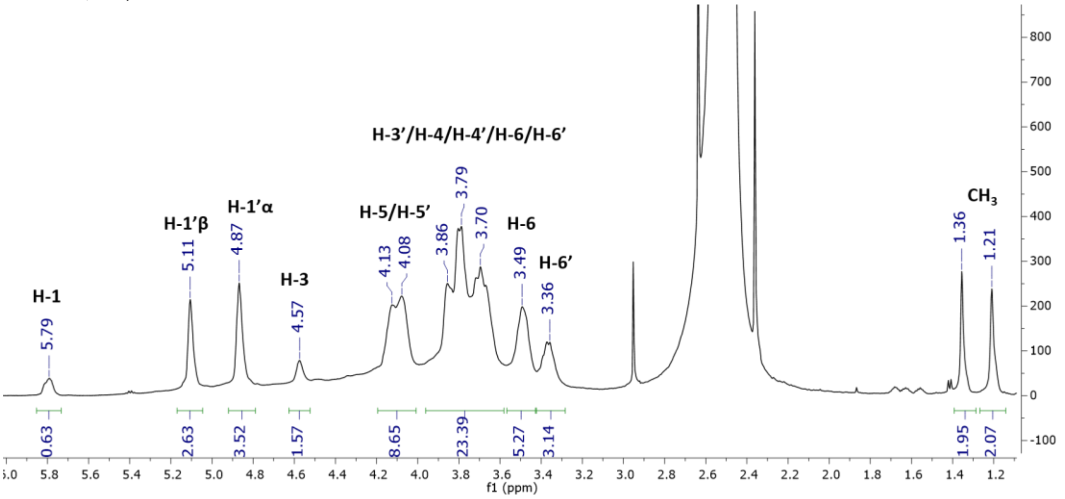

Figure S9 Annotated ^{1}H NMR spectrum of dp-poly(**D-1**) in chloroform-d. A 91% deprotected sample has been chosen to demonstrate the protected vs. deprotected proton environments.

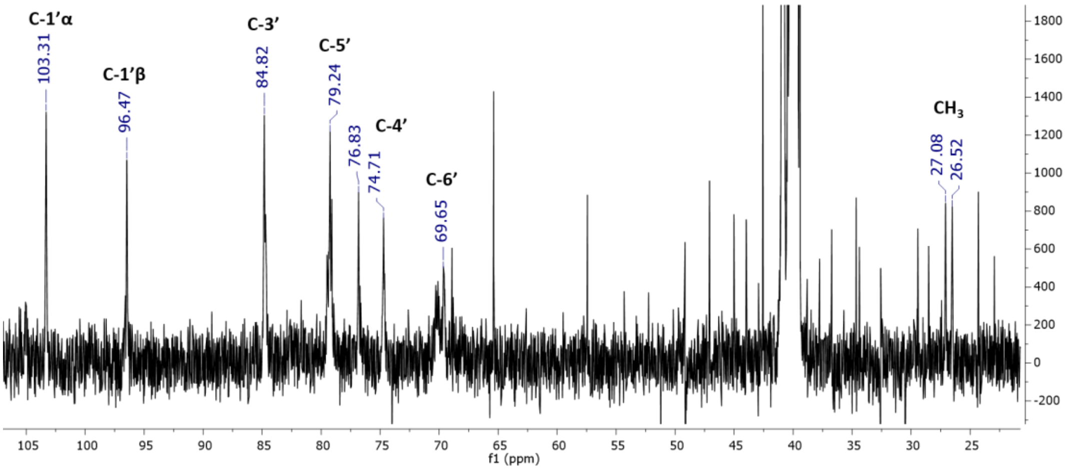

Figure S10 Annotated ^{13}C{^{1}H} NMR spectrum of dp-poly(**D-1**) in chloroform-d.

3. Vial Inversion Tests

3.1 0.287 mol L^{-1} dp-poly(D-1), 0.00 – 1.00 PDBA equiv.

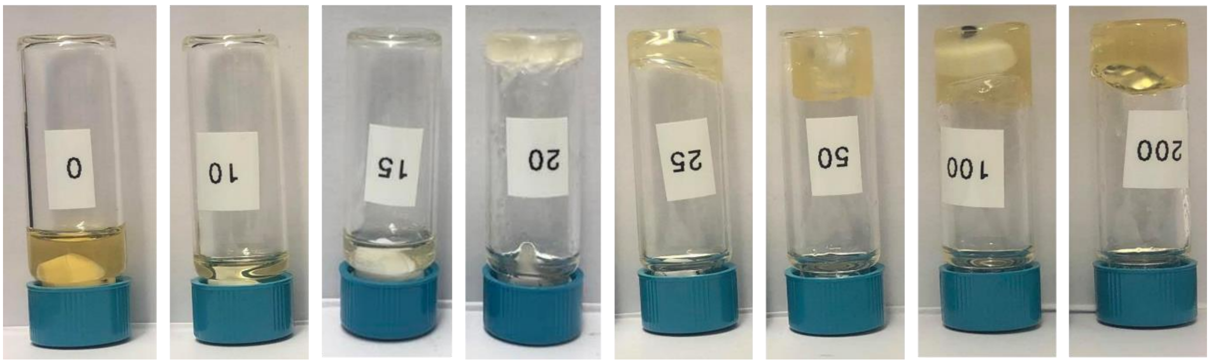

Figure S11 Vial inversion tests of reactions between a 0.287 mol L^{-1} dp-poly(**D-1**) solution in DMSO with increasing equiv. of PDBA. Equiv. of PDBA from left to right: 0.00, 0.05, 0.075, 0.10, 0.125, 0.25, 0.50, and 1.00. Vial labels show the theoretical maximum percentage cross-linking, *i.e* the percentage of polymer hydroxy groups that would be functionalised if all added PDBA reacted.

3.2 0.144 mol L^{-1} dp-poly(D-1), 0.125 – 1.00 PDBA equiv.

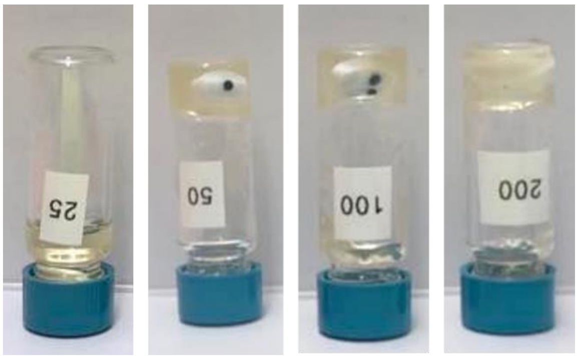

Figure S12 Vial inversion tests of reactions between a 0.144 mol L^{-1} dp-poly(**D-1**) solution in DMSO with increasing equiv. of PDBA. Equiv. of PDBA from left to right: 0.125, 0.25, 0.50, and 1.00. Vial labels show the theoretical maximum percentage cross-linking, *i.e* the percentage of polymer hydroxy groups that would be functionalised if all added PDBA reacted.

3.3 0.409 mol L^{-1} dp-poly(D-1), 0.05 – 0.125 PDBA equiv.

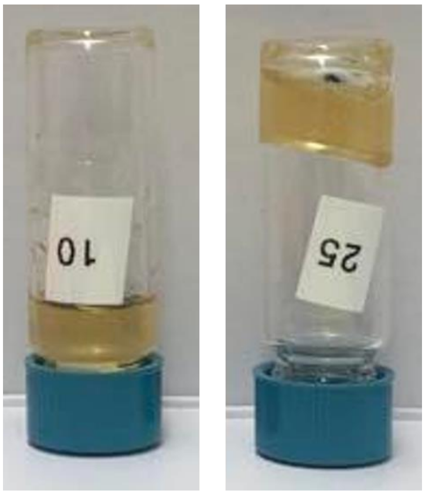

Figure S13 Vial inversion tests of reactions between a 0.409 mol L^{-1} dp-poly(**D-1**) solution in DMSO with increasing equiv. of PDBA. Equiv. of PDBA from left to right: 0.05, and 0.125. Vial labels show the theoretical maximum percentage cross-linking, *i.e* the percentage of polymer hydroxy groups that would be functionalised if all added PDBA reacted.

3.4 0.072 mol L^{-1} dp-poly(D-1), 0.25 – 1.00 PDBA equiv.

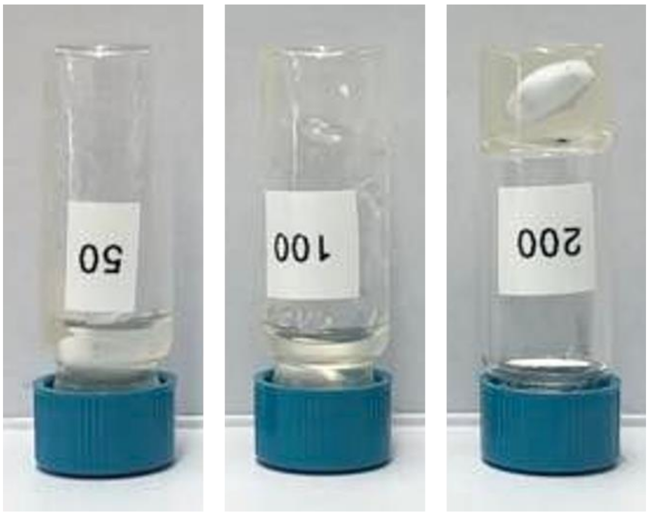

Figure S14 Vial inversion tests of reactions between a 0.072 mol L^{-1} dp-poly(**D-1**) solution in DMSO with increasing equiv. of PDBA. Equiv. of PDBA from left to right: 0.25, 0.50, and 1.00. Vial labels show the theoretical maximum percentage cross-linking, *i.e* the percentage of polymer hydroxyl groups that would be functionalised if all added PDBA reacted.

3.5 pH Stability

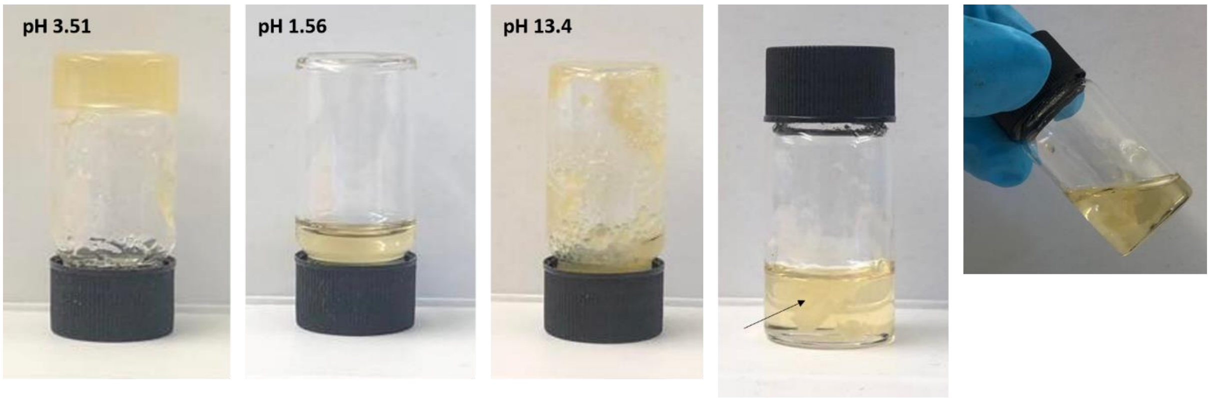

Figure S15 Vial inversion tests of organogels made with a 0.144 mol L^{-1} dp-poly(**D-1**) solution in DMSO with 0.5 equiv. of PDBA upon addition of small amount of acid or base. From left to right: original organogel; organogel after addition of 1 mol L^{-1} HCl; organogel after addition of 1 mol L^{-1} NaOH; partial reformation of gel after addition of 1 mol L^{-1} HCl to basic gel mixture.

4. FT-IR Spectroscopy

4.1 Deprotected polyether, dp-poly(D-1)

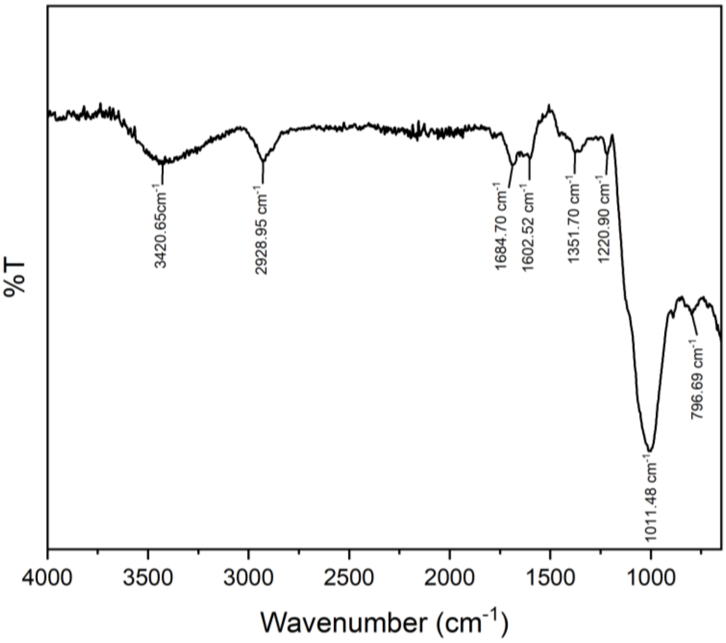

Figure S16 FT-IR spectrum of dp-poly(**D-1**). Key wavenumbers are highlighted.

4.2 1,4-phenyl diboronic acid (PDBA)

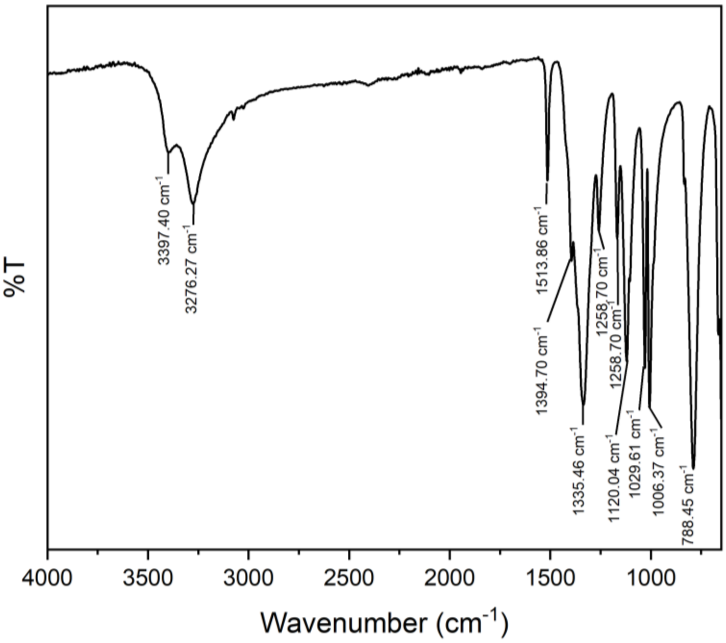

Figure S17 FT-IR spectrum of PDBA. Key wavenumbers are highlighted.

4.3 0.287 mol L^{-1} dp-poly(D-1), 0.125 – 1.00 PDBA equiv.

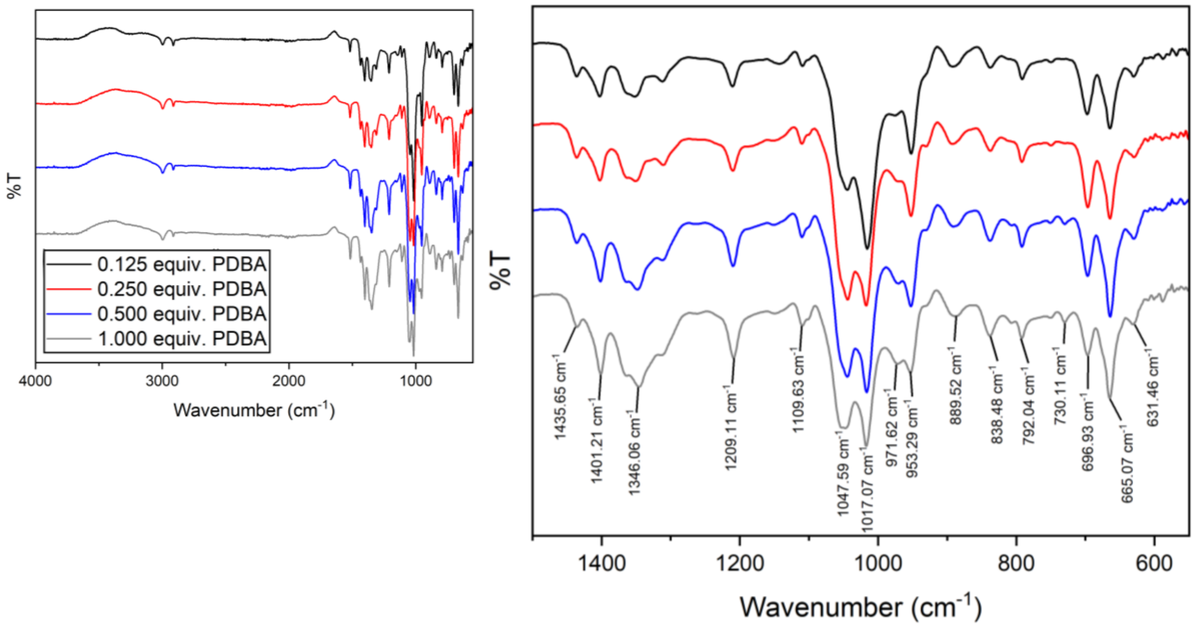

Figure S18 FT-IR spectrum of organogels made from dp-poly(**D-1**) and PDBA with a polymer concentration of 0.287 mol L^{-1} and 0.125 to 1.00 equiv. of PDBA. Key wavenumbers are highlighted.

5. Rheology

5.1 Frequency sweep of 0.287 mol L^{-1} dp-poly(D-1), 0.25 PDBA equiv.

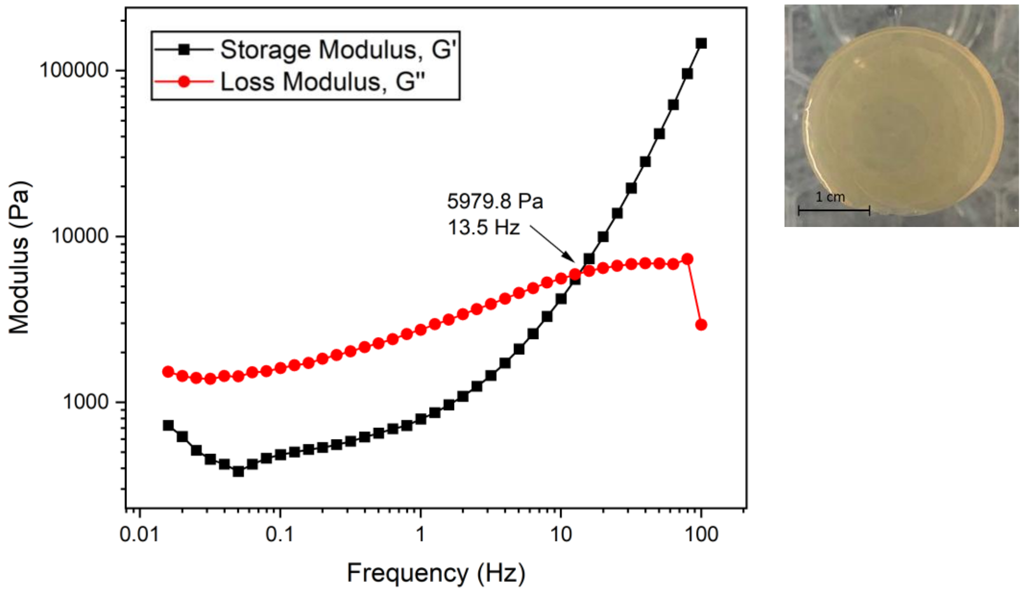

Figure S19 LEFT: Dynamic frequency sweep of organogel made from dp-poly(**D-1**) and PDBA with a polymer concentration of 0.287 mol L^{-1} and 0.25 equiv. of PDBA. Annotations show the crossover frequency and modulus. RIGHT: Photograph of the organogel used in rheological studies.

5.2 Frequency sweep of 0.287 mol L^{-1} dp-poly(D-1), 0.50 PDBA equiv.

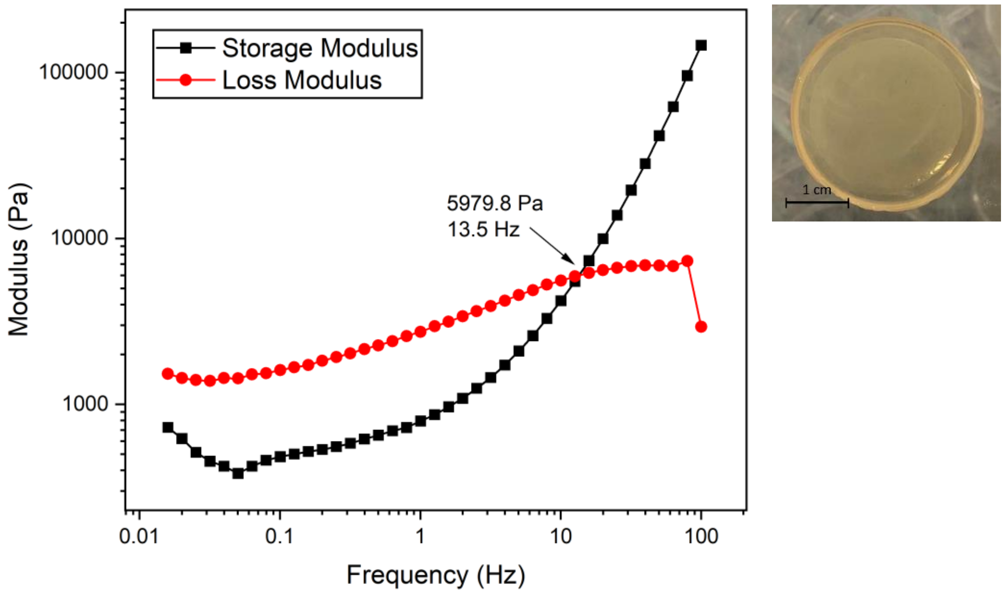

Figure S20 LEFT: Dynamic frequency sweep of organogel made from dp-poly(**D-1**) and PDBA with a polymer concentration of 0.287 mol L^{-1} and 0.50 equiv. of PDBA. Annotations show the crossover frequency and modulus. RIGHT: Photograph of the organogel used in rheological studies.

5.3 Frequency sweep of 0.287 mol L–1 dp-poly(D-1), 1.00 PDBA equiv.

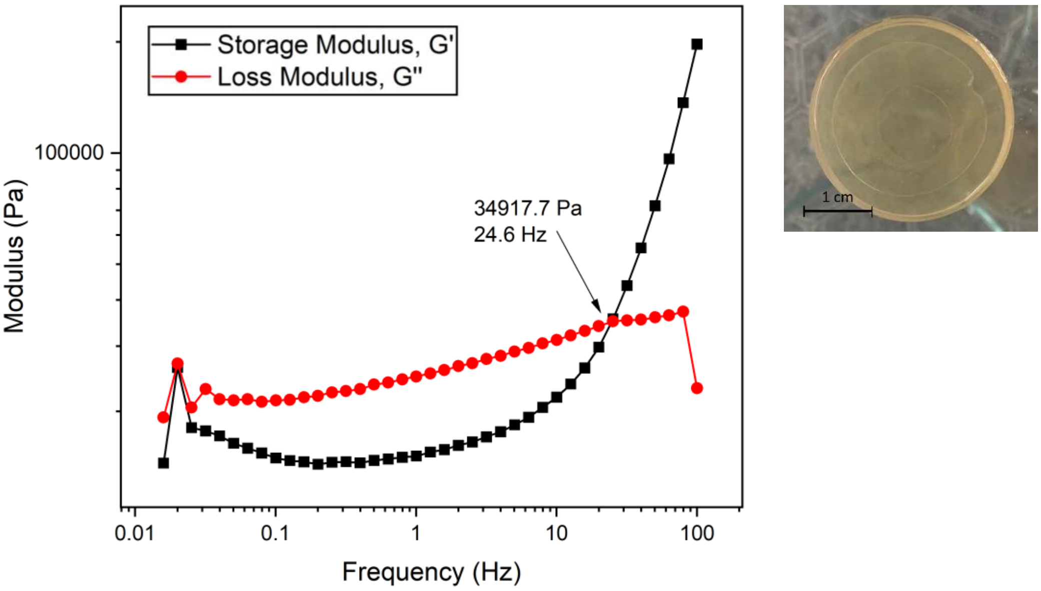

Figure S21 LEFT: Dynamic frequency sweep of organogel made from dp-poly(D-**1**) and PDBA with a polymer concentration of 0.287 mol L^{-1} and 1.00 equiv. of PDBA. Annotations show the crossover frequency and modulus. RIGHT: Photograph of the organogel used in rheological studies.

5.4 Frequency sweep of 0.144 mol L–1 dp-poly(d-1), 0.25 PDBA equiv.

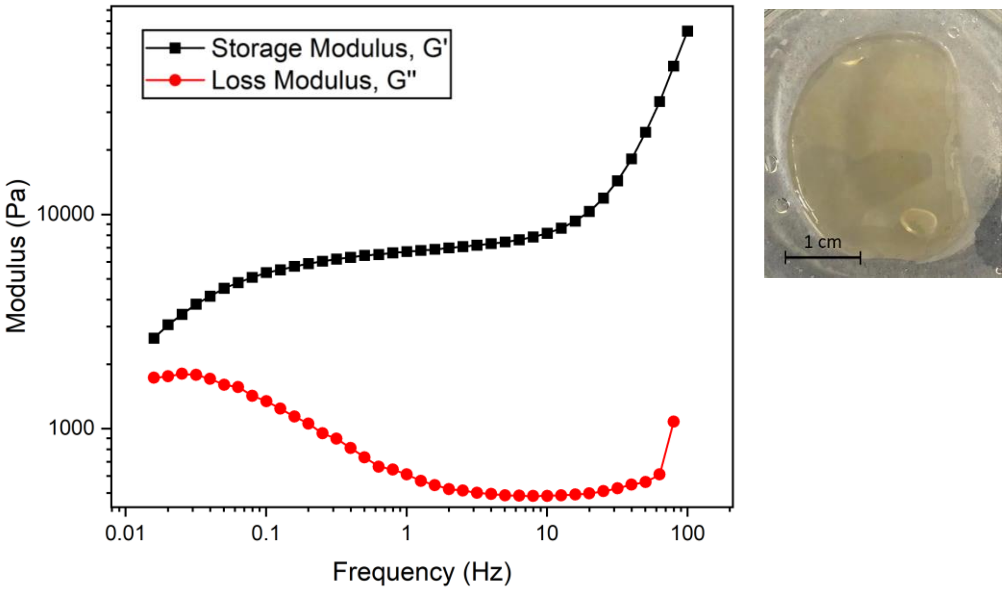

Figure S22 LEFT: Dynamic frequency sweep of organogel made from dp-poly(D-**1**) and PDBA with a polymer concentration of 0.144 mol L^{-1} and 0.25 equiv. of PDBA. RIGHT: Photograph of the organogel used in rheological studies.

5.5 Frequency sweep of 0.144 mol L–1 dp-poly(D-1), 0.50 PDBA equiv.

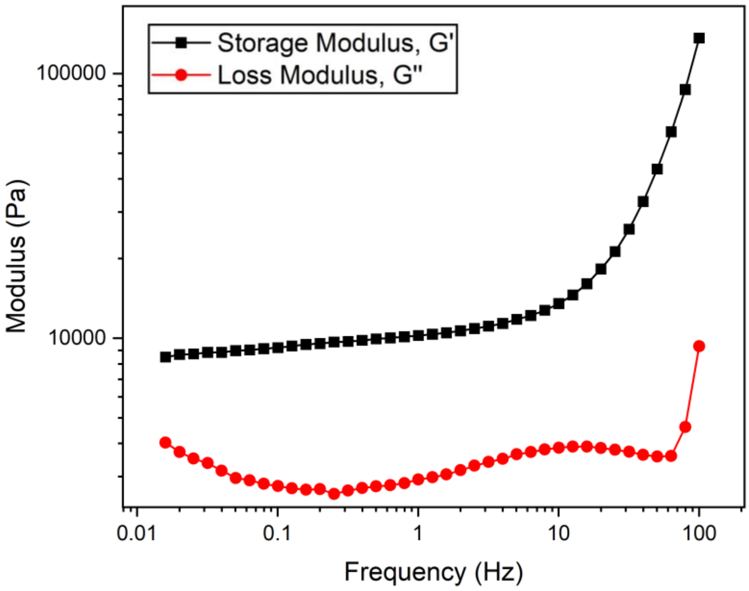

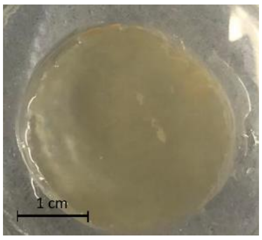

Figure S23 LEFT: Dynamic frequency sweep of organogel made from dp-poly(**D-1**) and PDBA with a polymer concentration of 0.144 mol L^{-1} and 0.50 equiv. of PDBA. RIGHT: Photograph of the organogel used in rheological studies.

5.6 Frequency sweep of 0.144 mol L^{-1} dp-poly(D-1), 1.00 PDBA equiv.

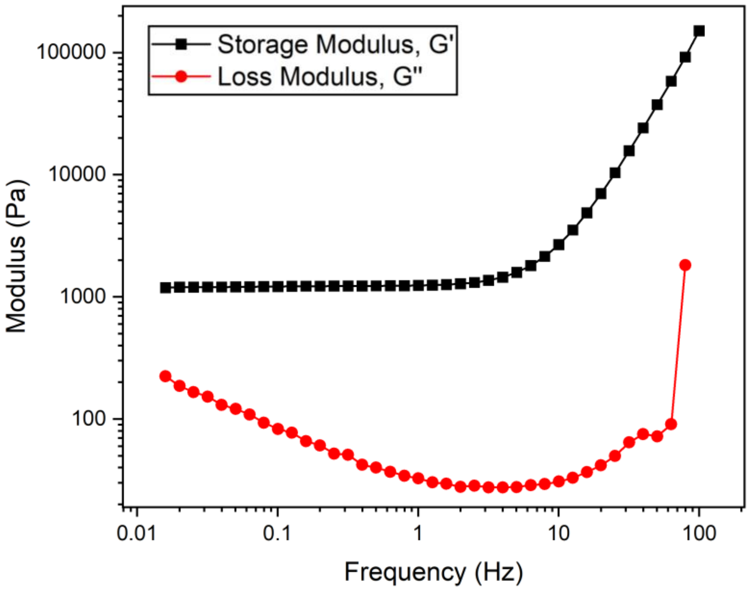

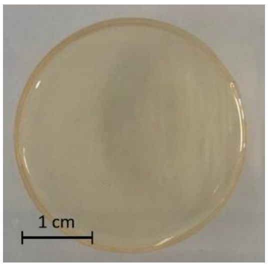

Figure S24 LEFT: Dynamic frequency sweep of organogel made from dp-poly(**D-1**) and PDBA with a polymer concentration of 0.144 mol L^{-1} and 1.00 equiv. of PDBA. RIGHT: Photograph of the organogel used in rheological studies.

5.7 Frequency sweep of 0.409 mol L^{-1} dp-poly(D-1), 1.00 PDBA equiv.

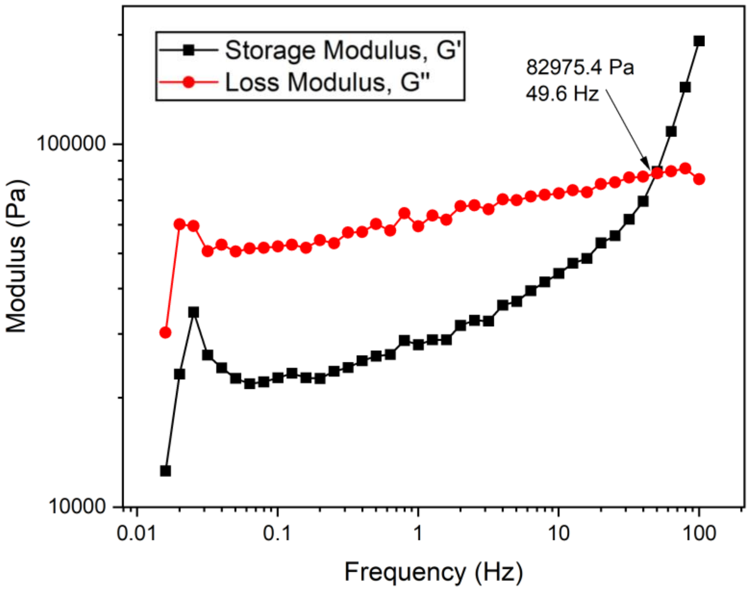

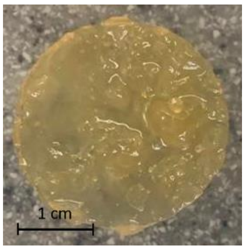

Figure S25 LEFT: Dynamic frequency sweep of organogel made from dp-poly(**D-1**) and PDBA with a polymer concentration of 0.409 mol L^{-1} and 1.00 equiv. of PDBA. Annotations show the crossover frequency and modulus. RIGHT: Photograph of the organogel used in rheological studies.

5.8 Strain sweep of 0.144 mol L^{-1} dp-poly(D-1), 0.50 PDBA equiv.

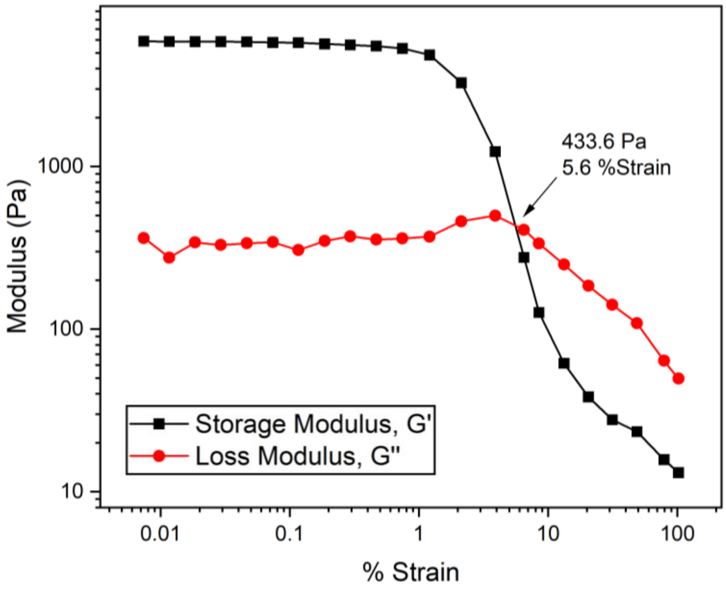

Figure S26 Dynamic strain sweep of organogel made from dp-poly(**D-1**) and PDBA with a polymer concentration of 0.144 mol L^{-1} and 0.50 equiv. of PDBA conducted at 1 Hz. Annotations show crossover modulus and critical % strain.

5.9 Frequency sweep of 0.144 mol L^{-1} dp-poly(D-1), 0.50 PDBA equiv. with LiTFSI

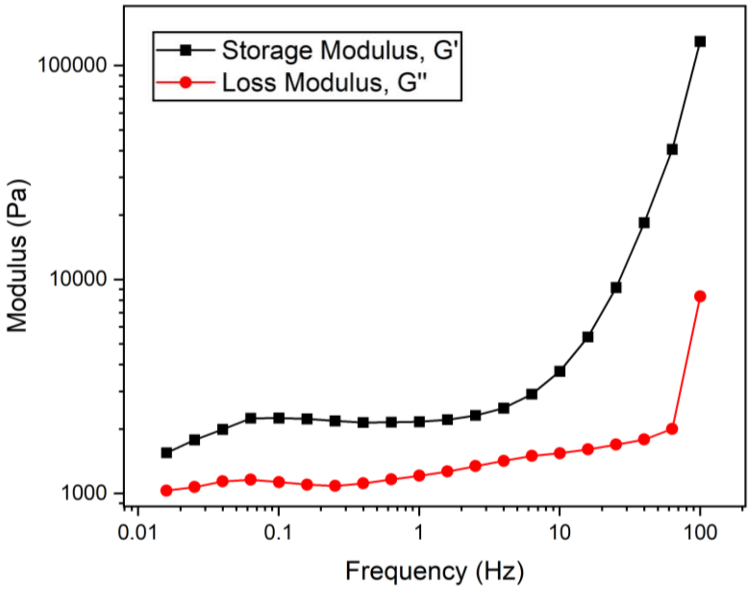

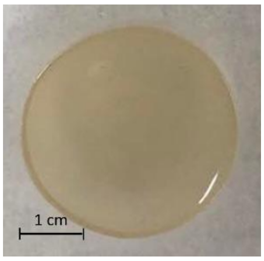

Figure S27 LEFT: Dynamic frequency sweep of lithiated organogel made from dp-poly(**D-1**), PDBA, and LiTFSI (0.144 mol L^{-1}) with a polymer concentration of 0.144 mol L^{-1} and 0.50 equiv. of PDBA. RIGHT: Photograph of the lithiated organogel used in rheological studies.

5.10 Strain ramp of 0.144 mol L^{-1} dp-poly(D-1), 0.50 PDBA equiv. with LiTFSI

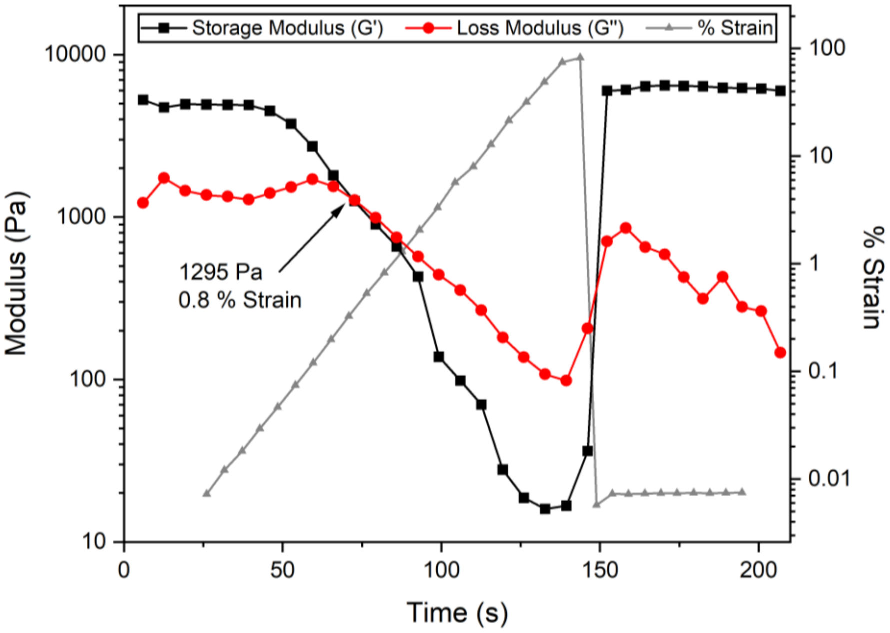

Figure S28 Change in moduli during a strain ramp, measured at a frequency of 1 Hz, of a lithiated organogel made from dp-poly(**D-1**), PDBA, and LiTFSI (0.144 mol L^{-1}) with a polymer concentration of 0.144 mol L^{-1} and 0.50 equiv. of PDBA. The percentage strain was increased from 0.007 % to 100 %, then strain was released and the moduli were measured at 0.007 % strain. Annotation shows the point of moduli crossover.

5.11 Step strain of 0.144 mol L^{-1} dp-poly(D-1), 0.50 PDBA equiv. with LiTFSI

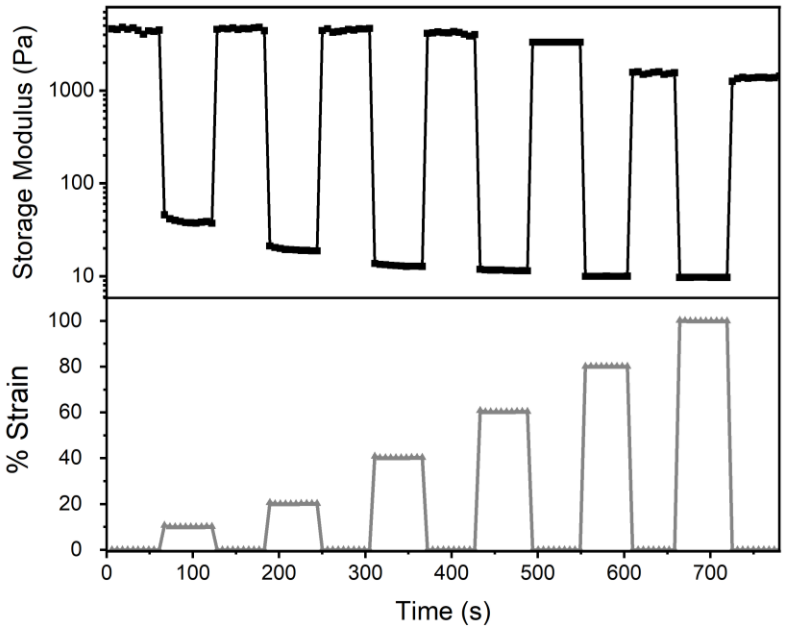

Figure S29 Gel fracture and self-healing of lithiated organogel made from dp-poly(**D-1**), PDBA, and LiTFSI (0.144 mol L^{-1}) with a polymer concentration of 0.287 mol L^{-1} and 0.50 equiv. of PDBA in sequential strain steps. Top shows the measured storage moduli at each strain step, as described in the bottom plot (0% → 10% → 0% → 20% → 0% → 40% → 0% → 60% → 0% → 80% → 0% → 100% → 0%, measured at 1 Hz frequency).

5.12 Frequency sweep of 0.287 mol L^{-1} dp-poly(D-1), 0.50 PDBA equiv. with LiTFSI

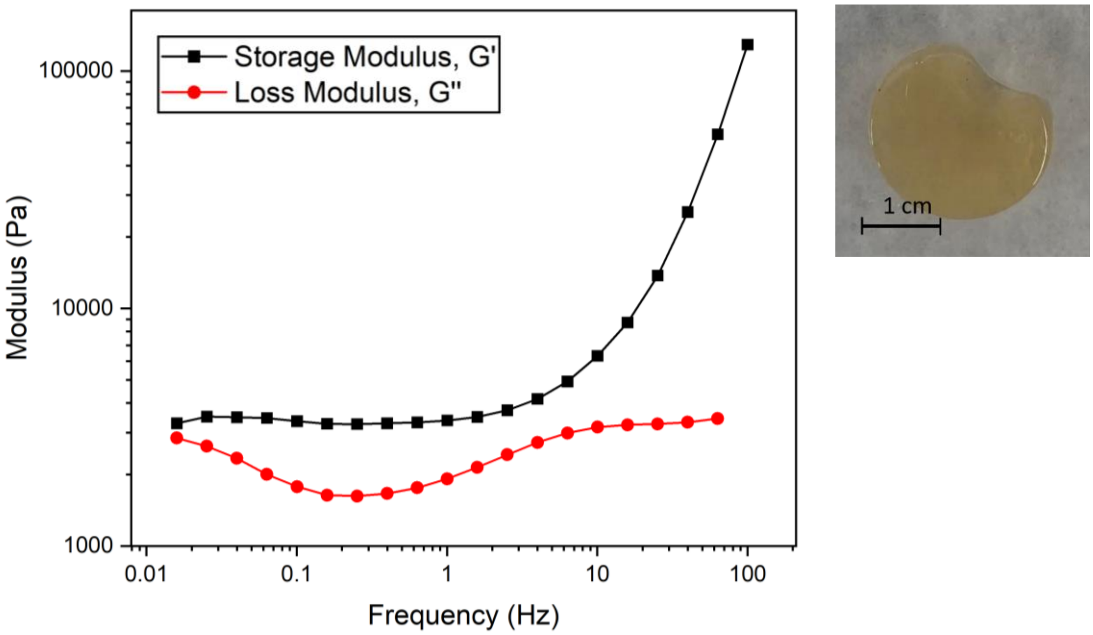

Figure S30 LEFT: Dynamic frequency sweep of lithiated organogel made from dp-poly(**D-1**), PDBA, and LiTFSI (0.144 mol L^{-1}) with a polymer concentration of 0.287 mol L^{-1} and 0.50 equiv. of PDBA. RIGHT: Photograph of the lithiated organogel used in rheological studies.

5.13 Strain ramp of 0.287 mol L^{-1} dp-poly(D-1), 0.50 PDBA equiv. with LiTFSI

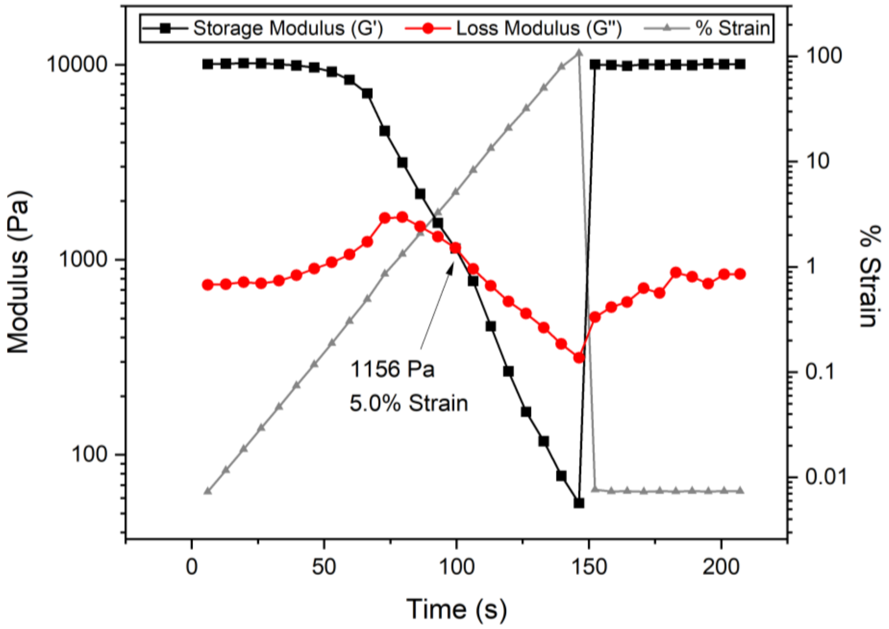

Figure S31 Change in moduli during a strain ramp, measured at a frequency of 1 Hz, of a lithiated organogel made from dp-poly(**D-1**), PDBA, and LiTFSI (0.144 mol L^{-1}) with a polymer concentration of 0.287 mol L^{-1} and 0.50 equiv. of PDBA. The percentage strain was increased from 0.007 % to 100 %, then strain was released and the moduli were measured at 0.007 % strain. Annotation shows the point of moduli crossover.

5.14 Step strain of 0.287 mol L^{-1} dp-poly(D-1), 0.50 PDBA equiv. with LiTFSI

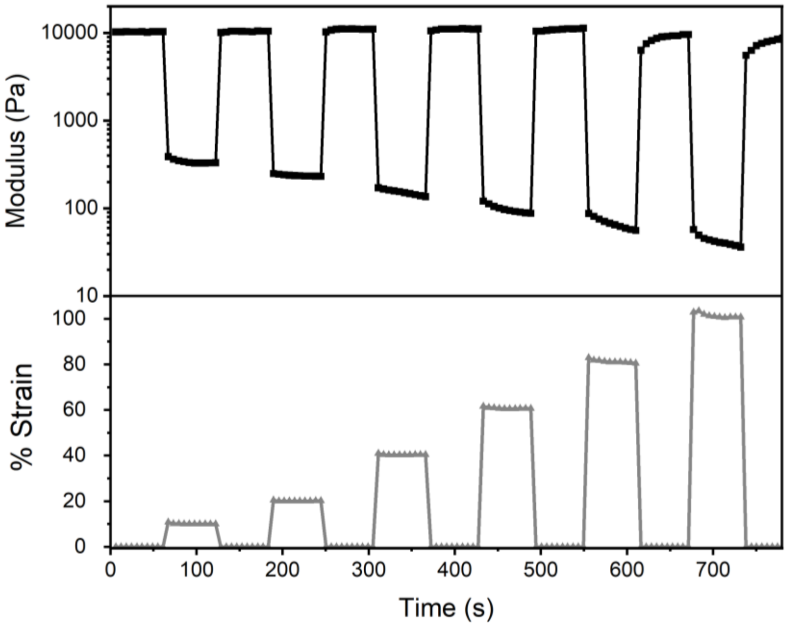

Figure S32 Gel fracture and self-healing of lithiated organogel made from dp-poly(**D-1**), PDBA, and LiTFSI (0.144 mol L^{-1}) with a polymer concentration of 0.287 mol L^{-1} and 0.50 equiv. of PDBA in sequential strain steps. Top shows the measured storage moduli at each strain step, as described in the bottom plot (0% → 10% → 0% → 20% → 0% → 40% → 0% → 60% → 0% → 80% → 0% → 100% → 0%, measured at 1 Hz frequency).

5.15 Temperature ramp of 0.144 mol L^{-1} dp-poly(D-1), 0.50 PDBA equiv. with LiTFSI

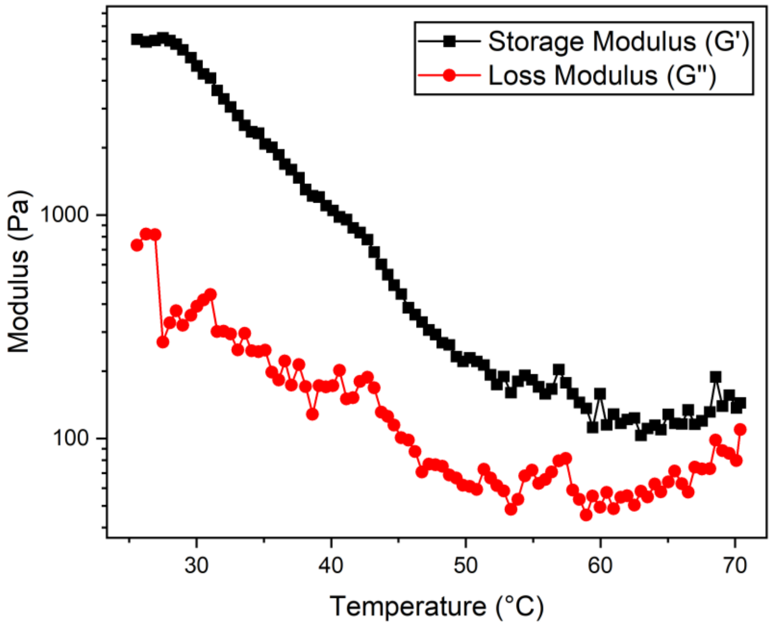

Figure S33 Dynamic temperature ramp (25 – 70 °C) of lithiated organogel made from dp-poly(**D-1**), PDBA, and LiTFSI (0.144 mol L^{-1}) with a polymer concentration of 0.144 mol L^{-1} and 0.50 equiv. of PDBA. Storage and loss moduli were recorded at a constant frequency of 1 Hz and a strain of 0.007 %.

5.16 Temperature ramp of 0.287 mol L^{-1} dp-poly(D-1), 0.50 PDBA equiv. with LiTFSI

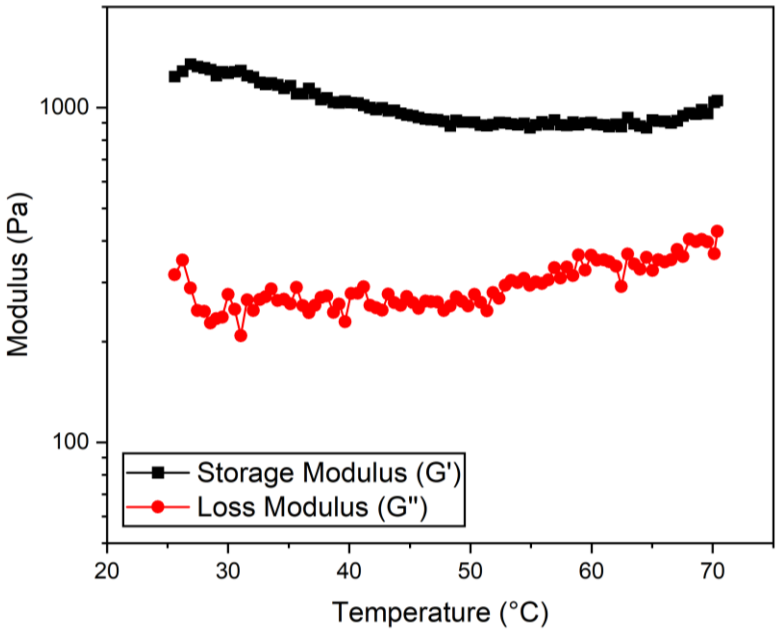

Figure S34 Dynamic temperature ramp (25 – 70 °C) of lithiated organogel made from dp-poly(**D-1**), PDBA, and LiTFSI (0.144 mol L^{-1}) with a polymer concentration of 0.287 mol L^{-1} and 0.50 equiv. of PDBA. Storage and loss moduli were recorded at a constant frequency of 1 Hz and a strain of 0.007 %.

6. FE-FEM

6.1 Cross section of 0.144 mol L^{-1} dp-poly(D-1), 0.25 PDBA equiv.

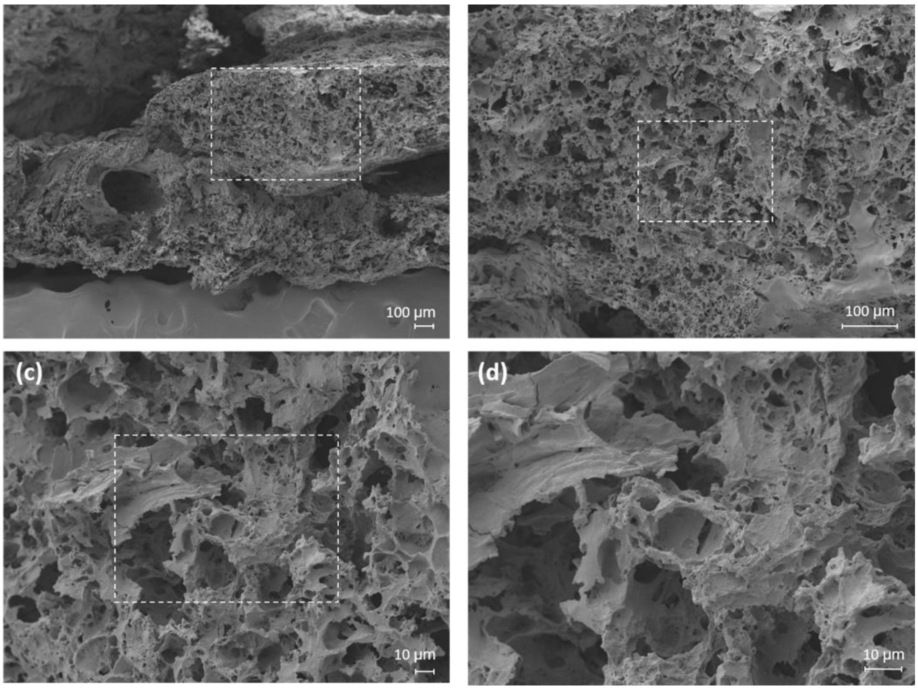

Figure S35 FE-SEM image of the cross-section of a lyophilised organogel made from 0.144 mol L^{-1} solution of dp-poly(**D-1**) in DMSO with 0.25 equiv. of PDBA, taken at 50, 150, 500, and 1000 times magnification in a), b), c), and d) respectively. The approximate area of magnification is indicated in each case.

6.2 Cross section of 0.144 mol L^{-1} dp-poly(D-1), 0.50 PDBA equiv.

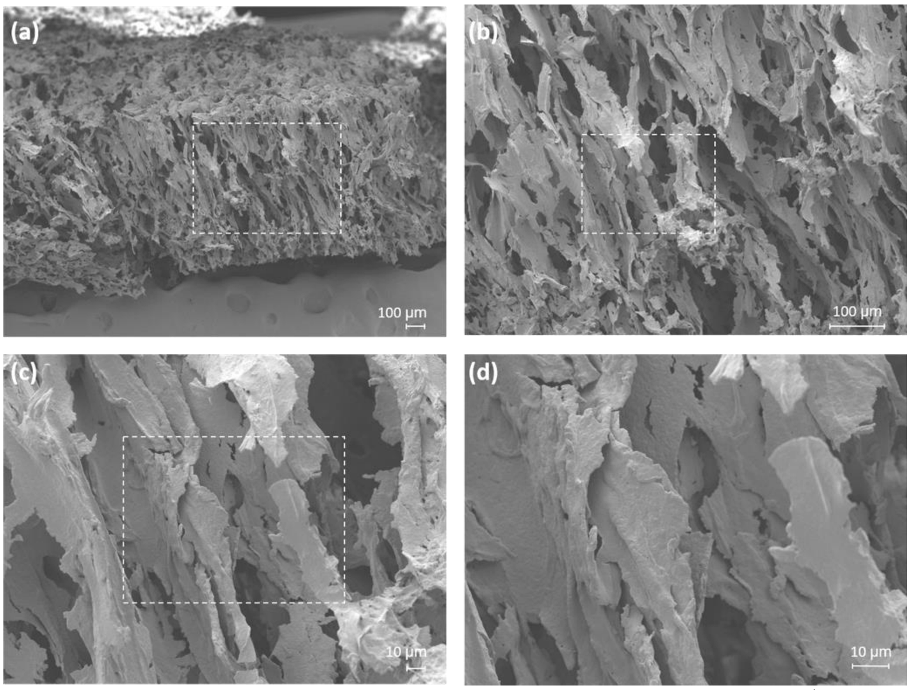

Figure S36 FE-SEM image of the cross-section of a lyophilised organogel made from 0.144 mol L^{-1} solution of dp-poly(**D-1**) in DMSO with 0.50 equiv. of PDBA, taken at 50, 150, 500, and 1000 times magnification in a), b), c), and d) respectively. The approximate area of magnification is indicated in each case.

6.3 Side view of 0.144 mol L^{-1} dp-poly(D-1), 0.50 PDBA equiv.

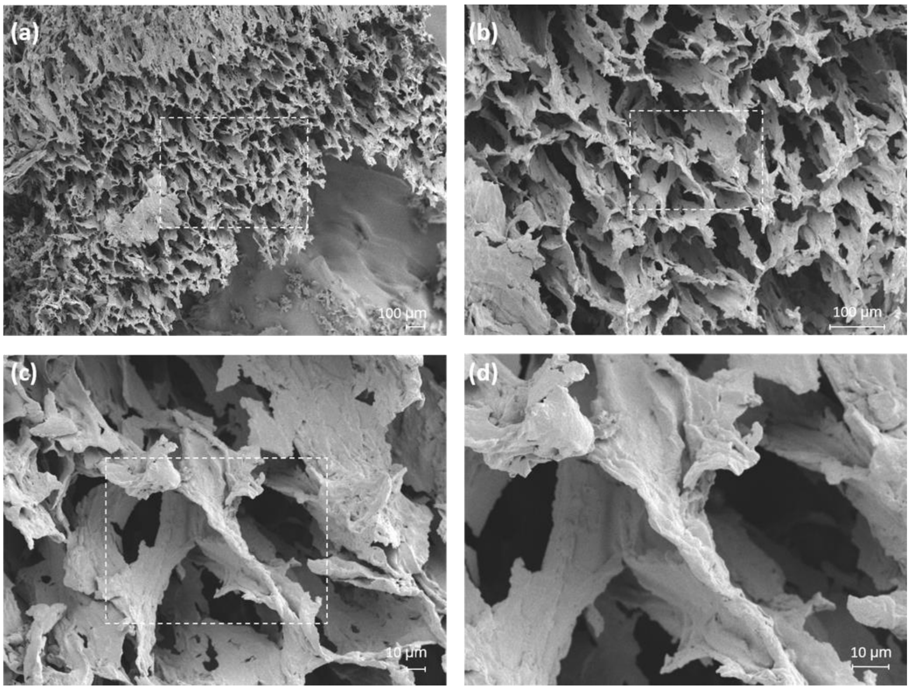

Figure S37 FE-SEM image of the side of a lyophilised organogel made from 0.144 mol L^{-1} solution of dp-poly(**D-1**) in DMSO with 0.50 equiv. of PDBA, taken at 50, 150, 500, and 1000 times magnification in a), b), c), and d) respectively. The approximate area of magnification is indicated in each case.

6.4 Cross section of 0.144 mol L^{-1} dp-poly(D-1), 1.00 PDBA equiv.

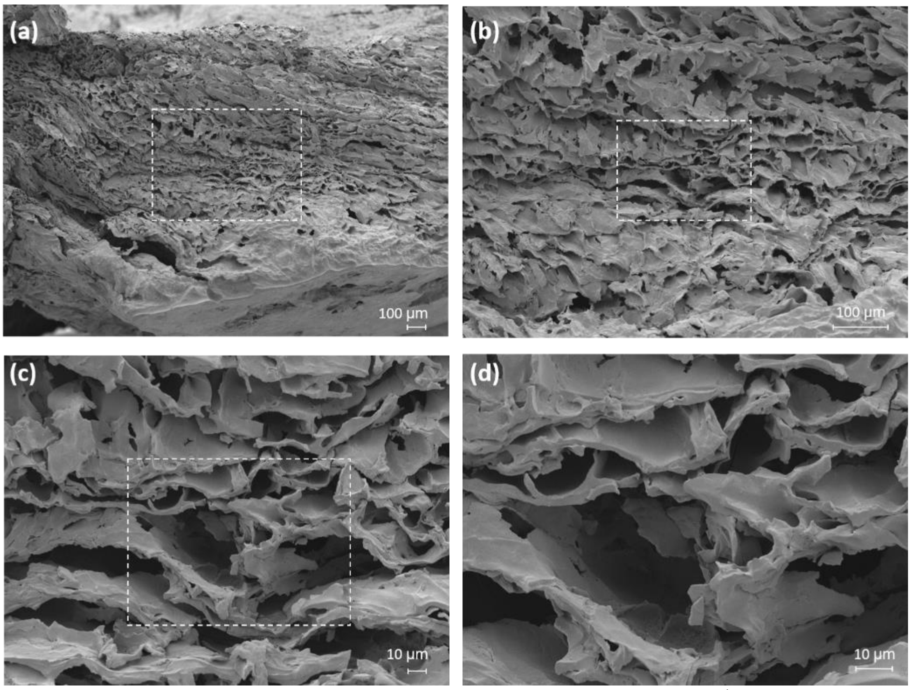

Figure S38 FE-SEM image of the side of a lyophilised organogel made from 0.144 mol L^{-1} solution of dp-poly(**D-1**) in DMSO with 1.00 equiv. of PDBA, taken at 50, 150, 500, and 1000 times magnification in a), b), c), and d) respectively. The approximate area of magnification is indicated in each case.

7. Thermal Characterisation

7.1 TGA Traces

7.1.1 Polyether, poly(D-1)

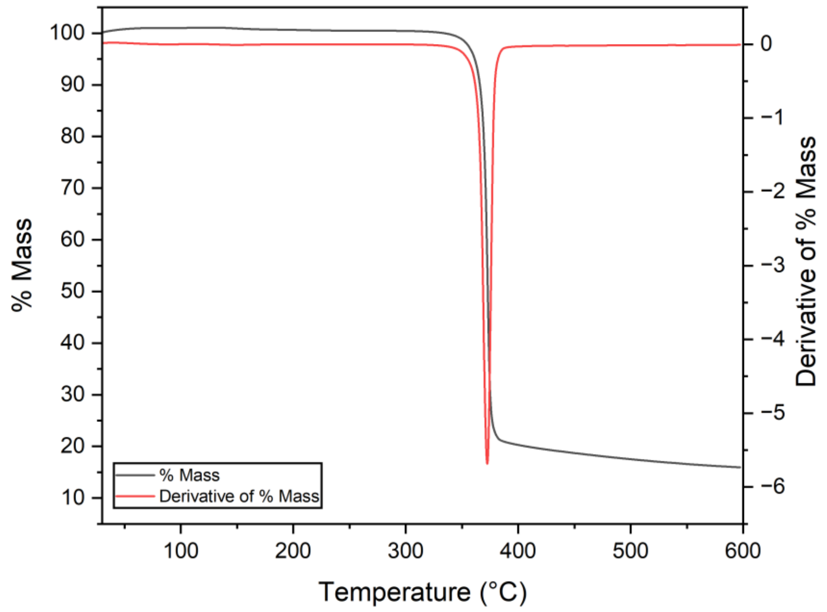

Figure S39 TGA trace of poly(D-1), showing $T_{d,max}$ = 372 °C and $T_{d,5\%}$ = 361 °C with 16% char remaining at 600 °C.

7.1.2 Deprotected polyether, dp-poly(D-1)

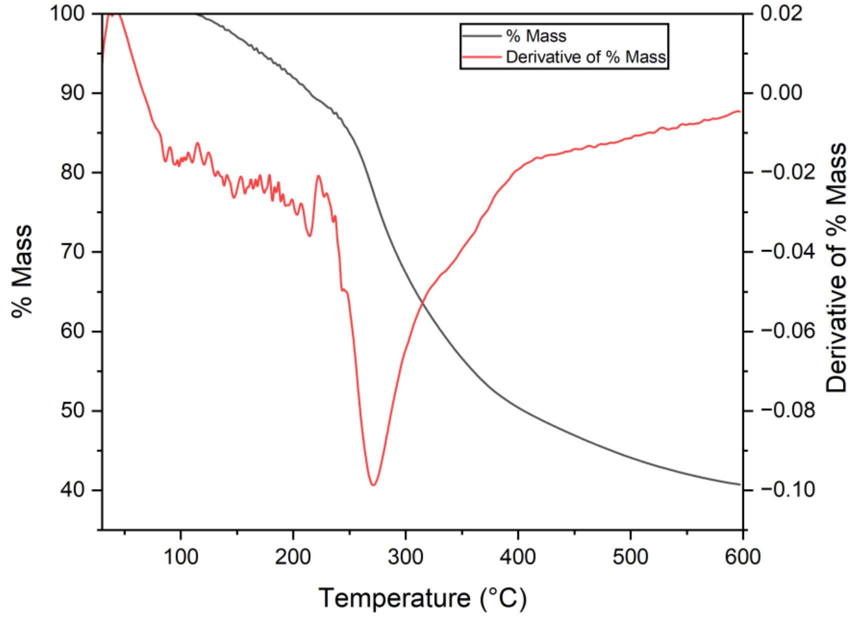

Figure S40 TGA trace of dp-poly(D-1), showing $T_{d,max}$ = 272 °C and $T_{d,5\%}$ = 136 °C with 40% char remaining at 600 °C.

7.1.3 0.144 mol L^{-1} dp-poly(D-1), 0.25 PDBA equiv.

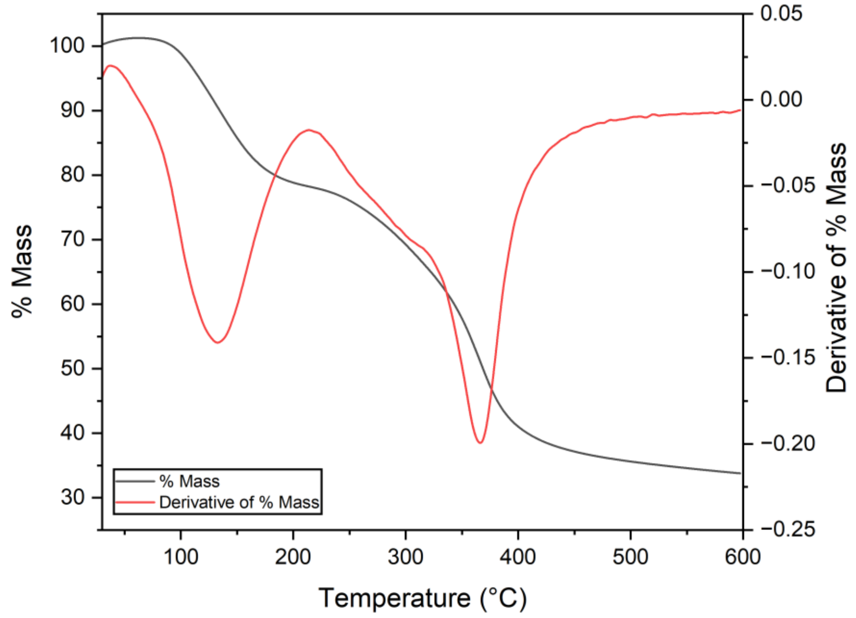

Figure S41 TGA trace of a lyophilised organogel made from dp-poly(**D-1**) and PDBA with a polymer concentration of 0.144 mol L^{-1} and 0.25 equiv. of PDBA , showing $T_{d,max}$ = 367.7 °C and $T_{d,5\%}$ = 116.8 °C with 34 % char remaining at 600 °C.

7.1.4 0.144 mol L^{-1} dp-poly(D-1), 0.50 PDBA equiv.

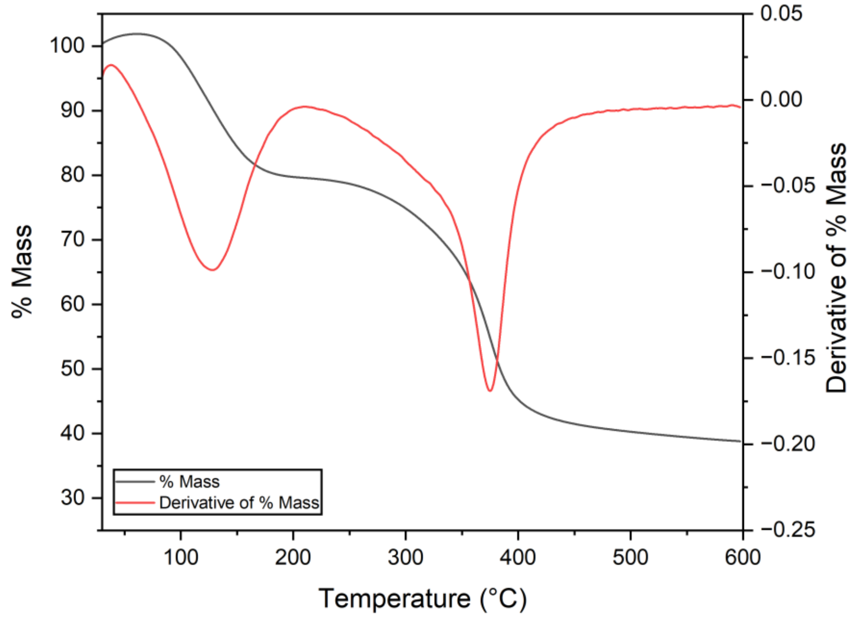

Figure S421 TGA trace of a dried organogel made from dp-poly(**D-1**) and PDBA with a polymer concentration of 0.144 mol L^{-1} and 0.50 equiv. of PDBA , showing $T_{d,max}$ = 375.5 °C and $T_{d,5\%}$ = 112.8 °C with 39% char remaining at 600 °C.

7.1.5 0.144 mol L^{-1} dp-poly(D-1), 1.00 PDBA equiv.

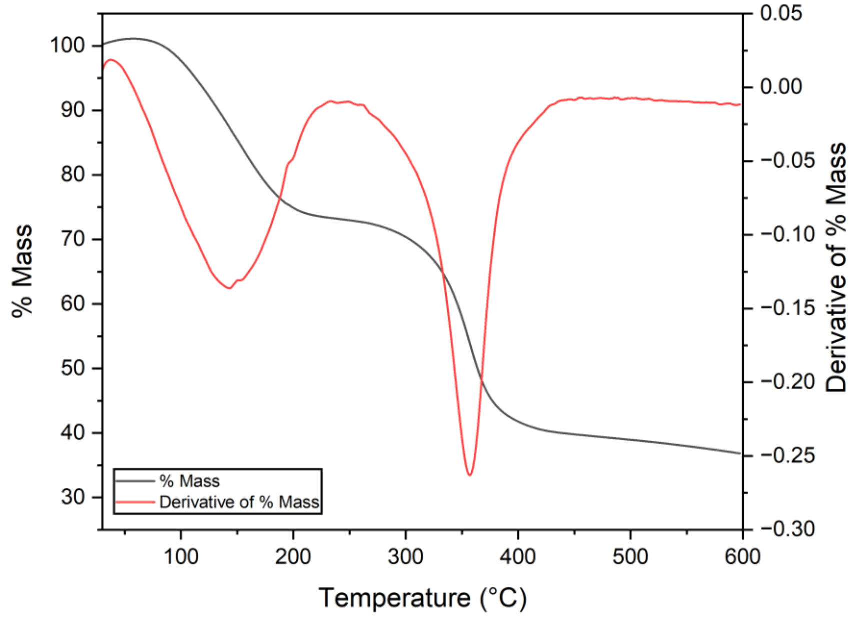

Figure S43 TGA trace of a dried organogel made from dp-poly(**D-1**) and PDBA with a polymer concentration of 0.144 mol L^{-1} and 1.00 equiv. of PDBA , showing $T_{d,max}$ = 356.5 °C and $T_{d,5\%}$ = 113.5 °C with 37% char remaining at 600 °C.

7.1.6 0.144 mol L^{-1} dp-poly(D-1), 0.25 PDBA equiv. nonlyophilized

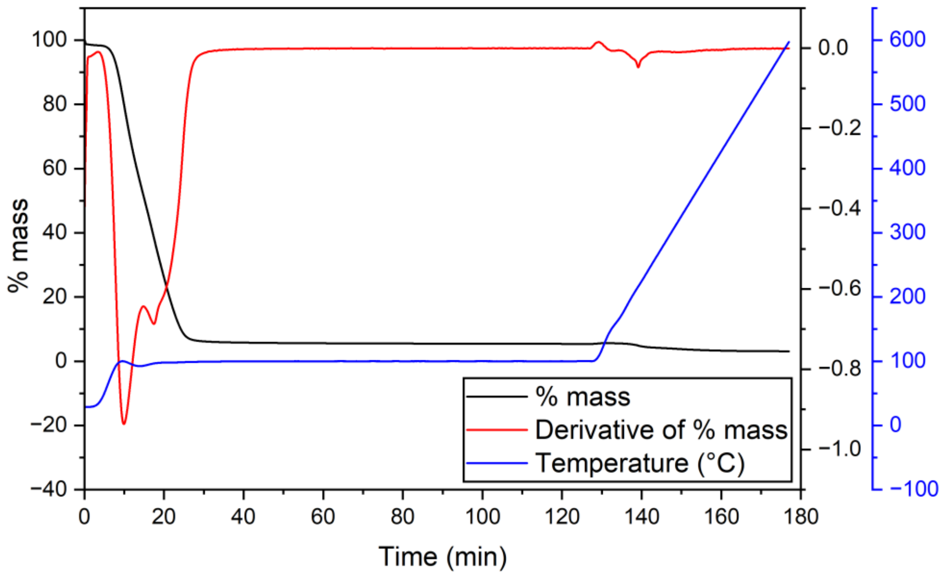

Figure S44 TGA trace of a nonlyophilised organogel made from dp-poly(**D-1**) and PDBA with a polymer concentration of 0.144 mol L^{-1} and 0.25 equiv. of PDBA , showing the removal of DMSO at 100 °C ($T_{d,max}$ = 100.0 °C, 74.9% mass loss) followed by polymer degradation ($T_{d,max}$ = 216.2 °C, 2% mass loss). 23% char remaining at 600 °C.

7.1.7 0.144 mol L^{-1} dp-poly(D-1), 0.50 PDBA equiv. nonlyophilized

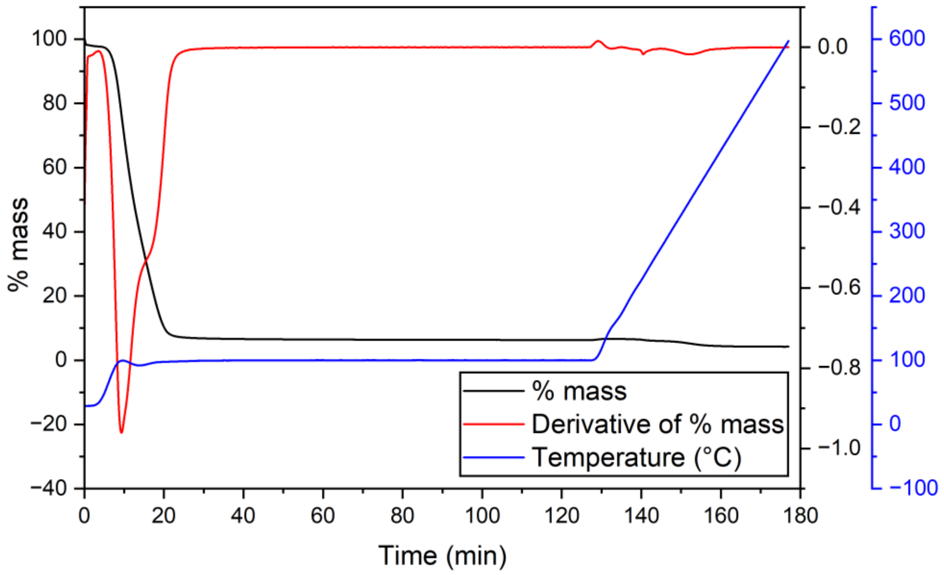

Figure S45 TGA trace of a nonlyophilised organogel made from dp-poly(**D-1**) and PDBA with a polymer concentration of 0.144 mol L^{-1} and 0.50 equiv. of PDBA, showing the removal of DMSO at 100 °C ($T_{d,max}$ = 99.3 °C, 68.3% mass loss) followed by polymer degradation ($T_{d,max}$ = 227.9 °C, 1.5% mass loss). 30% char remaining at 600 °C.

7.1.8 0.144 mol L^{-1} dp-poly(D-1), 1.00 PDBA equiv. nonlyophilized

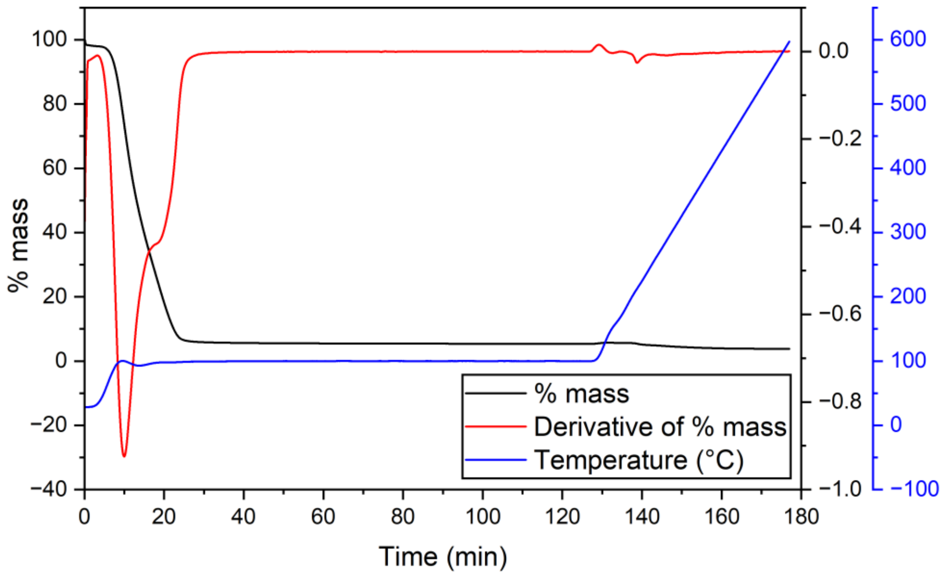

Figure S46 TGA trace of a nonlyophilised organogel made from dp-poly(**D-1**) and PDBA with a polymer concentration of 0.144 mol L^{-1} and 1.00 equiv. of PDBA, showing the removal of DMSO at 100 °C ($T_{d,max}$ = 100.1 °C, 71.2% mass loss) followed by polymer degradation ($T_{d,max}$ = 213.8 °C, 1.2% mass loss). 15% char remaining at 600 °C.

7.2 DSC Traces

7.2.1 Polyether, poly-(D-1)

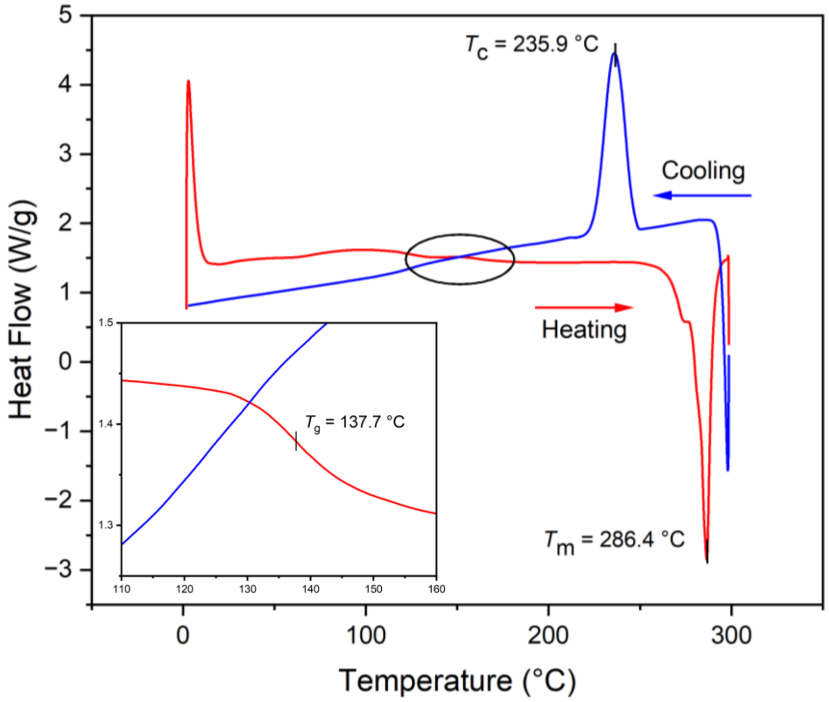

Figure S47 DSC trace collected at 20 °C min^{-1} showing the first heating and cooling cycle under nitrogen of poly(**D-1**), showing a crystallisation temperature, T_c of 235.9 °C, and a melting temperature, T_m of 286.4 °C. Inset shows the second heating and cooling cycle, and the glass transition temperature, T_g of 137.7 °C. Exothermic and endothermic events are indicated by positive and negative heat flows, respectively.

7.2.2 Deprotected Polyether, dp-poly(D-1)

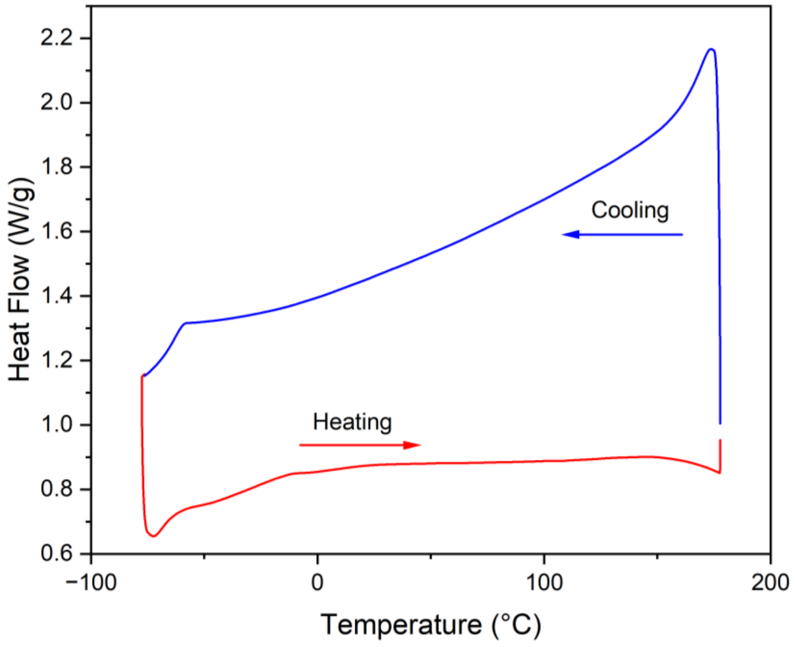

Figure S48 DSC trace collected at 20 °C min^{-1} showing the second heating and cooling cycle under nitrogen of dp-poly(**D-1**). T_c, T_m, and T_g are not observed. Exothermic and endothermic events are indicated by positive and negative heat flows, respectively.

7.2.3 0.144 mol L^{-1} dp-poly(D-1), 0.25 PDBA equiv.

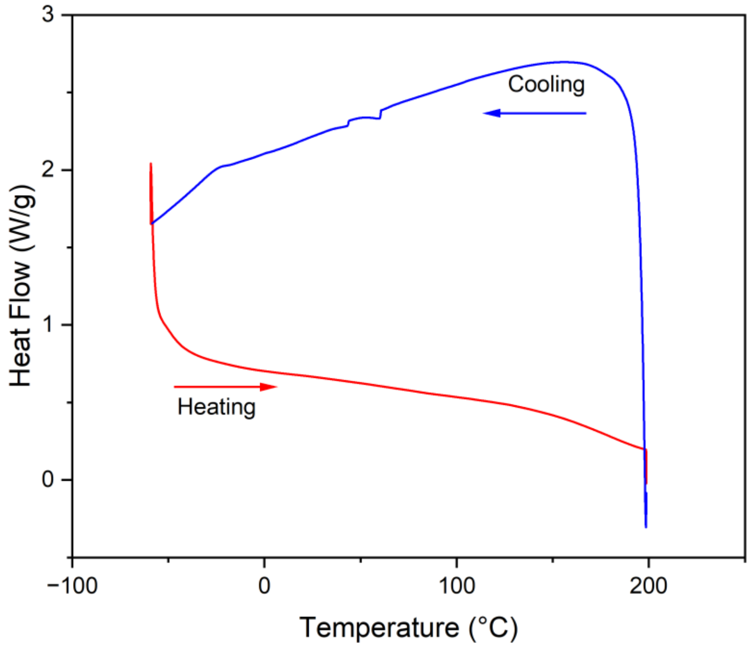

Figure S49 DSC trace collected at 20 °C min^{-1} showing the second heating and cooling cycle under nitrogen of a lyophilised organogel made from dp-poly(**D-1**) and PDBA with a polymer concentration of 0.144 mol L^{-1} and 0.25 equiv. of PDBA. T_g, T_c, and T_m are not observed. Exothermic and endothermic events are indicated by positive and negative heat flows, respectively.

7.2.4 0.144 mol L^{-1} dp-poly(D-1), 0.50 PDBA equiv.

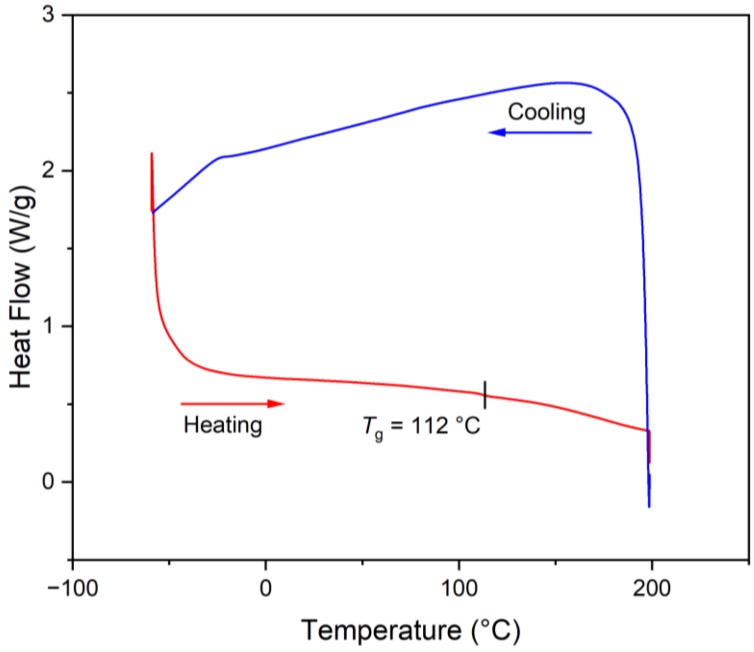

Figure S50 DSC trace collected at 20 °C min^{-1} showing the second heating and cooling cycle under nitrogen of a lyophilised organogel made from dp-poly(**D-1**) and PDBA with a polymer concentration of 0.144 mol L^{-1} and 0.50 equiv. of PDBA. T_c, and T_m are not observed. A slight T_g can be observed at 112 °C. Exothermic and endothermic events are indicated by positive and negative heat flows, respectively.

7.2.5 0.144 mol L^{-1} dp-poly(D-1), 1.00 PDBA equiv.

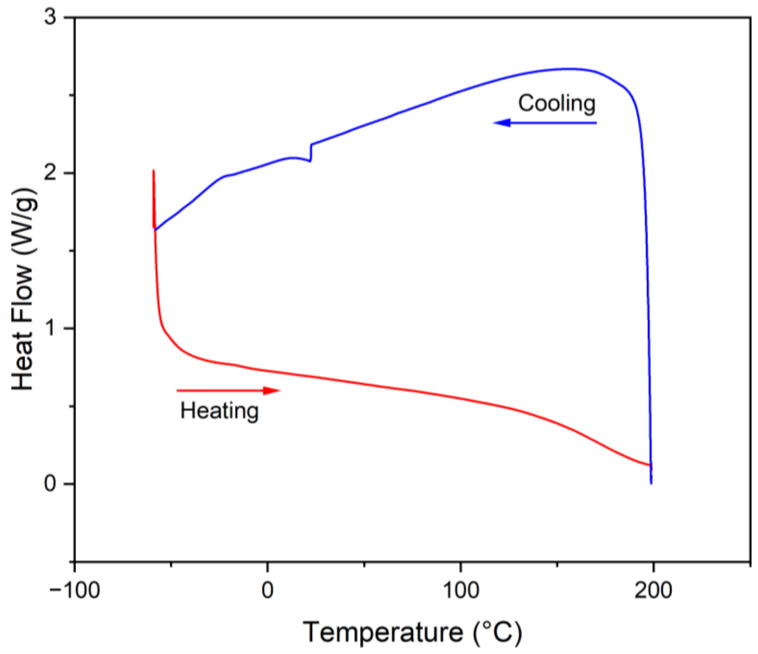

Figure S51 DSC trace collected at 20 °C min^{-1} showing the second heating and cooling cycle under nitrogen of a lyophilised organogel made from dp-poly(**D-1**) and PDBA with a polymer concentration of 0.144 mol L^{-1} and 1.00 equiv. of PDBA. T_c, T_m, and T_g are not observed. Exothermic and endothermic events are indicated by positive and negative heat flows, respectively.

8. Electrochemistry Impedance Spectroscopy (EIS)

8.1 Nyquist plots of 0.287 mol L^{-1} dp-poly(D-1), 0.50 PDBA equiv. with LiTFSI

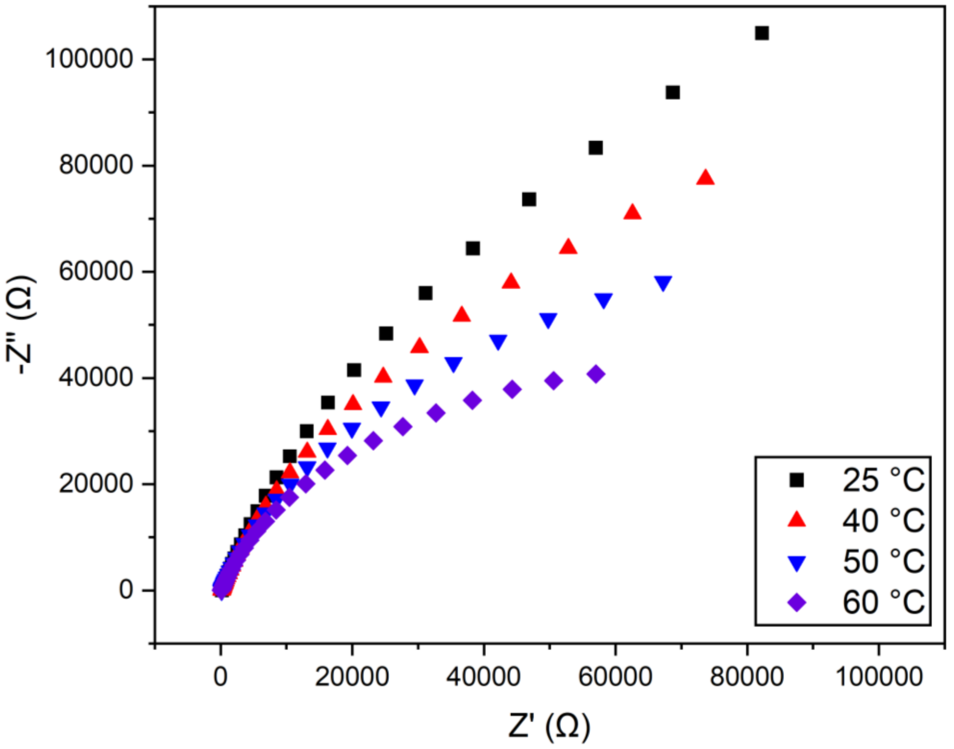

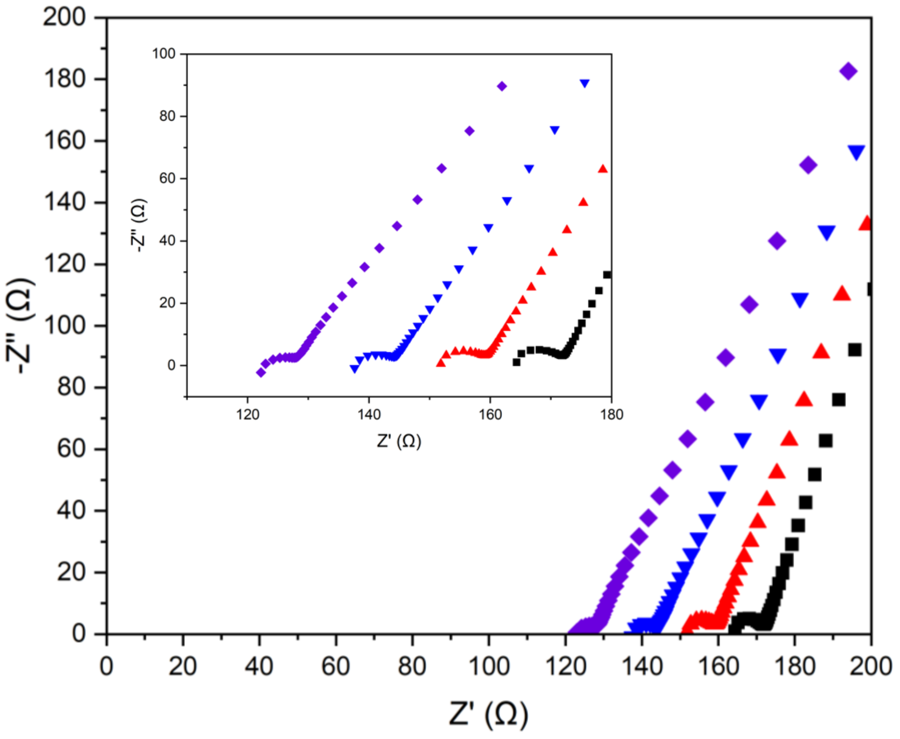

Figure S52 Nyquist plots, measured in the frequency range of 0.1 Hz to 0.5 MHz, of lithiated organogel made from dp-poly(**D-1**), PDBA, and LiTFSI (0.144 mol L^{-1}) with a polymer concentration of 0.287 mol L^{-1} and 0.50 equiv. of PDBA. TOP: whole Nyquist plots. BOTTOM: Zoom of Nyquist plots with 0 < Z' and Z'' < 200 Ω. BOTTOM INSET: Further zoom showing semi-circle characteristic of plots (100 < Z' < 180 Ω, and -10 < Z'' < 100 Ω).

8.2 Nyquist plots of 0.287 mol L^{-1} dp-poly(D-1), 0.50 PDBA equiv. without LiTFSI

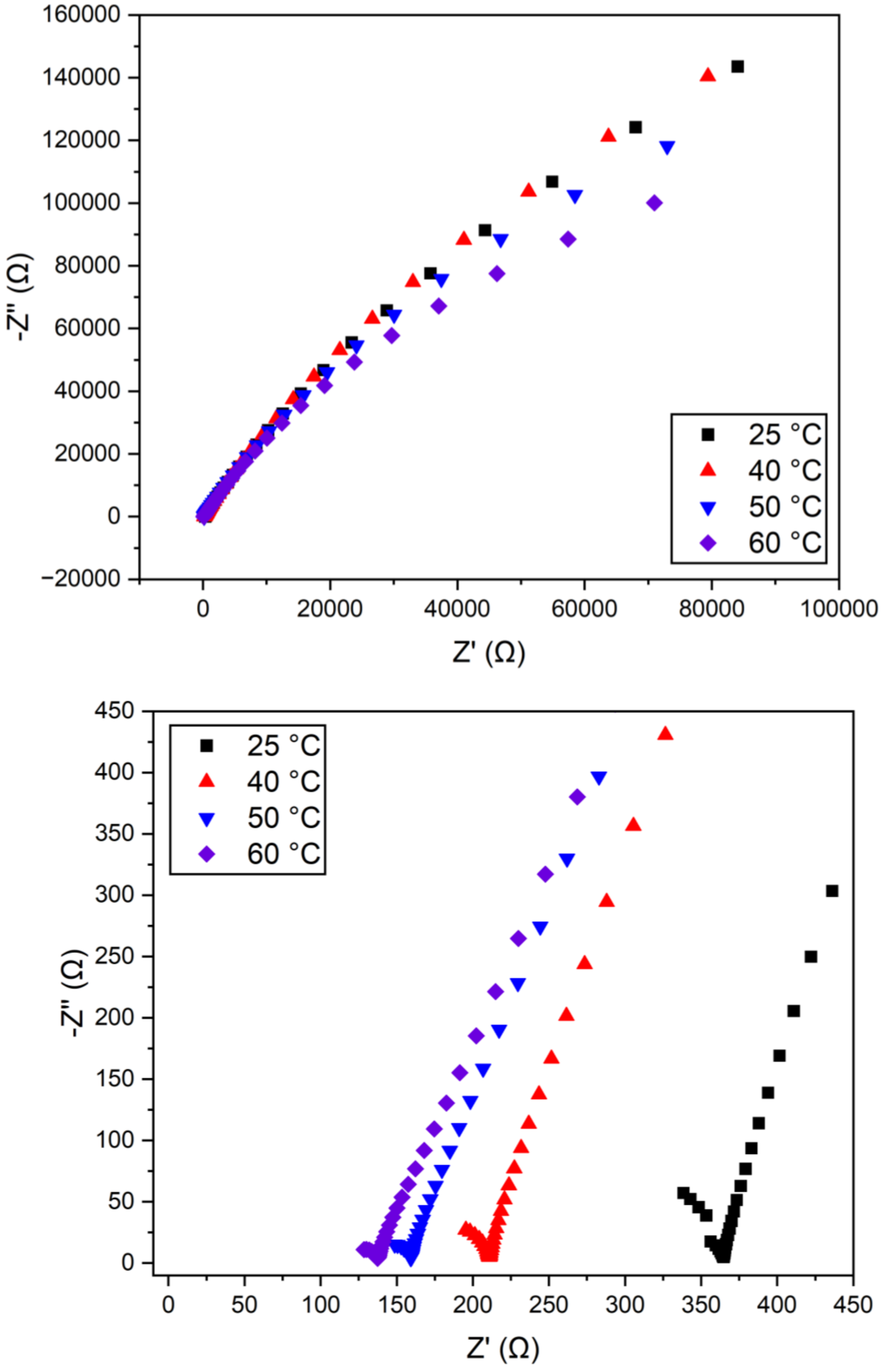

Figure S53 Nyquist plots, measured in the frequency range of 0.1 Hz to 0.5 MHz, of nonlithiated control organogel made from dp-poly(**D-1**), and PDBA with a polymer concentration of 0.287 mol L^{-1} and 0.50 equiv. of PDBA. TOP: whole Nyquist plots. BOTTOM: Zoom of Nyquist plots with 0 < Z' and Z'' < 450 Ω.

8.3 Temperature dependent conductivity of 0.287 mol L^{-1} dp-poly(D-1), 0.50 PDBA equiv. with LiTSFI

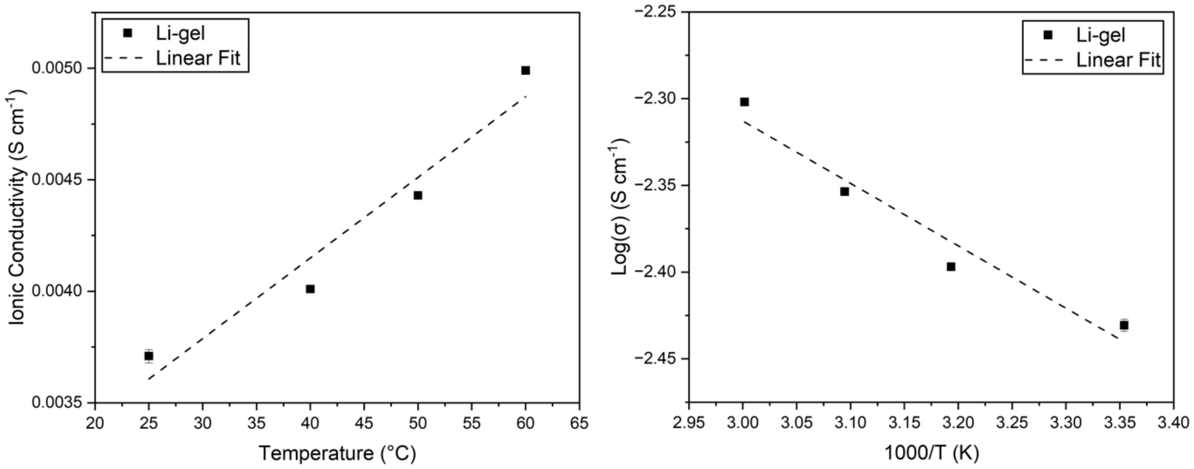

Figure S54 Temperature dependence of ionic conductivity (σ) for lithiated organogel made from dp-poly(**D-1**) and PDBA with a polymer concentration of 0.287 mol L^{-1} and 0.50 PDBA equiv. with 0.144 mol L^{-1} of LiTFSI.

Table S1 Bulk resistance (R_b) and ionic conductivity (σ) values of a lithiated organogel made from dp-poly(**D-1**), PDBA, and LiTFSI (0.144 mol L^{-1}) with a polymer concentration of 0.287 mol L^{-1} and 0.50 PDBA equiv. Values are averaged over five measurements. The thickness of the gel used was 3.21 mm.

Temp. (°C)	Temp. (K)	1000/T (K^{-1})	R_b (Ω)	σ (mS cm^{-1})	Log(σ)
25	298.15	3.3540	171.74	3.71	-2.4306
40	313.15	3.0193	159.11	4.01	-2.4318
50	323.15	3.0945	144.08	4.43	-2.3536
60	333.15	3.0017	127.66	4.99	-2.3019

8.4 Temperature dependent conductivity of 0.287 mol L^{-1} dp-poly(D-1), 0.50 PDBA equiv. without LiTSFI

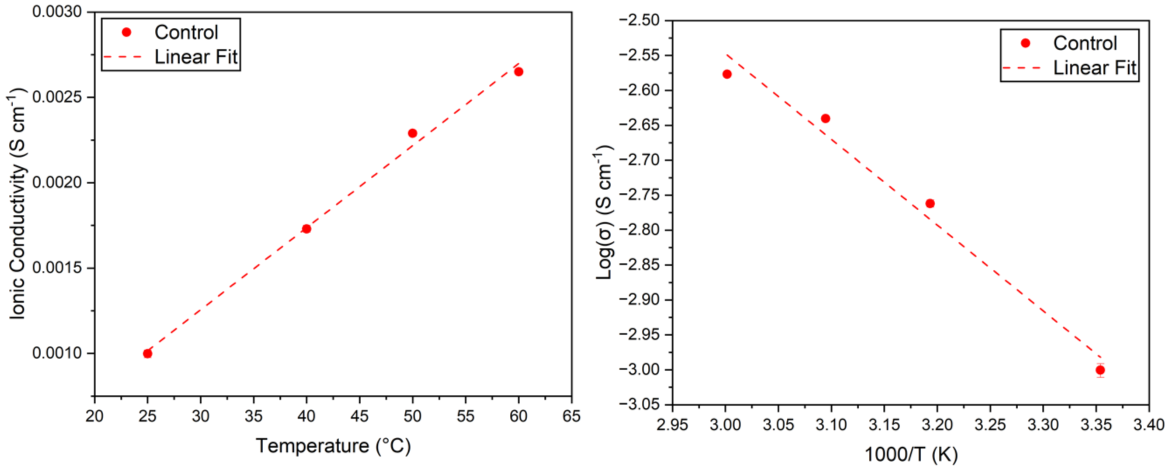

Figure S55 Temperature dependence of ionic conductivity (σ) for nonlithiated organogel made from dp-poly(**D-1**) and PDBA with a polymer concentration of 0.287 mol L^{-1} and 0.50 PDBA equiv..

Table S2 Bulk resistance (R_b) and ionic conductivity (σ) values of a nonlithiated organogel made from dp-poly(**D-1**) and PDBA with a polymer concentration of 0.287 mol L^{-1} and 0.50 PDBA equiv.. Values are averaged over five measurements. The thickness of the gel used was 1.83 mm.

Temp. (°C)	Temp. (K)	1000/T (K^{-1})	R_b (Ω)	σ (mS cm^{-1})	Log(σ)
25	298.15	3.3540	364.77	0.99	-3.00
40	313.15	3.0193	210.99	1.73	-2.76
50	323.15	3.0945	159.29	2.29	-2.64
60	333.15	3.0017	137.58	2.65	-2.58

8.5 Comparison with literature ionic conductivity values

Table S3 Comparison of ambient ionic conductivity (σ) values of this work (lithiated organogel made from dp-poly(**D-1**), PDBA, and LiTFSI (0.144 mol L^{-1}) with a polymer concentration of 0.287 mol L^{-1} and 0.50 PDBA equiv.) and literature examples.

Publication	Salt [Conc.] (mol L^{-1})	Electrolyte	Temp. (°C)	σ (mS cm^{-1})	t_{Li+}
**This work**	**LiTFSI [0.144]**	**DMSO**	**25**	**3.71**	**0.90**
Ma *et al.*[1]	$LiClO_4$ [1.000]	DMF	25	2.33	0.60
Dai *et al.*[2]	LiTFSI [1.000]	1:1 EC:DMC	30	0.84	0.76
Chen *et al.*[3]	$LiClO_4$ [0.100]	H_2O	25	4.50	/
Deng *et al.*[4]	LiBAMB [0.500]	GBL/PC/EC	25	1.47	0.89
Shim *et al.*[5]	LiTFSI [1.000]	1:1 EC:DEC	30	4.20	0.68
Humbeck *et al.*[6]	LiOH	PC	28	2.70	0.93
Wang *et al.*[7]	PLTB [1.000]	1:1 EC:DMC	25	0.50	0.91
Sun *et al.*[8]	LiBAMB	1:1 EC:DMC	25	7.90×10^{-3}	/
Zhou *et al.*[9]	PAMPSLi	EC	20	0.25	/
Zhu *et al.*[10]	Li_2CO_3	PC	25	6.11×10^{-3}	/
Alvarez-Tirado *et al.*[11]	nBuLi	Tetraglyme	25	0.71	0.85
Zeng *et al.*[12]	LiBMAB/LiTFSI [1.000]	1:1 EC:DEC	32	1.03	0.65

DMSO = Dimethyl sulfoxide, DMF = Dimethylformamide, EC = Ethylene carbonate, DMC = Dimethyl carbonate, GBL = Gamma-butyrolactone, PC = Propylene carbonate, DEC = Diethylene carbonate

8.6 Linear Sweep Voltammetry

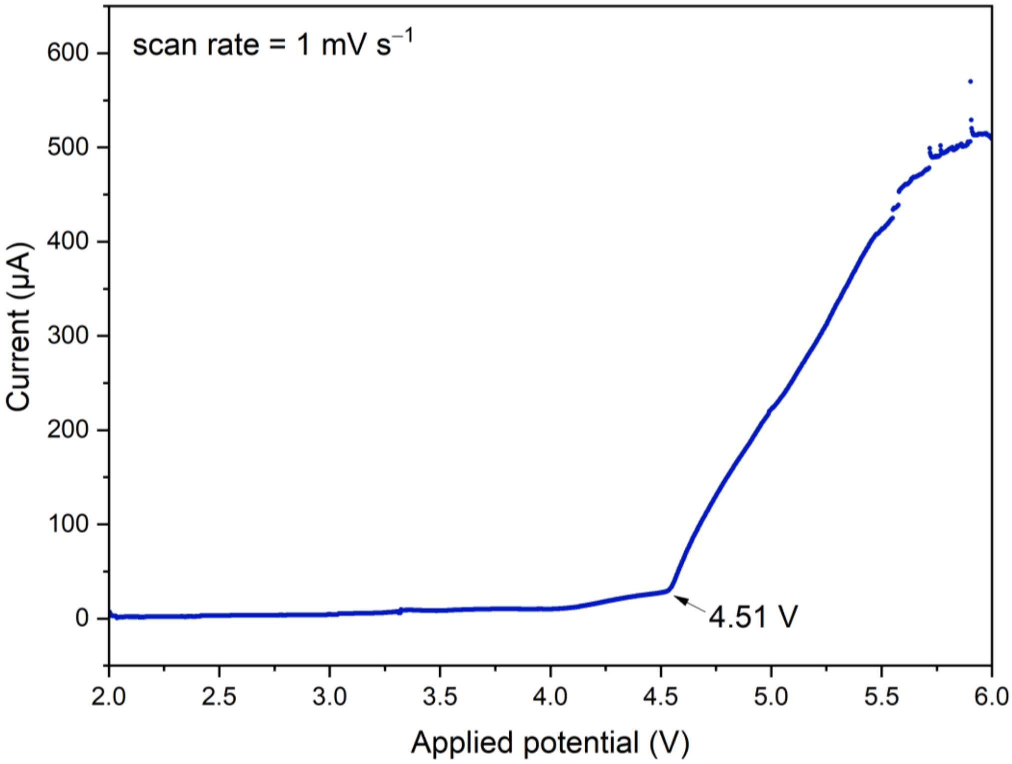

Figure S56 Linear sweep voltammetry (LSV) of a lithiated organogel (made from dp-poly(**D-1**) and PDBA with a polymer concentration of 0.287 mol L^{-1} and 0.50 PDBA equiv. with 0.144 mol L^{-1} of LiTFSI.) in a Li|SPE|SS cell using a lithium counter/reference electrode at 25 °C. The open-cell voltage (OCV) was first determined and then the voltage swept from the OCV to +6 V with a scan rate of 1 mV s^{-1}.

8.7 Lithium Transference Number, t_{Li+}

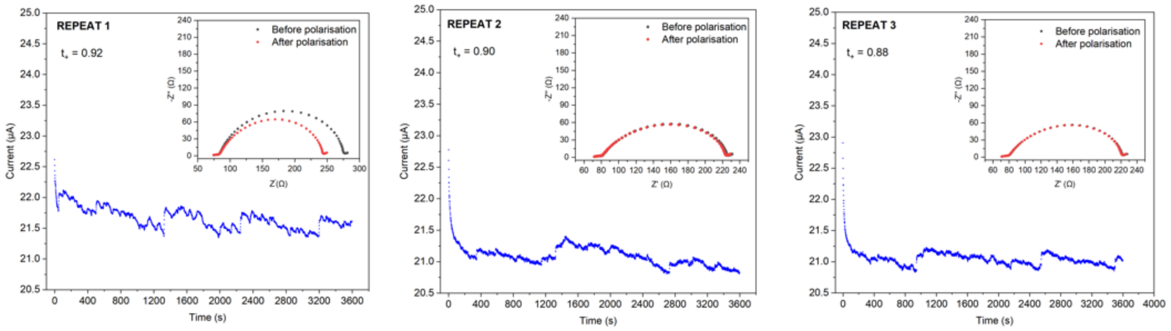

Figure S57 Chronoamperometry and Nyquist plots (inlayed) obtained for the determination of t_{Li+} of **Li-gel**. A 10 mV applied polarisation voltage was used for the chronoamperometry in a symmetrical Li|GPE|Li cell. The Nyquist plots were obtained by EIS in the frequency range of 0.1 Hz to 1 MHz.

Table S4 Data used for the determination of the lithium transference number (t_{Li+}). Average t_{Li+} = 0.90 ± 0.011516.

Repeat	I_0 (A)	I_{ss} (A)	$R_{b,0}$ (Ω)	$R_{b,ss}$ (Ω)	ΔV	t_{Li+}
1	2.27E-05	2.17E-05	166.18	162.94	0.01	0.92
2	2.27e-05	2.12e-05	146.76	144.38	0.01	0.90
3	2.29e-05	2.10e-05	143.63	142.10	0.01	0.88

9. References

1. Ma, L.; Jin, M.; Yan, C.; Guo, H.; Ma, X., Gel Polymer Electrolyte with Anion-Trapping Boron Moieties via One-Step Synthesis for Symmetrical Supercapacitors. *Macromol. Mater. Eng.* **2020**, *305* (7), 1900807.
2. Dai, K.; Ma, C.; Feng, Y.; Zhou, L.; Kuang, G.; Zhang, Y.; Lai, Y.; Cui, X.; Wei, W., A borate-rich, cross-linked gel polymer electrolyte with near-single ion conduction for lithium metal batteries. *J. Mater. Chem. A* **2019**, *7* (31), 18547-18557.
3. Chen, Q.; Shi, Y.; Sheng, K.; Zheng, J.; Xu, C., Dynamically Cross-Linked Hydrogel Electrolyte with Remarkable Stretchability and Self-Healing Capability for Flexible Electrochromic Devices. *ACS Appl. Mater. Interfaces* **2021**, *13* (47), 56544-56553.
4. Deng, K.; Wang, S.; Ren, S.; Han, D.; Xiao, M.; Meng, Y., Network type sp3 boron-based single-ion conducting polymer electrolytes for lithium ion batteries. *J. Power Sources* **2017**, *360*, 98-105.
5. Shim, J.; Lee, J. S.; Lee, J. H.; Kim, H. J.; Lee, J.-C., Gel Polymer Electrolytes Containing Anion-Trapping Boron Moieties for Lithium-Ion Battery Applications. *ACS Appl. Mater. Interfaces* **2016**, *8* (41), 27740-27752.
6. Van Humbeck, J. F.; Aubrey, M. L.; Alsbaiee, A.; Ameloot, R.; Coates, G. W.; Dichtel, W. R.; Long, J. R., Tetraarylborate polymer networks as single-ion conducting solid electrolytes. *Chem. Sci.* **2015**, *6* (10), 5499-5505.
7. Wang, X.; Liu, Z.; Kong, Q.; Jiang, W.; Yao, J.; Zhang, C.; Cui, G., A single-ion gel polymer electrolyte based on polymeric lithium tartaric acid borate and its superior battery performance. *Solid State Ionics* **2014**, *262*, 747-753.
8. Sun, X.-G.; Liu, G.; Xie, J.; Han, Y.; Kerr, J. B., New gel polyelectrolytes for rechargeable lithium batteries. *Solid State Ionics* **2004**, *175* (1), 713-716.
9. Zhou, F.; MacFarlane, D. R.; Forsyth, M., Boroxine ring compounds as dissociation enhancers in gel polyelectrolytes. *Electrochim. Acta* **2003**, *48*, 1749-1758.
10. Zhu, Y. S.; Wang, X. J.; Hou, Y. Y.; Gao, X. W.; Liu, L. L.; Wu, Y. P.; Shimizu, M., A new single-ion polymer electrolyte based on polyvinyl alcohol for lithium ion batteries. *Electrochim. Acta* **2013**, *87*, 113-118.
11. Alvarez-Tirado, M.; Guzman-Gonzalez, G.; Vauthier, S.; Cotte, S.; Gueguen, A.; Castro, L.; Mecerreyes, D., Designing Boron-Based Single-Ion Gel Polymer Electrolytes for Lithium Batteries by Photopolymerization. *Macromol. Chem. Phys.* **2022**, *223* (8), 2100407.
12. Zeng, X.; Dong, L.; Fu, J.; Chen, L.; Zhou, J.; Zong, P.; Liu, G.; Shi, L., Enhanced interfacial stability with a novel boron-centered cross-linked hybrid polymer gel electrolytes for lithium metal batteries. *Chem. Eng. J.* **2022**, *428*, 131100.
